# Rapid characterization of adeno-associated virus (AAV) capsid proteins using microchip ZipChip CE-MS

**DOI:** 10.1007/s00216-023-05097-5

**Published:** 2023-12-15

**Authors:** Josh Smith, Sara Carillo, Aditya Kulkarni, Erin Redman, Kate Yu, Jonathan Bones

**Affiliations:** 1 Characterisation and Comparability Laboratory, The National Institute for Bioprocessing Research and Training, Foster Avenue, Mount Merrion, A94 X099 Co. Dublin Ireland; 2 908 Devices Inc., 645 Summer Street #201, Boston, MA 02210 USA; 3 908 Devices Inc., 511 Davis Dr Suite 450, Morrisville, NC 27560 USA; 4 School of Chemical and Bioprocess Engineering, University College Dublin, Belfield, Dublin 4 D04 V1W8 Ireland

**Keywords:** Adeno-associated virus, Gene therapy, Microchip CE-MS, Product quality monitoring, Characterization, Post translation modifications

## Abstract

Adeno-associated viruses (AAVs) are viral vectors used as delivery systems for gene therapies. Intact protein characterization of AAV viral capsid proteins (VPs) and their post-translational modifications is critical to ensuring product quality. In this study, microchip-based ZipChip capillary electrophoresis-mass spectrometry (CE-MS) was applied for the rapid characterization of AAV intact VPs, specifically full and empty viral capsids of serotypes AAV6, AAV8 and AAV9, which was accomplished using 5 min of analysis time. Low levels of dimethyl sulfoxide (4%) in the background electrolyte (BGE) improved MS signal quality and component detection. A sensitivity evaluation revealed consistent detection of VP proteoforms when as little as 2.64 × 10^6^ viral particles (≈26.4 picograms) were injected. Besides the traditional VP proteoforms used for serotype identification, multiple VP3 variants were detected, including truncated VP3 variants most likely generated by leaky scanning as well as unacetylated and un-cleaved VP3 proteoforms. Phosphorylation, known to impact AAV transduction efficiency, was also seen in all serotypes analysed. Additionally, low abundant fragments originating from either N- or C-terminus truncation were detected. As the aforementioned VP components can impact product quality and efficacy, the ZipChip’s ability to rapidly characterize them illustrates its strength in monitoring product quality during AAV production.

**Graphical Abstract:**

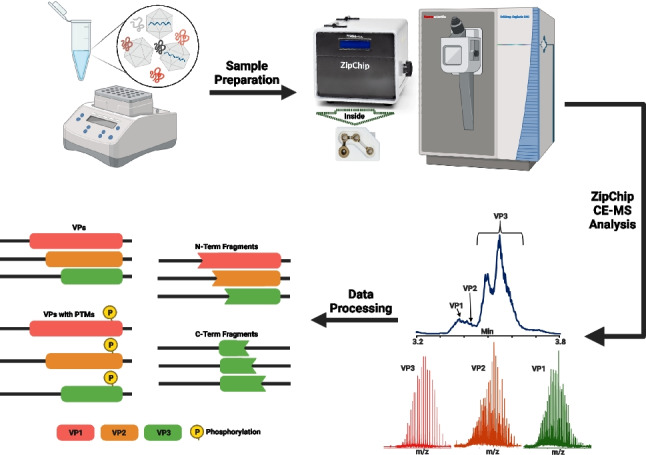

**Supplementary Information:**

The online version contains supplementary material available at 10.1007/s00216-023-05097-5.

## Introduction

Adeno-associated viruses (AAVs) are icosahedral capsids comprised of 60 total copies of three viral proteins (VPs), VP1, VP2, and VP3, in an approximate 1:1:10 ratio, respectively [1]. VP3 is the smallest of the three VPs, with VP2 containing the entire sequence of VP3 as the C-terminal sequence and VP1 containing the entirety of VP2 as its C-terminal sequence [2]. These capsids contain single-strand (ss) DNA of approximately 4.7 kb that delivers the genetic payload to target cells. There is increasing interest in AAVs as gene therapy vectors because of their highly effective delivery mechanisms, low cytotoxicity, and minimal immunogenicity. Additionally, the variety of serotypes, each having different tropisms, provides the ability to target specific cell types and organs [2–5]. Currently a handful of AAV therapy products are on the market, receiving conditional or full approval by either the US Food and Drug Administration (USFDA) or the European Medicines Agency (EMA): Luxturna® (approval date: 2017, serotype: AAV2, disease: retinal dystrophy), Zolgensma® (2019, AAV9, spinal muscular dystrophy), Hemgenix® (2022, AAV5, haemophilia B), Upstaza™ (2022, AAV2, aromatic L-amino acid decarboxylase deficiency), Elevidys (2023, AAVrh74, Duchenne muscular dystrophy), and Roctavian™ (2023, AAV5, haemophilia A).

The increase in approved AAV-based gene therapies in just the last 2 years along with the over 100 clinical trials currently ongoing (https://clinicaltrials.gov/) demonstrates that investigation into these gene therapies is continuously progressing. Monitoring AAV vector quality is crucial for ensuring product safety and efficacy. A key aspect of this is monitoring changes in post-translational modifications (PTMs) on the capsid VPs. PTMs on said VPs are known to occur during production and storage, and can have an influence on product stability, infectivity, and transduction efficiency [2, 6–9]. They also are seen varying between production lots highlighting the importance of batch-to-batch monitoring [2]. Additionally, final product yields for full AAV capsids are low due to the high levels of empty or partially filled capsids that are generated during production and which need to be removed during downstream processing [10]. Therefore, having highly sensitive AAV characterization platforms is critical to minimize the amount of sample needed for quality control (QC) testing.

Capillary electrophoresis (CE) is a fast and highly sensitive analytical technique commonly used for characterization of protein biologics [11]. CE analysis requires minimum sample, making it an ideal platform for the characterization of low yield products like AAVs. CE can be considered the standard platform for VP separations although the poor compatibility with mass spectrometry (MS) detection directed method development towards LC–MS approaches. While reversed phase (RP) and hydrophilic interaction liquid chromatography (HILIC) are the techniques of choice and can be easily hyphenated with MS, long method optimization is usually required to obtain good sensitivity and resolution [12]. In the past, CE has often struggled with compatibility to other highly informative analytical platforms such as MS; however, great strides have been made in creating coupled CE-MS platforms that can maximize the potential of their respective analytical capabilities [11]. One such platform is microchip CE-MS. Microchip CE-MS has emerged as a powerful technique for the characterization of biologics because of its high throughput, high sensitivity, rapid analysis time, and low sample consumption [13]. However, application of microchip CE-MS platforms to the characterization of AAVs has not yet been widely explored outside of Zhang et al. demonstrating the utilization of microchip CE-MS for the identification of AAV serotypes [14].

Here, we describe how the microchip ZipChip CE-MS platform can be utilized for the rapid characterization of AAV capsid proteins. We outline the steps taken to maximize detection of low abundant proteoforms and discuss how the use of low levels of dimethyl sulfoxide (DMSO) in the background electrolyte solution (BGE) improves VP detection and identification. A limit of detection (LoD) study was then performed to demonstrate how ZipChip CE-MS is a powerful platform even at low sample concentrations. Finally, we apply the ZipChip platform to the analysis of empty and full capsids from multiple serotypes to illustrate that it can perform not only AAV serotype identification, but also the detection of VP variants and fragments, as well as proteoforms containing PTMs that can impact product efficacy and safety.

## Materials and methods

### Reagents and materials

All reagents and solvents used were ACS reagent grade or better. Full AAV capsid serotypes derived from *Spodoptera frugiperda* Sf9 cells and produced using the cytomegalovirus-green fluorescent protein (CMV-GFP) construct were purchased from Virovek (Hayward, CA, USA) along with their empty capsid counterparts collected from the same production batch. Serotypes purchased were AAV6, AAV8, and AAV9. ZipChip High Resolution (HR) chips (Cat# 810–00140) and ZipChip Peptides Kits (Cat# 810–00167) containing ZipChip Peptides BGE were obtained from 908 Devices (Boston, MA, USA). Thermo Scientific™ SMART Digest™ pepsin kit was obtained from Thermo Fisher Scientific (Sunnyvale, CA, USA). Optima™ LC–MS grade acetonitrile (ACN), Thermo Scientific™ UHPLC-MS grade water, formic acid (FA), Tris(2-carboxyethyl)phosphine hydrochloride (TCEP), and LC–MS grade DMSO were sourced from Fisher Scientific (Dublin, Ireland).

### ZipChip CE-MS analysis for intact VPs

The ZipChip CE Ti interface with nano-ESI ion source (908 Devices, Boston, MA, USA) was installed following the vendor’s instructions. The Ti interface was attached to the front end of an Orbitrap Exploris 240 Mass Spectrometer (Thermo Scientific, Bremen, Germany), and an HR chip with a 22-cm-long separation channel was used. Before analysis, each AAV sample was diluted fivefold with peptide BGE (5 μL of sample + 20 μL of peptide BGE) and incubated on a Thermomixer for 15 min at 37 °C shaking at 500 rpm. Meanwhile, the HR Chip was primed with Peptides BGE containing 4% v/v DMSO. Immediately after incubation, 20 μL of incubated AAV sample was loaded manually into the sample well, which had previously been emptied after BGE prime. The AAV analysis was performed with a 5-min run time per injection. For each of the samples, 5 separate injections were performed per 20 μL sample load. The sample well was rinsed, and a BGE refresh was performed after all injections of a sample were run.

All data acquisition was performed using Thermo Scientific™ Chromeleon™ Chromatography Data System software version 7.3.1 (Thermo Scientific, Germering, Germany). Acquisition was triggered using the ZipChip software used to control ZipChip output. The following ZipChip CE settings were applied to analysis: an injection volume of 5.5 nL, a field strength of 500 V/cm, and a pressure assist start time of 0.5 min.

Global MS data parameters utilized on the Orbitrap Exploris 240 were as follows: intact protein was selected for application mode, low pressure was selected for pressure mode, liquid chromatography was selected for infusion mode, the expected peak width was 10 s, advanced peak determination was selected, the default charge state was 35, and internal mass calibration was off. The ion source properties registered the CE source as an ESI ion source with a static spray voltage and a positive ion capillary voltage of 0 V was used. A static gas mode was utilized with the sheath gas at 2 arbitrary units (au), auxiliary gas at 0 au, and a sweep gas at 0 au. The ion transfer tube temperature was set at 200 °C.

The MS scan parameters used are also as follows: Full-scan MS1 analysis was performed in positive ion mode with a scan range of *m/z* 740–2,000. Samples were analysed with an Orbitrap resolution of 15,000 (15 K) at m/z 200, the RF lens was set at 125%, the normalized AGC target was 50%, the maximum injection time was 200 ms, and the number of microscans was set to 2. Data was collected in profile mode. To assist in desolvation, the source fragmentation parameter was set to 35 V.

Data processing was performed using the Intact Mass Analysis experiment within Biopharma Finder Version 5.1 (BPF 5.1). All 5 injections of a sample were processed together using the multiconsensus option in BPF 5.1. Source spectra were selected using the Sliding Windows feature and deconvoluted using the ReSpect deconvolution algorithm. VP1 sequences for AAV6 (ID: AAB95450.1), AAV8 (ID: AAN03857.1), and AAV9 (ID: AAS99264.1) were obtained from the GeneBank® genetic sequence database accessed through The National Center for Biotechnology website (https://www.ncbi.nlm.nih.gov/genbank/). The VP2, VP3, and all protein fragment sequences for each serotype were generated from their respective VP1 sequences. Detected components were searched against the generated sequences for identification. All identifications were filtered so that they were found in at least 3 replicate injections, had a quality score ≥ 35, and had a relative abundance ≥ 0.05, unless otherwise noted. Full search parameters are described in Supplementary Table 1 (Table S1).

### Peptide mapping

Peptide mapping was performed to help verify the presence of identified VP PTMs, VP variants and VP fragments. Digestion of each empty and full capsid sample for peptide mapping was performed using a SMART Digest™ magnetic bead bulk pepsin kit (Thermo Scientific) and followed a slightly modified version of the protocol previously described by Guapo et al. [9] (Supplementary Information (SI) 1). Peptide mapping was performed in technical triplicate using a Vanquish Neo Ultra-High-Performance Liquid Chromatography (UHPLC) platform coupled to an Orbitrap Exploris 480 MS (Thermo Scientific, Bremen, Germany) following a modified version of the procedure described in Guapo et al*.* [9] (SI 2). Peptide identification and relative PTM quantitation was performed using BioPharma Finder™ (BPF) Version 5.1 (Thermo Scientific, San Jose, CA, USA) (SI 3 and Table S2).

## Results and discussion

### Enhancement of MS spectra using DMSO

**Fig. 1:**
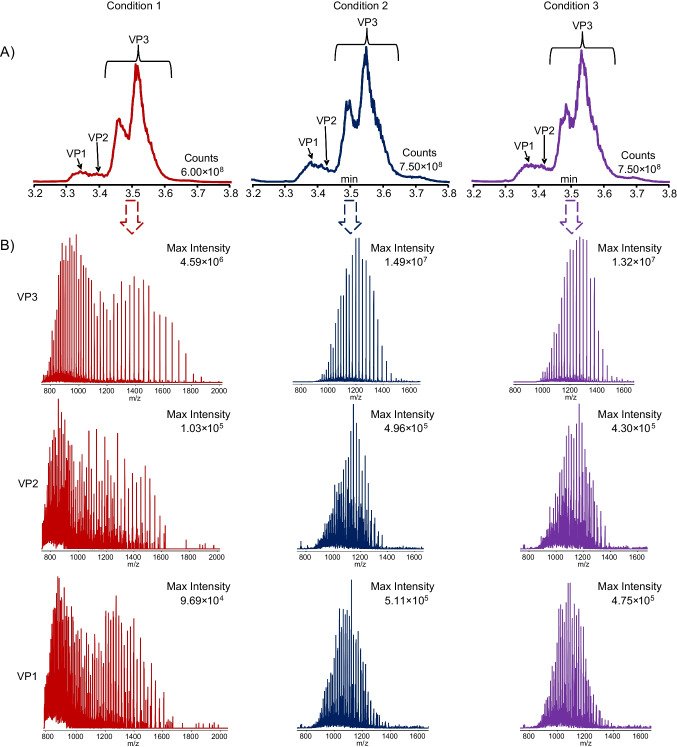
**A** ZipChip CE-MS total ion electropherograms. The maximum intensity value is listed on the right side of each electropherogram in counts. **B** Extracted MS spectra intensity of each VP peak when evaluating the use of DMSO in BGE during sample preparation and analysis. Empty AAV8 capsids were used for this analysis and VP peaks were detected in the electropherograms between 3.2 and 3.8 min. Condition 1 (red): no DMSO is used in the BGE for either sample prep or analysis. Condition 2 (blue): no DMSO is used in the BGE during sample prep, but 4% DMSO is added to the BGE during analysis. Condition 3 (purple): 4% DMSO is added to the BGE for both sample prep and analysis

During initial investigations, it was observed that the MS spectrum of the detected VPs appeared to have a bimodal distribution, suggesting the presence of multiple non-native conformational protein states for each VP (electropherogram of condition 1 in Fig. 1A) [15–17]. It has been shown that small quantities of DMSO in aqueous solution can result in intact proteins becoming more compact and thus generating preference for lower charged protein species [18]. Additionally, the use of DMSO in BGE has previously been demonstrated to improve charge variant analysis when comparing cation exchange chromatography MS (CEX-MS) with ZipChip-based CE-MS [19]. Taking this information into account, it was decided to see whether the addition of a low percentage of DMSO to the Peptides BGE used for analysis could improve MS spectra quality *versus* the use of BGE alone. For this work AAV8E was used, and three analysis conditions were tested: BGE only for both sample incubation and analysis (condition 1); BGE for sample incubation and BGE + 4% (v/v) DMSO for analysis (condition 2); and BGE + 4% (v/v) DMSO for both sample incubation and analysis (condition 3).

Here, the BGE containing 4% DMSO solution was prepared by removing 5 mL of BGE from a new 125 mL bottle of Peptides BGE and then adding 5 mL of LC–MS grade DMSO to the bottle of Peptides BGE [20]. The 5 mL of BGE originally removed from the Peptides BGE bottle was stored in a clean glass vial and subsequently used for sample incubation where appropriate. The presence of the 4% DMSO in the sample analysis improved data quality compared to using Peptides BGE as is without adding DMSO. First, the intensity of the peaks in the electropherograms increased in conditions 2 and 3 compared to condition 1 (Fig. 1A). It was also noted that the MS spectra had a more traditional bell curve charge distribution for each VP in conditions 2 and 3 compared to the bimodal distribution in condition 1 (Fig. 1B). This suggests that the presence of DMSO is reducing the number of conformational protein states for each VP, possibly by causing protein refolding into a more compact protein conformational structure [18, 21]. The presence of fewer, or possibly a single, denatured protein conformational state for each VP directly leads to increased MS signal intensity with the raw signal intensity for each VP increased in conditions 2 and 3 compared to condition 1.

Processing the raw data with BPF 5.1 further reinforced this observation from the raw MS spectra. As exemplified by acetylated-VP3 ((Ac)VP3) and VP2 in Table 1, respectively, the most abundant and least abundant proteoforms detected across all three conditions, it is clearly demonstrated that the presence of DMSO enhanced the summed signal intensity, reduced the total number of charge states, and also reduced the charge state distribution range. In BPF, the summed signal intensity represents the sum of the MS signal intensity values from all the raw data files processed. Here, the data shows that the average sum intensity for each VP proteoform increases in the presence of DMSO. For (Ac)VP3, the average sum intensity increases from 2.34 × 10^10^ in condition 1 to 3.54 × 10^10^ and 3.35 × 10^10^ in conditions 2 and 3, respectively, while for VP2, it increases from 8.91 × 10^7^ in condition 1 to 3.67 × 10^8^ and 3.31 × 10^8^ in conditions 2 and 3, respectively. As previously mentioned, the presence of low percentages of DMSO leads to a preference of lower charged protein species, with the average maximum charge state of (Ac)VP3 dropping from 83.60 in condition 1 to 66.80 and 66.40 in conditions 2 and 3, respectively. A similar observation is seen with VP2 as the average maximum charge state drops from 88.60 in condition 1 to 72.00 and 71.50 in conditions 2 and 3, respectively.

**Table 1:** Impact of DMSO on MS intensity and VP charge states exemplified using acetylated VP3 ((Ac)VP3) and VP2

	(Ac)VP3			VP2		
Condition	1	2	3	1	2	3
Ave. sum intensity*	2.34 × 10^10^	3.54 × 10^10^	3.36 × 10^10^	8.90 × 10^7^	3.67 × 10^8^	3.30 × 10^8^
Ave. # of charge states*	53.20	30.40	30.00	44.80	24.40	24.25
Ave. min. charge state*	31.20	37.40	37.40	44.00	48.60	49.25
Ave. max. charge state*	83.40	66.80	66.40	88.60	72.00	74.75

*This is an average of the data obtained from processing the 5 injections run for each condition in BPF 5.1

Interestingly, we also see that in the presence of DMSO, there is a slight upward shift at the low-end charge states towards higher charged protein species. For (Ac)VP3, the average minimum charge state shifts from 31.20 in condition 1 to 37.40 in both conditions 2 and 3, while for VP2, it shifts from 44.00 in condition 1 to 48.60 and 49.25 in conditions 2 and 3, respectively. These results suggest that the potential protein compaction caused by the presence of DMSO results in a consolidation in the number of overall charges states that predominately, but not entirely, favours lower charged species. Similar trends as those discussed above are seen for all the detected VPs (Table S3). While no additional components were seen, low abundant proteoforms were detected in greater quantities and with better quality scores (a BPF 5.1 metric for determining identification confidence) in conditions 2 and 3 compared to condition 1, with condition 2 generally providing the best results (Table S4). Because of the reasons discussed above, all analysis going forward was performed using the parameters ascribed to condition 2.

### Limit of detection (LoD) evaluation

**Fig. 2:**
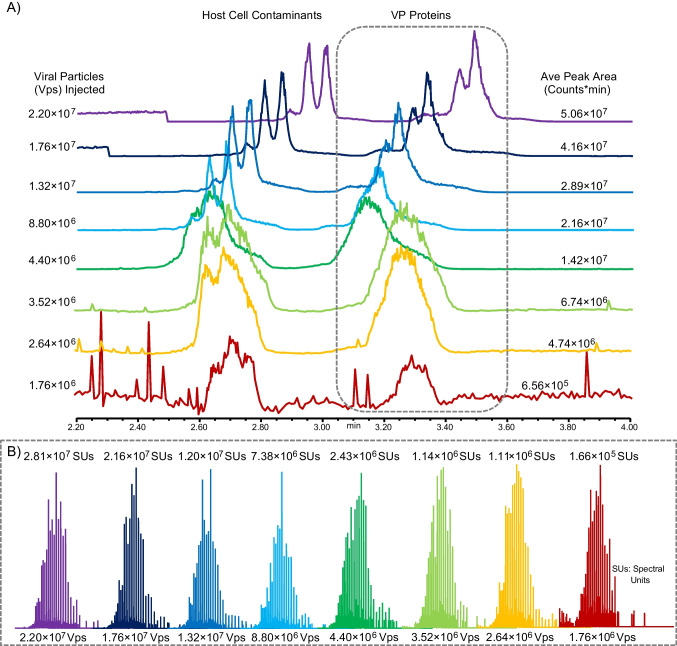
ZipChip CE-MS Limit of Detection testing for AAV capsid analysis. **A** Total ion electropherograms of AAV8E VPs analysed at decreasing concentrations. The peaks representing the VP proteins are encased in the grey box. The other peaks detected are those of host cell contaminants. The VP peak area shown is an average of the total VP peak area of the 5 injections run at each concentration of capsids injected. **B** MS spectra of the total area of the VP peaks in the electropherograms described in (**A**). Spectral intensity value shown is the average spectral intensity of area for the 5 injections run at each concentration of capsids injected. Injection 3 of each sample concentration analysed is used as a representative injection for the electropherograms and the MS spectra shown in (**A**) and (**B**), respectively

Given that production yields for full AAVs are generally low, the high sensitivity of the ZipChip can provide a platform for rapid serotype identity confirmation and VP proteoform identification. Here, a decreasing number of viral particles (Vps) were injected to test the LoD for the ZipChip CE-MS platform, namely 2.20 × 10^7^ Vps, 1.76 × 10^7^ Vps, 1.32 × 10^7^ Vps, 8.80 × 10^6^ Vps, 4.40 × 10^6^ Vps, 3.52 × 10^6^ Vps, 2.64 × 10^6^ Vps, and 1.76 × 10^6^ Vps (see Table S5 for sample dilution details). This roughly corresponds to a mass range of ≈220 pg – ≈26.4 pg of sample being used for LoD evaluation, assuming 1.00 × 10^13^ Vps equals 100 µg. The electropherograms of the analysed samples show two distinct sets of peaks (Fig. 2A).

As discussed in the “Analysis of empty and full capsids from multiple serotypes” section, the set of peaks in the AAV8E samples migrating from ≈3.2–3.8 min corresponds to the VPs, while the set of peaks migrating from ≈2.8–3.2 min are host cell proteins (HCPs) and other cellular contaminants from the density gradient centrifugation process used to purify full AAVs. These additional proteins are not seen during the analysis of full AAVs. As expected, the intensity and total area of the AAV8E VP peaks in the electropherograms decreases as the concentration of Vps injected decreases. The MS signal intensity extracted from the total area of the VP peaks also decreases as the amount of injected Vps decreases (Fig. 2B). Plotting both the electropherographic area of the VP peaks and the extracted MS spectra signal intensity against the amount of Vps injected illustrates their correlation (Supplementary Figure S1 (Figure S1)). Interestingly, for both peak area and MS signal intensity, their relationships are not linear but follow more of a polynomial trend. We saw that the steepest rate of decrease in both MS signal intensity and peak area occurs from an injection of 2.20 × 10^7^ Vps to 1.32 × 10^7^ Vps, before a consistent, near linear rate of decrease occurs from an injection of 1.32 × 10^7^ Vps to 3.52 × 10^6^ Vps and an injection of 1.32 × 10^7^ Vps to 4.40 × 10^6^ Vps for the peak area and signal intensity, respectively. The rate of decrease then begins to slow and level out from an injection of 3.52 × 10^6^ Vps to 1.76 × 10^6^ Vps and an injection of 4.40 × 10^6^ Vps to 1.76 × 10^6^ Vps for the peak area and signal intensity, respectively.

The results obtained from processing the aforementioned CE-MS data within BPF 5.1 demonstrate how lower sample concentrations impact VP proteoform identification (Table 2). At the highest amount of AAV8E injected (2.20 × 10^7^ Vps), 9 separate proteoforms were identified as well was two VP fragments, all of which will be further discussed in the “Analysis of empty and full capsids from multiple serotypes” section: acetylated VP1 ((Ac)VP1), monophosphorylated (Ac)VP1 ((Ac)VP1 + 1× P), diphosphorylated (Ac)VP1 ((Ac)VP1 + 2× P), VP2, monophosphorylated VP2 (VP2 + 1× P), acetylated VP3 ((Ac)VP3), monophosphorylated (Ac)VP3 ((Ac)VP3 + 1× P), un-acetylated VP3, an acetylated VP3 variant (A213(Ac)-VP3), a phosphorylated VP1 fragment, and a VP3 fragment. The component with a mass of 59,843.20 Da (unknown component 1) could not be identified, but as discussed later in the “Analysis of empty and full capsids from multiple serotypes” section, it is thought to be a type of VP3 proteoform. Furthermore, the mass of 59,717.22 Da (unknown component 2) detected when 1.32 × 10^7^ Vps were injected is thought to be the neutral mass loss of a of a carboxyl group (COOH) as discussed in the “Analysis of empty and full capsids from multiple serotypes” section.

**Table 2:** AAV8 VPs detected during LoD testing of the ZipChip CE-MS platform

VPs (theoretical mass)	Matched mass error (ppm)								Relative abundance (%)								Quality score							
Injection amount (× 10^7^ Vps)	2.200	1.760	1.320	0.880	0.440	0.352	0.264	0.176	2.200	1.760	1.320	0.880	0.440	0.352	0.264	0.176	2.200	1.760	1.320	0.880	0.440	0.352	0.264	0.176
(Ac)VP1 + 2 × P (81,826.67 Da)	12.4	9.6	9.1	1.2	-	-	-	-	0.16	0.18^b^	0.07^a^	0.05^a^	-	-	-	-	63.07	71.34	57.37	51.33	-	-	-	-
(Ac)VP1 + 1 × P (81,746.69 Da)	4.4	8.0	3.5	8.4	4.2	1.0	4.6	-	0.93	0.77	0.49^a^	0.46	0.31	0.28	0.22^a^	-	150.92	133.72	122.42	132.41	67.67	60.10	48.44	-
(Ac)VP1 (81,666.71 Da)	7.2	10.4	6.5	12.9	9.6	9.2	-	-	0.57	0.53	0.42	0.33	0.18	0.16^a^	-	-	138.86	131.42	123.42	120.79	74.22	55.33	-	-
VP1 + 1 × P fragment (67,312.85 Da)	11.0	6.2	18.1	-	-	-	-	-	0.13	0.13^a^	0.06^b^	-	-	-	-	-	44.96	49.16	35.84	-	-	-	-	-
VP2 + 1 × P (66,598.08 Da)	3.6	5.7	5.8	3.5	1.0	2.1	6.3	-	1.55	1.27	0.83	0.7	0.63	0.56^a^	0.75^b^	-	116.82	112.90	98.71	97.83	63.17	55.55	65.34	-
VP2 (66,518.10 Da)	7.6	7.7	7.1	7.6	4.9	5.5	4.7	-	0.72	0.61	0.40	0.31	0.21	0.14	0.15	-	103.25	106.52	90.06	86.19	53.70	44.17	52.20	-
(Ac)VP3 + 1 × P (59,884.66 Da)	9.7	12.2	9.3	10.5	6.9	11.8	8.9	21.2	6.35	6.14	6.54	6.30	6.39	5.85	5.85	2.29^b^	88.60	81.67	88.59	77.24	79.34	69.04	69.64	37.00
Unknown component 1 (59,844.17 Da^*^)	u	u	u	u	u	-	-	-	1.43	1.15	0.96	0.89	1.18^b^	-	-	-	46.64	47.64	38.82	35.39	39.11	-	-	-
(Ac)VP3 (59,804.68 Da)	2.4	2.8	1.6	3.4	3.7	2.2	3.6	5.7	100.00	100.00	100.00	100.00	100.00	100.00	100.00	100.00	128.00	127.75	126.21	125.06	128.47	126.24	125.81	110.29
VP3 (59,762.65 Da)	6.5	6.4	4.46	5.7	4.3	5.5	6.5	3.6	51.39	50.76	47.83	47.25	46.42	45.27	42.24	46.10	119.78	115.55	114.14	114.61	111.90	114.04	115.15	93.12
Unknown component 2 (59,717.22 Da^*^)	-	-	u	-	-	-	-	-	-	-	2.48^b^	-	-	-	-	-	-	-	42.38	-	-	-	-	-
VP3 Fragment (59,506.39 Da)	4.0	6.0	1.2	7.4	6.7	6.7	3.7	-	3.71	3.62	3.02	2.92	3.13	2.93	2.28	-	89.47	88.52	76.21	79.51	81.60	75.00	73.94	-
A213(Ac)-VP3 variant (59,191.98 Da)	2.9	4.2	5.1	5.4	3.8	3.3	5.0	2.9	4.75	4.61	4.48	4.46	4.66	3.66	4.09	4.54^b^	85.42	87.28	80.27	78.58	77.86	79.99	73.15	51.98

^a^Only found in 4 of the 5 replicate injections, all other features were found in all 5 injections

^b^Only found in 3 of the 5 replicate injections, all other features were found in all 5 injections

*VPs:* viral proteins, *Vps:* viral particles, *Ac:* N-term acetylation, *P:* phosphorylated, *u:* undetermined as component detected, but not identified; -: component not detected

^*^For unknown components, the mass listed is the average mass from all injections where said component was detected

All identified VP proteoforms were detected in all the injection amounts tested up to and including the concentration of 8.80 × 10^6^ Vps per injection, while at least one proteoform from each VP could be identified in all concentrations up to and including 2.64 × 10^6^ Vps per injection. These findings indicate that complete proteoform identification can be achieved at concentrations as low as 8.80 × 10^6^ Vps per injection, while rapid identity testing can be performed with concentrations as low as 2.64 × 10^6^ Vps per injection. If sample quantity is very low, there is potential that even a concentration of 1.76 × 10^6^ Vps per injection could be used for serotype identity confirmation, as VP3 proteoforms were confidently detected at this concentration. However, such quantities would not be sufficient to differentiate between modified serotypes where modifications occur in the protein sequences unique to VP2 or VP1.

### Analysis of empty and full capsids from multiple serotypes

**Fig. 3:**
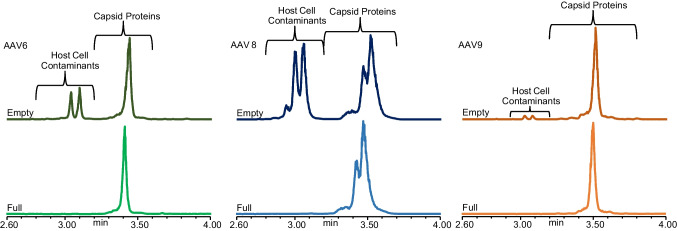
Total ion electropherograms for empty (top) and full (bottom) capsids analysed for serotypes AAV6 (left), AAV8 (middle), and AAV9 (right). Host cell contaminants are detected in empty capsid samples, but not full capsid samples. Injection 3 from the analysis of each sample analysed is used for illustrative purposes

Throughout the optimization process, we have solely worked with empty AAVs, but the ZipChip CE-MS platform is also highly adept at analysing full AAV capsids. Here, we analysed empty and full capsids from the same batch process for serotypes AAV6, AAV8, and AAV9, respectively. Immediately, differences could be observed in the generated data (Fig. 3). As expected, the electropherograms of the full capsids only contain one set of peaks, detected between 3.2 and 3.8 min, corresponding to the presence of the capsid proteins. However, the empty capsids contain an additional second set of peaks detected between 2.8 and 3.2 min. Given that this set of peaks is not seen in the full capsids, it is thought that they correspond to the presence of HCPs or other cellular contaminants in the sample.

According to the supplier, the empty and full capsids were separated using caesium chloride (CsCI) gradient density ultracentrifugation (https://www.virovek.com/aav-system/), which serves to purify the full capsids [22]. It is likely that no consideration was made to separate the empty capsids from other cellular contaminants generated during production, resulting in their presence in the empty samples, but not the full samples. Data processing supports this assumption as all the unknown components detected between 2.8 and 3.2 min in the empty capsids are not seen in the full capsids (Table S6). Furthermore, HCP analysis of the empty and full capsids shows significantly lower levels of HCPs detected in the full samples compared to the empty samples (data not shown), illustrating that many of them were removed from the full capsids during the empty/full separation process. HCPs and other cellular contaminants were not necessarily confined to detection from 2.8 to 3.2 min.

In AAV6, there were two unknown components in the empty capsids that were detected within the peaks containing the VP proteoforms (AAV6 unknown component 1 and AAV6 unknown component 2 in Table S6), but not detected within the full capsids. Their absence in the full capsids suggests that they too were potential HCPs or some other cellular component. While not initially considered when undertaking this work, the ability of the ZipChip to rapidly visualize the presence of potential cellular components suggests that it might be able to be used as a rapid, orthogonal method to monitor the effectiveness of sample purification during downstream processing.

Within the peaks corresponding to the VP proteins, we identified seven proteoforms in AAV6, nine proteoforms in AAV8, and six proteoforms in AAV9 (Table 3). All proteoforms were identified in both the empty and full capsids of their respective serotypes with the exception of a monophosphorylated VP2 proteoform in AAV9 that was only detected in the full capsids. Almost all identifications were made within a 10 ppm mass error, and all were made within a mass error of 20 ppm. As expected, the (Ac)VP1, VP2 and (Ac)VP3 proteoforms were all detected in each AAV serotype analysed. It is well understood that during production, the VP1 and VP3 proteins undergo N-term methionine (Met, M) cleavage at M1 and M203 (M204 for AAV8), respectively, and are then subsequently acetylated (Ac) resulting in the (Ac)VP1 and (Ac)VP3 sequences starting at the M + 1 amino acid [7, 23–25]. For AAV6, AAV8, and AAV9, the M + 1 amino acid is alanine (Ala, A) generating VP1 and VP3 proteoforms starting at A2 and A204 (A205 for AAV8), respectively. These acetylated proteoforms were confirmed through the presence of acetylated peptides starting at A2 and A204 (M205 for AAV8), respectively, detected during peptide mapping (AAV6: Table S7 and Figures S2 and S4, AAV8: Table S8 and Figures S8 and S10, AAV9: Table S9 and Figures S15 and S17). Meanwhile, VP2 for all the serotypes commences at A139 and does not contain any N-term acetylation. This was also confirmed by the presence of peptides starting at A139 with peptide mapping (AAV6: Table S7 and Figure S3, AAV8: Table S8 and Figure S9, AAV9: Table S9 and Figure S16).

**Table 3:** VP proteoforms and fragments identified in the empty and full capsids of AAV6, AAV8, and AAV9

Serotype	Amino acid sequence	Viral Protein	Capsids (empty/full)	Theoretical mass (Da)	Measured mass (Da)	Mass error (ppm)	Average sum intensity	Relative abundance (%)	Fractional abundance (%)	Quality score	Migration time (min)	Theoretical net charge at pH 2.4	Theoretical charge to mass ratio
AAV6	A2(Ac)-L736	(Ac)VP1 + 1xP	Empty Full	81,401.61	81,401.66^a^ 81,401.44^b^	0.6 2.1	1.07 × 10^7^ 3.19 × 10^6^	0.02 0.01	0.01 0.01	37.43^ 40.63	3.308 3.282	+ 79.820	9.81 × 10^−4^
		(Ac)VP1	Empty Full	81,321.63	81,322.85 81,322.15	15.0 6.4	7.80 × 10^7^ 2.30 × 10^7^	0.17 0.10	0.07 0.07	101.10 75.84	3.286 3.274	+ 80.760	9.93 × 10^−4^
	R116-L736	R116-VP1 Fragment	Empty Full	68,650.93	68,651.63 -	10.2 -	2.62 × 10^7^ -	0.06 -	0.02 -	93.19 -	3.273 -	+ 68.104	9.92 × 10^−4^
	A139-L736	VP2 + 1xP	Empty Full	66,174.93	66,175.56 66,175.17	9.6 3.7	3.05 × 10^7^ 4.88 × 10^6^	0.06 0.02	0.03 0.02	47.01 37.56^	3.343 3.316	+ 62.189	9.40 × 10^−4^
		VP2	Empty Full	66,094.95	66,095.48 66,095.17	8.1 3.4	2.38 × 10^8^ 6.98 × 10^7^	0.50 0.32	0.21 0.23	80.02 86.18	3.319 3.306	+ 63.130	9.55 × 10^−4^
	A204(Ac)-L736	(Ac)VP3 + 1xP	Empty Full	59,598.70	59,598.51 59,598.65	3.2 0.8	1.81 × 10^9^ 8.35 × 10^8^	3.84 3.81	1.56 2.71	81.89 74.79	3.460 3.444	+ 54.288	9.11 × 10^−4^
		(Ac)VP3	Empty Full	59,518.72	59,518.83 59,518.88	1.8 2.6	4.71 × 10^10^ 2.19 × 10^10^	100.00 100.00	40.69 71.11	144.18 140.27	3.423 3.405	+ 55.228	9.28 × 10^−4^
	A212(Ac)-L736	(Ac)VP3 Variant	Empty Full	58,890.02	58,890.41 58,890.35	6.7 5.6	6.84 × 10^8^ 2.90 × 10^8^	1.45 1.33	0.59 0.94	75.62 70.58	3.370 3.358	+ 55.228	9.38 × 10^−4^
	A204(Ac)-D590	(Ac)VP3-D590 Fragment	Empty Full	43,221.43	43,221.38^a^ 43,220.68^b^	1.0 17.2	3.28 × 10^7^ 9.60 × 10^6^	0.07 0.04	0.03 0.03	51.84 44.55	3.558 3.551	+ 38.402	8.88 × 10^−4^
AAV8	A2(Ac)-L738	(Ac)VP1 + 2xP	Empty Full	81,826.67	81,827.11^a^ 81,826.07	5.4 7.3	4.55 × 10^7^ 2.06 × 10^7^	0.13 0.08	0.02 0.04	65.05 40.91	3.336 3.311	+ 73.996	9.04 × 10^−4^
		(Ac)VP1 + 1xP	Empty Full	81,746.69	81,746.81 81,746.51	1.4 2.2	2.19 × 10^8^ 1.03 × 10^8^	0.62 0.41	0.10 0.21	141.85 119.09	3.322 3.291	+ 74.937	9.17 × 10^−4^
		(Ac)VP1	Empty Full	81,666.71	81,666.57 81,667.01^a^	1.8 3.7	1.47 × 10^8^ 5.18 × 10^7^	0.42 0.21	0.07 0.10	117.31 97.84	3.310 3.278	+ 75.878	9.29 × 10^−4^
	V132-L738	V132-VP1 + 1xP Fragment	Empty Full	67,312.85	67,312.78^a^ 67,311.84^b^	1.1 15.0	4.05 × 10^7^ 1.63 × 10^7^	0.11 0.07	0.02 0.03	39.11^ 44.46	3.370 3.339	+ 59.268	8.80 × 10^−4^
	A139-L738	VP2 + 1xP	Empty Full	66,598.08	66,597.85 66,597.89	3.5 2.8	4.47 × 10^8^ 2.20 × 10^8^	1.27 0.89	0.21 0.44	114.77 112.75	3.374 3.332	+ 58.286	8.75 × 10^−4^
		VP2	Empty Full	66,518.10	66,517.91 66,518.08	2.8 0.2	2.02 × 10^8^ 9.55 × 10^7^	0.57 0.38	0.09 0.19	100.25 91.06	3.338 3.299	+ 59.226	8.90 × 10^−4^
	A205(Ac)-L738	(Ac)VP3 + 1xP	Empty Full	59,884.66	59,883.74 59,883.62	15.4 17.4	2.34 × 10^9^ 1.70 × 10^9^	6.64 6.85	1.09 3.42	86.65 86.24	3.546 3.511	+ 48.375	8.08 × 10^−4^
		(Ac)VP3	Empty Full	59,804.68	59,804.47 59,804.40	3.6 4.7	3.53 × 10^10^ 2.49 × 10^10^	100.00 100.00	16.35 49.89	130.02 127.81	3.521 3.485	+ 49.316	8.25 × 10^−4^
		VP3	Empty Full	59,762.65	59,761.79 59,761.70	14.3 15.8	1.75 × 10^10^ 1.13 × 10^10^	49.75 45.29	8.13 22.60	120.19 113.27	3.468 3.431	+ 50.316	8.42 × 10^−4^
	G209-L738	G208-VP3 Fragment	Empty Full	59,506.39	59,506.23 59,506.14	2.6 4.1	1.20 × 10^9^ 6.48 × 10^8^	3.40 2.61	0.56 1.30	86.95 75.69	3.441 3.402	+ 50.316	8.46 × 10^−4^
	A213(Ac)-L738	(Ac)VP3 Variant	Empty Full	59,191.98	59,191.85 59,191.87	2.3 1.8	1.57 × 10^9^ 1.23 × 10^9^	4.46 4.94	0.73 2.46	85.68 88.78	3.484 3.450	+ 49.316	8.33 × 10^−4^
AAV9	A2(Ac)-L736	(Ac)VP1	Empty Full	81,290.34	81,291.15 81,290.64	9.9 3.7	5.16 × 10^7^ 6.11 × 10^6^	0.11 0.02	0.08 0.01	88.98 40.04	3.328 3.335	+ 76.860	9.45 × 10^−4^
	R116-L736	R116-VP1 Fragment 1	Empty Full	68,759.79	68,760.41 -	9.1 -	1.06 × 10^8^ -	0.22 -	0.16 -	113.96 -	3.319 -	+ 64.161	9.33 × 10^−4^
	L131-L736	L131-VP1 Fragment 2	Empty Full	67,051.72	67,051.99 67,052.27	4.1 8.2	9.95 × 10^7^ 1.53 × 10^7^	0.20 0.04	0.15 0.03	63.31 37.80^	3.383 3.387	+ 60.170	8.97 × 10^−4^
	A139-L736	VP2 + 1xP	Empty Full	66,289.74	- 66,289.93^a^	- 2.8	- 1.18 × 10^7^	- 0.03	- 0.02	- 45.96	- 3.398	+ 58.247	8.79 × 10^−4^
		VP2	Empty Full	66,209.76	66,210.21 66,210.12	6.7 5.4	3.26 × 10^8^ 1.33 × 10^8^	0.67 0.33	0.49 0.24	83.46 92.82	3.389 3.390	+ 59.188	8.94 × 10^−4^
	F173-L736	F173-VP2 Fragment	Empty Full	62,712.86	62,713.18 62,713.02^a^	5.2 2.7	3.32 × 10^8^ 9.35 × 10^7^	0.68 0.23	0.50 0.17	85.16 76.16	3.597 3.605	+ 52.226	8.33 × 10^−4^
	M203-L736	M203-VP3	Empty Full	59,821.78	59,821.99^a^ 59,821.57^a^	3.5 3.5	2.12 × 10^8^ 1.63 × 10^8^	0.43 0.41	0.32 0.30	70.44 65.96	3.420 3.418	+ 52.286	8.74 × 10^−4^
	A204(Ac)-L736	(Ac)VP3 + 1xP	Empty Full	59,812.60	59,812.87 59,812.85	4.5 4.0	3.75 × 10^9^ 3.95 × 10^9^	7.68 9.79	5.64 7.20	80.83 86.31	3.533 3.531	+ 50.345	8.42 × 10^−4^
		(Ac)VP3	Empty Full	59,732.62	59,732.75 59,732.75	2.1 2.2	4.88 × 10^10^ 4.03 × 10^10^	100.00 100.00	73.50 73.60	121.21 122.11	3.498 3.492	+ 51.286	8.59 × 10^−4^
	A204(Ac)-D657	(Ac)VP3-D657 Fragment	Empty Full	50,469.48	50,469.26 50,469.61	4.4 2.6	4.61 × 10^7^ 4.27 × 10^7^	0.09 0.11	0.07 0.08	43.88 47.97	3.588 3.593	+ 42.363	8.39 × 10^−4^
	A204(Ac)-S538	(Ac)VP3-S538 Fragment	Empty Full	37,557.06	37,557.09 37,557.15	0.6 2.3	1.83 × 10^8^ 1.71 × 10^8^	0.37 0.42	0.25 0.30	48.18 46.90	3.645 3.647	+ 30.547	8.13 × 10^−4^
	A204(Ac)-M518	(Ac)VP3-M518 Fragment	Empty Full	35,387.68	35,387.57 35,387.55	2.9 3.7	6.34 × 10^7^ 6.18 × 10^7^	0.13 0.15	0.09 0.11	42.95 45.40	3.745 3.750	+ 27.594	7.80 × 10^−4^
	A204(Ac)-N512	(Ac)VP3-N512 Fragment	Empty Full	34,728.90	34,728.79 34,728.63	3.2 7.8	4.83 × 10^7^ 3.59 × 10^7^	0.10 0.09	0.07 0.06	45.72 46.05	3.789 3.793	+ 26.589	7.66 × 10^−4^
	A204(Ac)-S448	(Ac)VP3-S448 Fragment	Empty Full	27,803.37	27,802.85 27,802.94	18.5 15.4	2.84 × 10^8^ 7.98 × 10^7^	0.58 0.20	0.39 0.14	47.52 42.72	3.800 3.804	+ 21.594	7.77 × 10^−4^

All identifications were found in all 5 replicate injections performed per condition, a quality score ≥ 40, and a relative abundance ≥ 0.05 except where otherwise noted.

^a^Only found in 4 of the replicate injections, ^b^only found in 3 of the replicate injections, ^quality score below 40, *VP*: viral protein, *Ac*: N-term acetylation, *P*: phosphorylated, -: component not detected.

Surprisingly, a significant amount of unacetylated VP3 (VP3) was also detected during intact analysis of the AAV8 samples, with peptide mapping revealing only 60.87% and 73.64% of VP3 being acetylated in the empty and full capsid samples, respectively (Table S11). Unacetylated VP3 was not detected during the analysis of either the AAV6 or AAV9 serotypes with peptide mapping indicating near 100% acetylation of VP3 in both the empty and full capsids (AAV6: Table S10, AAV9: Table S12). Another unexpected discovery, this time found in the AAV9 samples, was the detection of a low level of unacetylated VP3 that had not undergone N-term methionine cleavage (M203-VP3). Peptide mapping confirmed the presence of this proteoform with the identification of peptides beginning at M203 (Table S9 and FigureS18). N-term acetylation on VP3 is thought to be associated with viral capsid degradation and uncoating, which can influence AAV transduction; it is therefore possible that the presence of unacetylated VP3 proteoforms can impact product efficacy [23, 24]. However, further investigation of this is needed.

In serotypes AAV6 and AAV8, an acetylated VP3 variant commencing at A212 for AAV6 and A213 for AAV8 ((Ac)VP3 Variant) was also identified and confirmed with peptide mapping (AAV6: Table S7 and Figure S5, AAV8: Table S8 and Figure S12). The presence of this variant in some AAV serotypes is the product of their VP3 DNA sequences containing a second ATG initiation codon at M211 (or M212 for AAV8) along with the more common ATG initiation codon at M203 (or M204 for AAV8) [23, 25]. Expression levels of VP3 is thought to be controlled by the Kozak sequence where A in the − 3 position and G in the + 4 position, assuming A in the initiation codon AUG is + 1, is considered to be the optimal and heavily favoured sequence [25, 26]. For AAV6 and AAV8, respectively, the ATG initiation codon at M203 and M204 has this optimal sequence, while the second ATG initiation codon at M211 and M212, respectively, has C in the − 3 position and G in the + 4 position, resulting in the population of (Ac)VP3 being significantly greater than the population of the (Ac)VP3 variant, as observed in this study and others (Table 3) [25]. AAV9 does not contain an M211 amino acid as it does not contain the second ATG initiation codon in its DNA sequence at that position, explaining why no (Ac)VP3 Variant is detected in the AAV9 samples.

Monophosphorylated proteoforms of each VP ((Ac)VP1 + 1×P, VP2 + 1×P and (Ac)VP3 + 1×P) were identified in all serotypes examined, with the exception of (Ac)VP1 of AAV9, where only the unphosphorylated proteoform was seen. Additionally, a diphosphorylated VP1 ((Ac)VP1 + 2×P) proteoform was identified in the AAV8 serotype. The presence of phosphorylation in each serotype was confirmed via peptide mapping. As expected, in both the AAV6 and AAV9 serotypes, the phosphorylated proteoforms for each VP were in low abundance compared to their unphosphorylated counterparts. However, with AAV8, the predominant proteoform for both VP2 and (Ac)VP1 was the phosphorylated proteoform. Peptide mapping of AAV8 revealed high levels of phosphorylation between valine V132 and aspartic acid D185 (V132-D185) of VP1 (Table S11). As VP2 starts at A139, any phosphorylation detected after A139 would be present on both (Ac)VP1 and VP2. A significant amount of phosphorylation was found to be present near threonine T138 in the empty capsids (46.44%), a region unique to VP1. However, the predominant source of phosphorylation found on AAV8 is around serine S153 for the empty capsids (62.70%) and S149 for the full capsids (87.53%), though BPF 5.1 could not determine the exact residue of the phosphorylation in either case. The inability of BPF to determine the exact phosphorylation location might be because phosphorylation has been shown to impact enzymatic digestion efficiency [27], which would explain why the phosphorylated peptides detected within the V131-D184 region are 20–53 amino acids in length. Still, both S149 and S153 are immediately succeeded by a proline (P), which is significant as proteins phosphorylated on serine or threonine immediately preceding proline are known to play essential roles in the regulation of cellular processes [28]. Additional significance to the inability of BPF to successfully determine the exact location of protein phosphorylation within this region is due to the fact that this phosphorylation is detected near the SST motif contained within D155-G159. This is a highly conserved region demonstrated to be essential for AAV transduction efficiency, with phosphomimetic replacements shown to negatively impact virus formation and transduction [29, 30]. Given that phosphorylation on the surface of AAV capsids is suggested to reduce AAV transduction efficiency, phosphorylation on any of the S156, S157, or T158 residues within this motif could be an explanation for this, though further investigation is needed [7, 31–33].

In addition to the full VP proteoforms detected, the ZipChip platform was able to detect a variety of low abundant VP fragments in the serotypes analysed (Table 3). Two types of fragments were identified: those resulting from N-terminus truncation and those resulting from C-terminus truncation. Peptides originating with their new respective N-terminal amino acids or ending with their new respective C-terminal amino acids were detected during peptide mapping to help confirm the presence of these detected fragments (AAV6 Table S7 and Figures S6–S7, AAV8: Table S8 and Figures S13–S14, AAV9: Table S9 and Figures S19–S26). Two fragments were detected in both the AAV6 and AAV8 serotypes: A VP1 fragment (AAV6: R116-L736/AAV8: V132-L738) and a VP3 fragment (AAV6: A204(Ac)-D590/AAV8: G209-L738). Significantly more fragments were identified in the AAV9 serotype with two VP1 fragments (R116-L736 and L131-L736), a VP2 fragment (F173-L736), and five VP3 fragments (A204(Ac)-D657, A204(Ac)-S538, A204(Ac)-M518, A204(Ac)-N512, A204(Ac)-S448).

It is not clear at this time the exact cause of these fragments, but some probable causes are proposed. It has previously been shown that the baculoviral cathepsin (v-cath) protease can cause degradation of VP proteins in AAVs [34]. As the samples used in this study were produced using an Sf9 production system, which requires baculoviral infection for AAV production, it is understandable that this could be a cause for some of the fragments detected. Additionally, the immune response generated by baculoviral infection, which includes the activation of stress response and apoptosis, could contribute to the degradation of capsid proteins or their faulty production [35–37]. This might also mean that the detection of the M203-VP3 protein in AAV9 is reflective of the product being harvested during a late stage of production, where most of the cells would be dying, and thus not functioning as they would when healthy. However, it is impossible to determine this within this study and further investigation is required to do so.

A few of the fragments detected in AAV6 and AAV9 were the result of truncation of the C-terminus of (Ac)VP3. The largest of these fragments is due to the cleavage of the DP peptide at D590 and D657 in AAV6 and AAV9, respectively, through hydrolysis of the aspartic acid, which can occur in acidic conditions [25, 38]. It is possible that these fragments are related to the analysis conditions used as the BGE used during sample preparation and analysis has a pH around 2.4. The smaller (Ac)VP3 fragments in AAV9 are not caused by the hydrolysis of the aspartic acid but might be the result of further degradation at that end once the hydrolysis occurred.

There were also some low abundant unknown components detected that could not be considered HCPs or other cellular contaminants due to their presence in both the empty and full capsids of their respective serotypes. In AAV6, this was AAV6 unknown component 3 (AAV6-UC3, empty: 59,474.15 Da/full: 59,473.23 Da); in AAV8, this was AAV8 unknown component 1 (AAV8-UC1, empty: 59,843.02 Da/full: 59,844.37 Da) and AAV8 unknown component 2 (AAV8-UC2, empty: 59,715.94 Da/full: 59,716.00 Da); and in AAV9, this was AAV9 unknown component 1 (AAV9-UC1, empty: 59,687.55 Da/full: 59,687.18 Da) (Table S6). The exact nature of these components could not be determined, but all are thought to be a type of modified VP3 proteoform. AAV6-UC3, AAV8-UC2, and AAV9-UC1 are considered to be caused by the loss of a carboxyl group (COOH) from (Ac)VP3, unacetylated VP3, and (Ac)VP3, respectively. Most likely a neutral mass loss during CE-MS analysis as there is no difference in migration time between the aforementioned proteoforms and the aforementioned unknown components in their respective serotypes. AAV8-UC2 was initially thought to be a phosphorylated version of the un-acetylated VP3, but as discussed later, the migration time shift associated with phosphorylation was not present to suggest this was the case.

Finally, we explored how PTMs impact proteoform migration and detection times on the ZipChip CE-MS platform as understanding these can aid with proteoform identification when potential components have similar masses. The ZipChip platform separates analytes through differences in electrophoretic mobility, which is a function of an analytes size and charge [39]. Calculating the charge to mass ratios (z/m) of the identified proteoforms will give an indication of their order of detection, as proteins with a larger positive z/m are expected to migrate through the chips channel faster and thus be detected in the MS earlier. ProtPi (https://www.protpi.ch/Calculator/ProteinTool) was utilized to determine the theoretical charge (z) of each identified proteoform at pH 2.4 (the pH of the peptide BGE), and then, this value was used along with their respective theoretical masses (m) to calculate their respective z/m values.

**Fig. 4:**
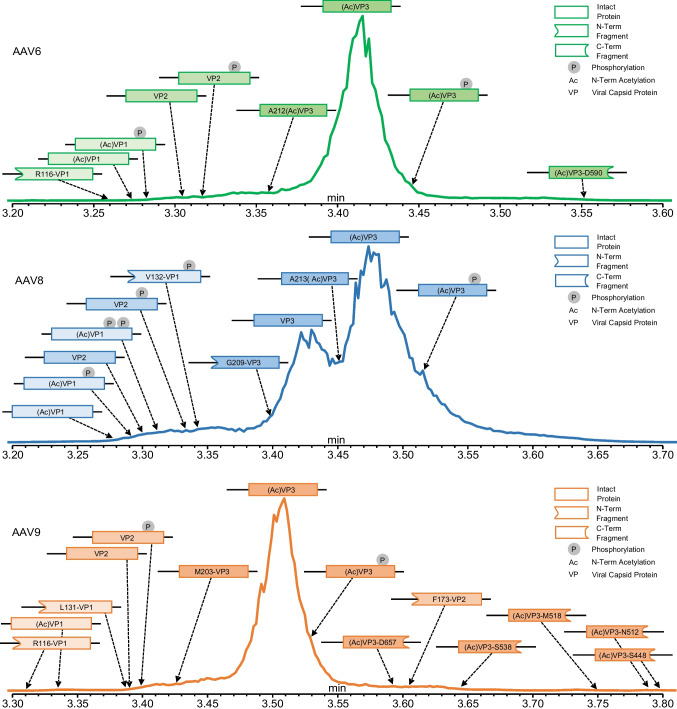
Total ion electropherograms illustrating the VP proteoforms and VP fragments detected in the serotypes analysed. Injection 3 from the analysis of the full capsids for each serotype is used for illustrative purposes: (top) AAV6; (centre) AAV8; (bottom) AAV9

Our initial general observation across all serotypes tested was that VP1 proteoforms were detected first, followed by VP2 proteoforms and VP3 proteoforms (Fig. 4). This was previously seen by Zhang et al. [14] and is expected based off the calculated z/m values of the expected predominant proteoform for each VP ((Ac)VP1, VP2, and (Ac)VP3). When focusing on proteoforms with PTMs, we saw that, across serotypes, the phosphorylated proteoform of a VP was always detected after its unphosphorylated counterpart (Fig. 4). Such observations are a consequence of the negatively charged phosphate reducing the overall charge of the proteoform while also increasing its mass. This leads to lower z/m ratios of the phosphorylated proteoforms compared to their unphosphorylated counterparts (Table 3), resulting in slower migration through the chip and later detection.

Another modification that reduces the overall charge of a protein, and thus influences proteoform detection times, is N-term acetylation. It most likely does so by neutralizing the positively charged N-terminal amino group of the proteoforms. As observed in the AAV8 samples, unacetylated VP3 is detected before (Ac)VP3 (Fig. 4, centre) which correlates with the theoretical z/m calculations of each proteoform (8.42 × 10^−4^ vs. 8.25 × 10^−4^ respectively) (Table 3). Further confirmation of the influence of acetylation on proteoform migration times is observed during analysis of the AAV9 samples when comparing the migration times of the M203-VP3 and (Ac)VP3 (Fig. 4, bottom). If the M203-VP3 was acetylated, it would be expected to migrate slower through the chip than (Ac)VP3. This is because (Ac)VP3 and acetylated M203-VP3 would have the same overall charge (met is an uncharged, non-polar amino acid that does not contribute to the overall charge of the proteoform), but the additional mass of the met amino acid would result in the acetylated M203-VP3 having a smaller z/m ratio than (Ac)VP3 (8.57 × 10^−4^ vs. 8.59 × 10^−4^, respectively). However, in our analysis, M203-VP3 is detected earlier than (Ac)VP3 (E: 3.420 min/F:3.418 min vs. E: 3.498 min/F: 3.492 min, respectively), predominantly due to the lack of acetylation on the M203-VP3 (Fig. 4, bottom).

We also see how the loss of uncharged amino acid residues influences proteoform migration when analysing the retention times of the (Ac)VP3 and (Ac)VP3 variants in serotypes AAV6 and AAV8. In both serotypes, the (Ac)VP3 variants are detected earlier than their respective (Ac)VP3 proteoforms (Fig. 4, top and centre). This is a product of the A204-M211 (or A205-M212 for AAV8) sequence present in (Ac)VP3 but not the (Ac)VP3 variant, not contributing to the overall charge of the (Ac)VP3 proteoform because of the uncharged nature of all the amino acids within said sequence (AAV6: *ASGGGAPM* / AAV8: *AAGGGAPM*). As such, both proteoforms have the same overall charge, but the greater molecular weight of (Ac)VP3 results in a smaller z/m ratio than that of the (Ac)VP3 variant (Table 3).

## Conclusions

In this study, we demonstrate how the microchip ZipChip CE-MS platform can be utilized for rapid in-depth characterization of AAV serotypes, with runs performed in as little as 5 min. We first optimized the platform, demonstrating that low levels of DMSO (4%) improve platform sensitivity and component detection. A LoD study was then performed showing the sensitivity of the ZipChip platform, as all VP proteoforms were detected when as little as 2.64 × 10^6^ viral particles (≈26.4 picograms) were injected. We then compared the analysis of empty and full capsid for serotypes AAV6, AAV8, and AAV9. In doing so we illustrated how the ZipChip platform can determine the presence of HCPs and other cellular contaminants and differentiate them from VP proteoforms.

More importantly, we were able to detect a variety of proteoforms including phosphorylated proteoforms in all serotypes and unacetylated and M203-VP3 proteoforms in AAV8 and AAV9, respectively. We also identified the presence of a VP3 variant at M211 in AAV6 and AAV8, most likely generated by leaky scanning of the initial start codon of VP3 at M203. Additionally, we were able to detect a variety of low abundant fragments originating from the truncation of either the N- or C-terminus. It is possible that the fragments generated from N-terminus truncation are a product of degradation caused by the baculovirus v-cath protease or Sf9 cellular response to baculovirus infection, while fragments generated from C-terminus truncation are the result of forced cleavage at the DP sequence through hydrolysis due to the acidic conditions the analysis was performed under.

Finally, we examined how PTMs influence proteoform migration and detection times to serve as a complementary method to peptide mapping for the confirmation of their presence. Monitoring all of the above is critical as unexpected PTMs or VP modifications can impact product quality and efficacy. The ability of the ZipChip to not only rapidly identify serotypes, but also to detect and monitor PTMs and VP fragments illustrates how it can aid in monitoring product quality during AAV production.

### Supplementary Information

Below is the link to the electronic supplementary material.

Supplementary file1 (PDF 11225 KB)
