# Supplementary material for: Rapid characterization of adeno-associated virus (AAV) capsid proteins using microchip ZipChip CE-MS
Source: Anal Bioanal Chem. 2023 Dec 15;416(4):1069–84. doi: 10.1007/s00216-023-05097-5 (PMC10800304; doi:10.1007/s00216-023-05097-5)

Rapid Characterization of Adeno-Associated Virus (AAV) Capsid Proteins using Microchip ZipChip CE-MS

Josh Smith[1], Sara Carillo[1], Aditya Kulkarni[2], Erin Redman[3], Kate Yu[2], Jonathan Bones[1,4*]

[1]Characterisation and Comparability Laboratory, The National Institute for Bioprocessing Research and Training, Foster Avenue, Mount Merrion, Co. Dublin, Ireland, A94 X099.

[2]908 Devices Inc., 645 Summer Street #201, Boston, MA 02210, United States.

[3]908 Devices Inc., 511 Davis Dr Suite 450, Morrisville, NC 27560, United States.

[4]School of Chemical and Bioprocess Engineering, University College Dublin, Belfield, Dublin 4, Ireland, D04 V1W8.

**Corresponding author*: Jonathan Bones, email: jonathan.bones@nibrt.ie, tel: +353 1215 1800, fax: +353 1215 8116.

TABLE OF CONTENTS

**Supplementary Information 1**	Sample preparation for peptide mapping digestion of AAV capsids
**Supplementary Information 2**	Detailed AAV peptide mapping LC-MS analysis parameters
**Supplementary Information 3**	Detailed AAV peptide mapping data analysis parameters
**Table S1**	BPF 5.1 software parameter settings for intact mass analysis
**Table S2**	BPF 5.1 software parameter settings for peptide mapping analysis
**Table S3**	Effect of sample conditions on MS intensity and AAV8 viral capsid protein (VP) charge state
**Table S4**	Detected AAV8 VPs under investigated sample analysis conditions
**Table S5**	Empty AAV8 capsid dilution series for ZipChip limit of detection (LoD) evaluation
**Table S6**	Unknown components detected in the empty and full capsids of AAV6, AAV8, and AAV9
**Table S7**	Select peptides found in both empty and full AAV6 peptide mapping analysis to confirm presence of VPs and fragments detected during intact mass analysis. Tandem MS (MS/MS) spectra of the highlighted peptides is shown in Figures S2-S7 as illustrative examples of the quality of the peptides identified for each VP or fragment. Peptide information displayed is from the analysis of the full AAV6 capsids
**Table S8**	Select peptides found in both empty and full AAV8 peptide mapping analysis to confirm presence of VPs and fragments detected during intact mass analysis. MS/MS spectra of the highlighted peptides is shown in Figures S8-S14 as illustrative examples of the quality of the peptides identified for each VP or fragment. Peptide information displayed is from the analysis of the full AAV8 capsids
**Table S9**	Select peptides found in both empty and full AAV9 peptide mapping analysis to confirm presence of VPs and fragments detected during intact mass analysis. MS/MS spectra of the highlighted peptides is shown in Figures S15-S26 as illustrative examples of the quality of the peptides identified for each VP or fragment. Peptide information displayed is from the analysis of the full AAV9 capsids
**Table S10**	Acetylation and phosphorylation PTMs identified during peptide mapping of AAV6 empty and full capsids with a relative abundance ≥ 1%. ~ indicates that the exact location of the modification could not be determined by BPF 5.1, but the modification is near or on the listed residue
**Table S11**	Acetylation and phosphorylation PTMs identified during peptide mapping of AAV8 empty and full capsids with a relative abundance ≥ 1%. ~ indicates that the exact location of the modification could not be determined by BPF 5.1, but the modification is near or on the listed residue
**Table S12**	Acetylation and phosphorylation PTMs identified during peptide mapping of AAV9 empty and full capsids with a relative abundance ≥ 1%. ~ indicates that the exact location of the modification could not be determined by BPF 5.1, but the modification is near or on the listed residue
**Figure S1**	Plot of total ion electropherogram area (blue line on top) and extracted apex MS signal intensity (orange line on bottom) versus Vps injected during LoD study
**Figure S2**	Tandem MS (MS/MS) spectra of the *AADGYLPDWLEDNLSEGIRE* (A2-E21) peptide, with acetylation at A2, from peptide mapping of full AAV6 capsids, as identified using BPF 5.1. Peptide is an example of the detected peptides that indicate the presence of the (Ac)VP1 capsid protein identified during intact mass analysis (Table S7)

**Figure S3**	MS/MS spectra of the *APGKKRPVEQSPQEPDSSSGIGKTGQQPAKKRLNFGQTGD* (A139-D178) peptide from peptide mapping of full AAV6 capsids, as identified using BPF 5.1. Peptide is an example of the detected peptides that indicate the presence of the VP2 capsid protein identified during intact mass analysis (Table S7)
**Figure S4**	MS/MS spectra of the *ASGGGAPMADNNEGADGVGNASGNWHCDSTWLGDRVITT* (A204-T242) peptide, with acetylation at A204, from peptide mapping of full AAV6 capsids, as identified using BPF 5.1. Peptide is an example of the detected peptides that indicate the presence of the VP3 capsid protein identified during intact mass analysis (Table S7)
**Figure S5**	MS/MS spectra of the *ADNNEGADGVGNASGNWHCDSTWLGDRVITTSTRT* (A212-T246) peptide, with acetylation at A212, from peptide mapping of full AAV6 capsids, as identified using BPF 5.1. Peptide is an example of the detected peptides that indicate the presence of the (Ac)VP3 Variant capsid protein identified during intact mass analysis (Table S7)
**Figure S6**	MS/MS spectra of the *RAVFQAKKRVLEPFGL* (R116-L131) peptide from peptide mapping of full AAV6 capsids, as identified using BPF 5.1. Peptide is an example of the detected peptides that indicate the presence of the R116-VP1 fragment identified during intact mass analysis (Table S7)
**Figure S7**	MS/MS spectra of the *EEIKATNPVATERFGTVAVNLQSSSTD* (E564-D590) peptide from peptide mapping of full AAV6 capsids, as identified using BPF 5.1. Peptide is an example of the detected peptides that indicate the presence of the (Ac)VP3-D590 fragment identified during intact mass analysis (Table S7)
**Figure S8**	MS/MS spectra of the *AADGYLPDWLEDNLSEGIRE* (A2-E21) peptide, with acetylation at A2, from peptide mapping of full AAV8 capsids, as identified using BPF 5.1. Peptide is an example of the detected peptides that indicate the presence of the (Ac)VP1 capsid protein identified during intact mass analysis (Table S8)
**Figure S9**	MS/MS spectra of the *APGKKRPVEPSPQRSPDSSTGIGKKGQQPARKRLNFGQTGDSESVPD* (A139-D185) peptide, with phosphorylation around S149 (exact location could not be determined), from peptide mapping of full AAV8 capsids, as identified using BPF 5.1. Peptide is an example of the detected peptides that indicate the presence of the VP2 capsid protein identified during intact mass analysis (Table S8). The relatively poor spectrum is a result of the phosphorylation present which impacts digestion efficiency of the pepsin, resulting in the large peptide identified
**Figure S10**	MS/MS spectra of the *AAGGGAPMADNNEGADGVGSSSGNWHCDSTWLGDRVITT* (A205-T243) peptide, with acetylation at A205, from peptide mapping of full AAV8 capsids, as identified using BPF 5.1. Peptide is an example of the detected peptides that indicate the presence of the (Ac)VP3 capsid protein identified during intact mass analysis (Table S8)
**Figure S11**	MS/MS spectra of the *AAGGGAPMADNNEGADGVGSSSGNWHCDSTWLGDRVIT* (A205-T242) peptide, with no acetylation, from peptide mapping of full AAV8 capsids, as identified using BPF 5.1. Peptide is an example of the detected peptides that indicate the presence of the VP3 capsid protein without N-term acetylation identified during intact mass analysis (Table S8)
**Figure S12**	MS/MS spectra of the *ADNNEGADGVGSSSGNWHCDSTWLGDRVITTSTRT* (A213-T247) peptide, with acetylation at A213, from peptide mapping of full AAV8 capsids, as identified using BPF 5.1. Peptide is an example of the detected peptides that indicate the presence of the (Ac)VP3 Variant capsid protein identified during intact mass analysis (Table S8)

**Figure S13**	MS/MS spectra of the *VEEGAKTAPGKKRPVEPSPQRSPDSSTGIGKKGQQPARKRLNFGQTGDSESVPD* (V132-D185) peptide, with phosphorylation around S149 (exact location could not be determined), from peptide mapping of full AAV8 capsids, as identified using BPF 5.1. Peptide is an example of the detected peptides that indicate the presence of the V132-VP1 fragment identified during intact mass analysis (Table S8). The relatively poor spectrum is a result of the phosphorylation present which impacts digestion efficiency of the pepsin, resulting in the large peptide identified
**Figure S14**	MS/MS spectra of the *GAPMADNNEGADGVGSSSGNWHCDSTWLGDRVIT* (G209-T242) peptide from peptide mapping of full AAV8 capsids, as identified using BPF 5.1. Peptide is an example of the detected peptides that indicate the presence of the G209-VP3 fragment identified during intact mass analysis (Table S8)
**Figure S15**	MS/MS spectra of the *AADGYLPDWLEDNLSEGIRE* (A2-E21) peptide, with acetylation at A2, from peptide mapping of full AAV9 capsids, as identified using BPF 5.1. Peptide is an example of the detected peptides that indicate the presence of the (Ac)VP1 capsid protein identified during intact mass analysis (Table S9)
**Figure S16**	MS/MS spectra of the *APGKKRPVEQSPQEPDSSAGIGKSGAQPAKKRLNFGQTGDTESVPDPQPIGEPPAAPSGVGSLTM* (A139-M203) peptide, from peptide mapping of full AAV9 capsids, as identified using BPF 5.1. Peptide is an example of the detected peptides that indicate the presence of the VP2 capsid protein identified during intact mass analysis (Table S9)
**Figure S17**	MS/MS spectra of the *ASGGGAPVADNNEGADGVGSSSGNWHCDSQWLGDRVIT* (A204-T241) peptide, with acetylation at A204, from peptide mapping of full AAV9 capsids, as identified using BPF 5.1. Peptide is an example of the detected peptides that indicate the presence of the (Ac)VP3 capsid protein identified during intact mass analysis (Table S9)
**Figure S18**	MS/MS spectra of the *MASGGGAPVADNNEGADGVGSSSGNWHCDSQWLGDRVITTSTRT* (M203-T246) peptide from peptide mapping of full AAV9 capsids, as identified using BPF 5.1. Peptide is an example of the detected peptides that indicate the presence of the M203-VP3 capsid protein identified during intact mass analysis (Table S9). This is a VP3 protein that didn't undergo expected N-term methionine cleavage and subsequent N-term acetylation
**Figure S19**	MS/MS spectra of the *RAVFQAKKRLLEPLGL* (R116-L131) peptide from peptide mapping of full AAV9 capsids, as identified using BPF 5.1. Peptide is an example of the detected peptides that indicate the presence of the R116-VP1 fragment identified during intact mass analysis (Table S9)
**Figure S20**	MS/MS spectra of the *LVEEAAKTAPGKKRPVE* (L131-E147) peptide from peptide mapping of full AAV9 capsids, as identified using BPF 5.1. Peptide is an example of the detected peptides that indicate the presence of the L131-VP1 fragment identified during intact mass analysis (Table S9)
**Figure S21**	MS/MS spectra of the *FGQTGDTESVPDPQPIGEPPAAPSGVGSL* (F173-L201) peptide from peptide mapping of full AAV9 capsids, as identified using BPF 5.1. Peptide is an example of the detected peptides that indicate the presence of the F173-VP2 fragment identified during intact mass analysis (Table S9)
**Figure S22**	MS/MS spectra of the *IKNTPVPAD* (I649-D657) peptide from peptide mapping of full AAV9 capsids, as identified using BPF 5.1. Peptide is an example of the detected peptides that indicate the presence of the (Ac)VP3-D657 fragment identified during intact mass analysis (Table S9)

**Figure S23**	MS/MS spectra of the *ALNGRNSLMNPGPAMASHKEGEDRFFPLS* (A510-S538) peptide from peptide mapping of full AAV9 capsids, as identified using BPF 5.1. Peptide is an example of the detected peptides that indicate the presence of the (Ac)VP3-S538 fragment identified during intact mass analysis (Table S9)
**Figure S24**	MS/MS spectra of the *FAWPGASSWALNGRNSLM* (F501-M518) peptide from peptide mapping of full AAV9 capsids, as identified using BPF 5.1. Peptide is an example of the detected peptides that indicate the presence of the (Ac)VP3-M518 fragment identified during intact mass analysis (Table S9)
**Figure S25**	MS/MS spectra of the *AVQGRNYIPGPSYRQQRVSTTVTQNNNSEFAWPGASSWALN* (A472-N512) peptide from peptide mapping of full AAV9 capsids, as identified using BPF 5.1. Peptide is an example of the detected peptides that indicate the presence of the (Ac)VP3-N512 fragment identified during intact mass analysis (Table S9)
**Figure S26**	MS/MS spectra of the *YLYYLS* (Y443-S448) peptide from peptide mapping of full AAV9 capsids, as identified using BPF 5.1. Peptide is an example of the detected peptides that indicate the presence of the (Ac)VP3-S448 fragment identified during intact mass analysis (Table S9)

Supplementary Information 1: Sample preparation for peptide mapping digestion of AAV capsids

Sample preparation for AAV peptide mapping digestion was performed as follows. For each digestion, 4 µg of AAV sample was brought to a total volume of 50 µL with LC-MS grade water in a 500 µL Eppendorf protein LoBind tube (Eppendorf, Dublin). 148 µL of SMART Digest buffer and 2 µL of 0.5 M TCEP (5 mM final concentration) were then added for reduction and denaturation. Next 15 µL of pepsin beads were combined with 100 µL SMART Digest buffer and a digest wash solution composed of 50 µL of SMART Digest buffer and 150 µL of LC-MS grade water was prepared. The denatured AAV sample, the pepsin bead mixture, and the wash solution were then added to different wells of a 96-deepwell plate (Thermo Scientific, Vantaa, Finland). Sample digestion on the pepsin beads was performed using a Thermo Scientific KingFisher Duo Prime purification system under the control of Thermo Scientific BindIt software, version 4.0. Samples AAV6 Empty (AAV6E), AAV6 Full (AAV6F), AAV8 Empty (AAV8E), AAV9 Empty (AAV9E), and AAV9 Full (AAV9F) were incubated for 30 minutes while AAV8 Full (AAV8F) was incubated for 45 minutes. All incubations were performed at 70°C, set to medium mixing speed. After incubation, the beads were removed, and remaining sample volume was transferred to 1.5 mL Eppendorf protein LoBind tubes (Eppendorf, Dublin). Samples were centrifuged at 14,000 ⍰ g for 5 min to pellet any pepsin beads that had not been removed. The remaining supernatant was then transferred to a vial for liquid chromatography-mass spectrometry (LC-MS) for analysis.

Supplementary Information 2: Detailed AAV peptide mapping LC-MS analysis parameters

Peptide mapping LC-MS analysis was performed in technical triplicate using a Vanquish Neo UHPLC coupled to an Orbitrap Exploris 480 (Thermo Scientific, Bremen, Germany) following a modified version of the procedure described in Guapo *et al* (9). Mobile phase A was UHPLC-MS grade water containing 0.1 % (v/v) FA and mobile phase B was Optima™ LC-MS grade ACN containing 0.1 % (v/v) FA. Per injection, 250ng of digested AAV sample from Section 2.3 (assumed 100% digestion) was separated using a Thermo Scientific Easy-Spray™ PepMap™ Neo C18 column, 2 µm, 75 µm × 50 cm (Thermo Scientific, Sunnyvale, CA, USA). After injection, a hold at 2% B was performed for 10 min before peptide separation was performed using a linear gradient (Curve 5) from 2% B to 25% B over 60 min, followed by an increase to 45% B over 30 min. A column wash was then performed over 10 min by performing a step gradient to 80% B followed by an isocratic hold for 5 min. A step gradient to 2% B followed by a hold for 2 min was performed before a step gradient back to 80% B with a subsequent hold for 3 min was set to complete the wash. The flow rate was kept constant at 250 nL/min and the column temperature was maintained at 45°C.

Data-dependent acquisition (DDA)-MS analysis was performed in positive ion mode. Full scans were acquired at a resolution of 60,000 between a mass range of *m/z* 200–2,000. The RF Lens was set to 40% while the AGC target was set to 1.0×10^6 with a maximum injection time of 50ms and 1 microscan. MS-MS (MS^2) fragment scans were acquired using a resolution setting of 15,000 with an AGC target of 4.5×10^4, a maximum injection time of 70 ms, an isolation window of m/z 1.2, and a signal intensity threshold of 5.0×10^3. Fragmentation of the twenty most abundant precursor ions was performed using a normalized collision energy set to 28 with a dynamic exclusion set for 45s. Charge states from 2-7 were included for detection. The MS tune parameters were as follows: Spray voltage was set to 1.7 kV; Capillary temperature was set to 300°C.

Supplementary Information 3: Detailed AAV peptide mapping data analysis parameters

Peptide identification and relative post translational modification (PTM) quantitation was performed using a modified version of what is outlined by Guapo et al (9). Briefly, a peptide mapping analysis experiment was created in BioPharma Finder™ (BPF) Version 5.1 (Thermo Scientific, San Jose, CA, USA) to process the raw data files generated from Supplementary Information 1 using the parameters summarized in Table S2. Only peptides with a ≥ 95% confidence score, ≥ 1 x 10^5 average MS area, and within ± 5 ppm mass accuracy were included for sequence coverage evaluation. Peptides with adducts, unknown modifications, gas phase ions, and non-specific generated peptides fitting these parameters were filtered from the results and thus not utilized for sequence coverage determination.

For PTM quantitation the same parameters as for peptide sequence coverage were applied. PTMs were identified automatically in BPF by their mass differences compared to unmodified peptides generated from pepsin digestion. Relative PTM abundance was expressed in BPF as a percentage of PTM presence on a peptide to all forms of said peptide present. Manual validation of peptides selected for relative PTM quantitation was performed in addition to automatic software assignment to ensure accurate quantitation.

Table S1: BPF 5.1 software parameter settings for intact mass analysis

**Component Detection Parameters**	
Chromatogram Parameters	
Use Restricted Time	Enabled
Time Limits (Minutes)	2.6 to 4.0
m/z Range	740 to 2,000
Chromatogram Trace Type	TIC
Sensitivity	High
Rel. Intensity Threshold (%)	1
Source Spectra Method	Sliding Windows
Sliding Windows Definition	
RT Range	2.6 to 4.0
Target Avg Spectrum Width	0.1
Target Avg Spectrum Offset Scan	1
Sliding Windows Merging Parameters	
Merge Tolerance	30 ppm
Merge Scheme	Improved Merge Scheme
Max RT Gap	0.1 minutes
Min. Number of Detected Intervals	3
Deconvolution Algorithm	ReSpect™ (Isotopically Unresolved)
ReSpect Main Parameters	
Deconvolution Results Filter	
Output Mass Range	25,000 to 85,000
Deconvoluted Spectra Display Mode	Isotopic Profile (new)
Charge State Distribution	
Deconvolution Mass Tolerance	10 ppm
Choice of Peak Model	
Choice of Peak Model	Intact Protein
Resolution at 400 m/z	
Raw File Specific	
ReSpect Advanced Parameters	
Charge State Distribution	
Model Mass Range	10,000 to 1,000,000
Charge State Range	5 to 100
Minimum Adjacent Charges (low & high model mass)	4 to 6
Noise Parameters	
Rel. Abundance Threshold (%)	0
Deconvolution Quality	
Quality Score Threshold	0
Choice of Peak Model	
Target Mass	71,000 Da

Peak Model Parameters	
Number of Peak Models	1
Left/Right Peak Shape	Left 2 Right 2
Peak Filter Parameters	
Peak Detection Minimum Significance Measure	1
Peak Detection Quality Measure	95%
Specialized Parameters	
Peak Model Width Factor	1
Intensity Threshold Scale	0.01
Deconvolution Parameters	
Noise Compensation	Enabled
Charge Carrier	H+ (1.00727663)
Negative Charge	Disabled
**Identification Parameters**	
Sequence Matching Mass Tolerance	50 ppm
Enable Drug-to-Antibody Ratio (Protein Only)	Disabled
Variable Modification Selected	
Multiconsensus Component Merge	
Mass Tolerance	10 ppm
RT Tolerance	0.1 minutes
Minimum Number of Required Occurrences	1
**Sequences, Modifications**	
Sequence Name	AAV6_VP1 or AAV8_VP1 or AAV9_VP1
Static Modifications	Acetylation (N-term)(NTerm,A,x1)
Sequence Name	AAV6_VP2 or AAV8_VP2 or AAV9_VP2
Static Modifications	None
Sequence Name	AAV6_VP3 or AAV8_VP3 or AAV9_VP3
Static Modifications	Acetylation (N-term)(NTerm,A,x1)
Sequence Name	AAV8_VP3_No N Term Acetylation
Static Modifications	None
Sequence Name	AAV9_VP3 with Methionine
Static Modifications	None
Sequence Name	AAV6 VP3 Variant or AAV8 VP3 Variant
Static Modifications	Acetylation (N-term)(NTerm,A,x1)
Sequence Name	AAV6_R116-VP1 Fragment, AAV8_V132-VP1 Fragment, AAV8_G208-VP3 Fragment, AAV9_R116-VP1 Fragment, AAV9_L131-VP1 Fragment, AAV9_F173-VP2 Fragment
Static Modifications	None
Sequence Name	AAV6_VP3_A204toD590_Fragment, AAV9_VP3_A204toD657_Fragment, AAV9_VP3_A204toS538_Fragment, AAV9_VP3_A204toM518_Fragment, AAV9_VP3_A204toN512_Fragment, AAV9_VP3_A204toS448_Fragment
Static Modifications	Acetylation (N-term)(NTerm,A,x1)
Variable Modifications	Acetylation (SideChain), Deamidation (N), Oxidation (M,W), Double Oxidation (M,W,C), Triple Oxidation (M,W,C), Oxidation to kynurenine (W), Phosphorylation1x_Oxidation1x (S,T,Y,M,W,C), Phosphorylation (S,T,Y), Double Phosphorylation (S,T,Y), Triple Phosphorylation (S,T,Y), Double Deamidation (N.Q), Succinimide (D), Succinimide (N), NH3 loss_Glutarimide (Q)
Max # Modifications	3
Glycosylation	None

Table S2: BPF 5.1 software parameter settings for peptide mapping analysis

**Component Detection Parameters**	
Absolute MS Signal Threshold (MS Noise Level * S/N Threshold)	250,000
MS Noise Level	1,000
S/N Threshold	250
Beginning Peak Width (min)	0.1
Typical Chromatographic Peak Width (min)	0.3
Ending Peak Width (min)	0.75
Maximum Chromatographic Peak Width (min)	0.5
Use Restricted Time	Yes
Time Limits (Minutes)	5 to 115
Relative MS Signal Threshold (% of base peak)	1
Relative Analog Threshold (% of highest peak)	1
Width of Gaussian Filter (represented as 1/n of chromatographic peak width)	3
Minimum Valley to be Considered as Two Chromatographic Peaks (%)	80.00%
Minimum MS Peak Width (Da)	1.2
Maximum MS Peak Width (Da)	4.2
Mass Tolerance (ppm for high-res or Da for low-res)	4
Maximum Retention Time Shift (min)	1
Maximum Mass (Da)	30,000
Mass Centroiding Cutoff (% from base)	15
**Identification Parameters**	
Search by Full MS Only	No
Use MS/MS	Use All MS/MS
Maximum Peptide Mass	7,000
Mass Accuracy (ppm)	5
Minimum Confidence	0.8
Maximum Number of Modifications for a Peptide	1
Mass Changes for Unspecified Modifications	-129 to 169
Glycosylation	None
Search for Amino Acid Substitutions	None
Enable Residue Deletion	False
Perform Disulfide Bond Search	No
Protease	Pepsin
N-Term	FIMYWV
C-Term	CDEFLMTWY
Specificity	High
Static Modifications	None
Variable Modifications	Acetylation (N-term), Acetylation (SideChain), Deamidation (N), Deamidation (Q), Dimethylation (K), Glycation (K), Oxidation (C), Oxidation (M,W), Phosphorylation (S,T,Y), Phopho S (S,T,Y), SUMOylation by SUMO1 (K), SUMOylation by SUMO2 (K), Ubiquitination (K), Amidation (D,E), Methylation (K,R), Formylation (S,T), Double Oxidation (M,W,C), Oxidation to hydroxy-kynurenine (W), Oxidation to kynurenine (W), Double Phosphorylation (S,T,Y), Triple Phosphorylation (S,T,Y), Phosphorylation1x_Oxidation1x (S,T,Y,M,W,C), Cysteaminylation (C), Double Deamidation (N.Q, Succinimide (N), Succinimide (D), NH3 loss_Glutarimide (Q), Hex3HexNAc2dHex1(SideChain), Hex4HexNAc4dHex3(N), Hex4HexNAc2dHex1 (N), Hex3HexNAc3dHex2 (N), Hex3HexNAc5dHex3 (N), Hex2HexNAc4dHex4 (N), Hex2HexNAc6dHex1 (N), Hex4HexNAc4dHex2 (N), Hex5HexNAc2dHex5 (N), Hex5HexNAc5dHex1 (N), Hex6HexNAc5dHex1 (N), Hex2HexNAc2 (N), Hex2HexNAc2dHex1 (N), Hex2HexNAc2dHex2 (N), Hex4HexNAc2dHex2 (N), Hex1HexNAc2dHex1 (N), Hex1HexNAc2dHex2 (N), HexNAc1 (N), HexNAc1dHex1 (N), Man3 (N), Man4 (N), Man5 (N), Man6 (N), Man7 (N), Man8 (N), Man9 (N), Hex3HexNAc2dHex2 (N), Hex3HexNAc4dHex4 (N), Hex3HexNAc4dHex2 (N), Hex3HexNAc4dHex3 (N), Hex3HexNAc3dHex1 (N), Hex3HexNAc4dHex1 (N)

Table S3: Effect of sample conditions on MS intensity and AAV8 viral capsid protein (VP) charge state

Capsid Viral Proteins	Ave. Sum Intensity*			Ave. # of Charge States*			Ave. Min. Charge State*			Ave. Max. Charge State*		
**Condition**	1	2	3	1	2	3	1	2	3	1	2	3
**(Ac)VP1 + 2x P**	2.09×10^{7}	2.19×10^{8}	-	28.33	27.00	-	63.33	64.00	-	100.00	90.00	-
**(Ac)VP1 + 1x P**	2.06×10^{8}	7.98×10^{8}	7.49×10^{8}	49.80	34.80	33.60	51.20	58.80	61.00	100.00	92.80	94.60
**(Ac)VP1**	1.56×10^{8}	4.37×10^{8}	5.16×10^{8}	55.00	36.20	33.80	50.60	58.80	59.80	104.6	94.40	92.80
**VP2 + 1x P**	1.85×10^{8}	7.19×10^{8}	7.14×10^{8}	48.80	26.80	25.20	41.40	48.60	49.20	89.20	76.00	73.40
**VP2**	8.90×10^{7}	3.67×10^{8}	3.30×10^{8}	44.80	24.40	24.25	44.00	48.60	49.25	88.60	72.00	74.75
**(Ac)VP3 + 1x P**	5.33×10^{8}	2.27×10^{9}	2.15×10^{9}	43.00	26.20	26.40	35.80	40.80	40.80	77.8	66.00	66.20
**(Ac)VP3**	2.34×10^{10}	3.54×10^{10}	3.36×10^{10}	53.20	30.40	30.00	31.20	37.40	37.40	83.40	66.80	66.40
**VP3**	1.03×10^{10}	1.86×10^{10}	1.80×10^{10}	48.40	28.60	28.00	32.60	40.00	40.80	80.00	68.60	67.20
**VP3 Fragment**	3.12×10^{8}	1.22×10^{9}	1.07×10^{9}	41.40	21.20	22.20	36.60	43.20	43.20	77.20	63.40	64.80
**A213(Ac)-VP3 Variant**	3.15×10^{8}	1.48×10^{9}	1.43×10^{9}	35.25	22.40	20.20	39.50	42.20	42.80	75.25	63.60	62.00

*This is an average of the data obtained from processing the 5 injections run for each condition in BPF 5.1.
VP = viral protein, Ac = N-Term Acetylation, P = Phosphorylated, - = Not identified under that condition

Table S4: Identified AAV8 viral capsid proteins (VPs) under investigated sample analysis conditions

Capsid Viral Proteins (Theoretical mass)	Matched Mass Error (ppm)			Relative Abundance (%)			Fractional Abundance (%)			Quality Score		
**Condition**	1	2	3	1	2	3	1	2	3	1	2	3
**(Ac)VP1 + 2x P (81,826.67 Da)**	6.7[b]	0.7[a]	-	0.06	0.56	-	0.01	0.07	-	34.33[d]	74.78	-
**(Ac)VP1 + 1x P (81,746.69 Da)**	1.7	0.0	2.1	0.57	2.05	1.83	0.09	0.24	0.21	149.73	127.34	115.08
**(Ac)VP1 (81,666.71 Da)**	6.4	1.6	1.0	0.43	1.12	1.26	0.07	0.13	0.14	168.28	94.43	117.70
**VP2 + 1x P (66,598.08 Da)**	2.7	1.2	8.3	0.51	1.85	1.74	0.08	0.22	0.20	107.14	91.33	92.65
**VP2 (66,518.10 Da)**	6.0	3.4	3.1[a]	0.25	0.94	0.81	0.04	0.11	0.09	81.58	98.99	83.38
**(Ac)VP3 + 1x P (59,884.66 Da)**	1.5	15.3	14.3	1.24	5.81	5.25	0.19	0.68	0.60	60.41	75.34	77.02
**(Ac)VP3 (59,804.68 Da)**	3.9	5.8	5.9	65.15[c]	90.87[c]	81.87[c]	10.11	10.64	9.39	262.41	122.59	119.71
**VP3 (59,762.65 Da)**	9.5	14.9	13.5	28.58	47.77	43.82	4.43	5.60	5.03	238.27	114.04	114.50
**VP3 Fragment (59,506.81 Da)**	1.6	2.5	5.0	0.87	3.13	2.61	0.13	0.37	0.30	79.64	85.42	81.09
**A213(Ac)-VP3 Variant (59,191.98 Da)**	3.3[a]	1.7	5.1	0.87	3.79	3.48	0.14	0.44	0.40	55.24	75.60	83.71

[a] Only found in 4 of the 5 replicate injections, all other features found in all 5 injections unless otherwise stated
[b] Only found in 3 of the 5 replicate injections, all other features found in all 5 injections unless otherwise stated
[c] (Ac)VP3 is the most abundant VP. The most abundant feature is an unknown component detected in the host cell contaminants peaks
[d] Quality Score below 35
VP = viral protein, Ac = N-Term Acetylation, P = Phosphorylated, - = Not identified under that condition

Table S5: Empty AAV8 capsid dilution series for ZipChip limit of detection (LoD) evaluation

Initial AAV Concentration (Viral particles (Vps)/uL)	*Initial AAV Volume (uL)*	*Total Incubation Volume (uL)*	*Sample Concentration after Incubation (Vps/uL)*	*Injection Volume (nL)*	*Final Injection amount (Vps)*
2.00×10^{10}	5	25	**4.00×10^{9}**	5.5	**2.20×10^{7}**
	4	25	**3.20×10^{9}**	5.5	**1.76×10^{7}**
	3	25	**2.40×10^{9}**	5.5	**1.32×10^{7}**
	2	25	**1.60×10^{9}**	5.5	**8.80×10^{6}**
4.00×10^{9}*	5	25	**8.00×10^{8}**	5.5	**4.40×10^{6}**
	4	25	**6.40×10^{8}**	5.5	**3.52×10^{6}**
	3	25	**4.80×10^{8}**	5.5	**2.64×10^{6}**
	2	25	**3.20×10^{8}**	5.5	**1.76×10^{6}**

*2E10 Vp/uL stock solution diluted 1 in 5 with BGE to generate 4E9 Vps/uL sample

Table S1: Unknown components detected in the empty and full capsids of AAV6, AAV8, and AAV9

Serotype	Capsid Sample	Component	Measured Mass (Da)	Average Sum Intensity	Relative Abundance (%)	Fractional Abundance (%)	Quality Score	Migration Time (min)
AAV6	Empty	Unknown Component 1	62,511.32	3.42×10^{7}	0.07	0.03	58.33	3.529
		Unknown Component 2	60,095.74	9.10×10^{7}	0.19	0.08	47.35	3.360
		Unknown Component 3	59,474.15	1.88×10^{9}	4.00	1.63	58.10	3.413
		Unknown Component 4	43,908.72	4.17×10^{7}	0.09	0.04	66.82	2.942
		Unknown Component 5	26,140.07	7.05×10^{8}	1.50	0.61	40.43	3.025
		Unknown Component 6	26,069.70	6.09×10^{8}	1.29	0.53	45.50	3.107
		Unknown Component 7	26,054.67	3.52×10^{9}	7.48	3.04	54.01	3.100
		Unknown Component 8	26,050.64	4.43×10^{9}	9.40	3.83	56.89	3.082
		Unknown Component 9	26,034.46	6.62×10^{8}	1.41	0.57	38.69	3.100
		Unknown Component 10	26,012.37	1.42×10^{9}	3.02	1.23	49.52	3.022
		Unknown Component 11	26,008.53	2.25×10^{9}	4.78	1.95	53.28	3.030
		Unknown Component 12	25,993.43	5.90×10^{8}	1.25	0.51	38.41	3.065
	Full	Unknown Component 3	59,473.23	7.01×10^{8}	3.20	2.28	37.13	3.413
AAV8	Empty	Unknown Component 1	59,843.02	3.99×10^{8}	1.13	0.19	39.70	3.464
		Unknown Component 2	59,715.94	6.66×10^{8}	1.89	0.31	39.80	3.458
		Unknown Component 3	43,908.77	2.42×10^{8}	0.69	0.11	65.26	2.905
		Unknown Component 4	43,693.18	1.66×10^{7}	0.05	0.01	64.16	2.845
		Unknown Component 5	26,920.94	3.75×10^{9}	10.64	1.74	50.37	2.972
		Unknown Component 6	26,849.60	8.17×10^{8}	2.32	0.38	48.41	3.052
		Unknown Component 7	26,813.69	1.20×10^{9}	3.40	0.56	45.39	3.035
		Unknown Component 8	26,807.58	2.99×10^{8}	0.85	0.14	38.61	2.965
		Unknown Component 9	26,789.48	1.22×10^{10}	34.63	5.66	58.43	2.979
		Unknown Component 10	26,772.40	1.39×10^{9}	3.93	0.64	43.39	3.008
	Full	Unknown Component 1	59,844.37	3.65×10^{8}	1.47	0.73	38.43	3.432
		Unknown Component 2	59,716.00	4.72×10^{8}	1.90	0.95	43.16	3.431
AAV9	Empty	Unknown Component 1	59,687.55	2.75×10^{9}	5.64	3.78	63.39	3.492
		Unknown Component 2	26,140.48	7.65×10^{7}	0.16	0.10	41.86	3.017
		Unknown Component 3	26,069.54	5.67×10^{7}	0.12	0.08	45.45	3.078
		Unknown Component 4	26,054.57	3.02×10^{8}	0.62	0.42	52.09	3.089
		Unknown Component 5	26,050.51	4.40×10^{8}	0.90	0.60	58.23	3.091
		Unknown Component 6	26,012.41	1.23×10^{8}	0.25	0.17	47.35	3.010
		Unknown Component 7	26,008.34	1.74×10^{8}	0.36	0.24	55.66	3.018
	Full	Unknown Component 1	59,687.18	2.14×10^{9}	5.30	3.75	67.35	3.494

Table S7: Select peptides found in both empty and full AAV6 peptide mapping analysis to confirm presence of VPs and fragments detected during intact mass analysis. Tandem MS (MS/MS) spectra of the highlighted peptides is shown in Figures S2-S7 as illustrative examples of the quality of the peptides identified for each VP or fragment. Peptide information displayed is from the analysis of the full AAV6 capsids

Peptide Sequence	Positions	Modification	Site	Delta (ppm)	Confidence Score	ID Type	RT	M/Z	Charge State	Mono Mass Exp.	Mono Mass Theo.
**Acetylated VP1 (N-Term Peptide)**											
AADGYLPDW	2-10	Acetylation	A2	2.81	100	MS2	95.61	525.234	2	1,048.4531	1,048.4502
AADGYLPDWL	2-11	Acetylation	A2	2.37	100	MS2	104.64	581.776	2	1,161.537	1,161.5342
AADGYLPDWLED	2-13	Acetylation	A2	2.77	100	MS2	102.53	703.811	2	1,405.6077	1,405.6038
AADGYLPDWLEDNL	2-15	Acetylation	A2	2.43	100	MS2	106.04	817.375	2	1,632.7347	1,632.7308
AADGYLPDWLEDNLSE	2-17	Acetylation	A2	2.46	100	MS2	103.81	925.913	2	1,848.8099	1,848.8054
AADGYLPDWLEDNLSEG	2-18	Acetylation	A2	2.53	100	MS2	102.95	954.425	2	1,905.8317	1,905.8269
AADGYLPDWLEDNLSEGIRE	2-21	Acetylation	A2	2.47	100	MS2	91.72	1,153.539	2	2,304.0603	2,304.0546
**R116-VP1 Fragment (N-Term Peptide)**											
RAVFQAKKRVLEPFGL	116-131	-	-	3.02	100	MS2	49.14	465.783	4	1,858.0997	1858.0941
**VP2 (N-Term Peptide)**											
APGKKRPVEQSPQEPD	139-154	-	-	2.31	100	MS2	26.32	588.309	3	1,761.905	1,761.901
APGKKRPVEQSPQEPDSSSGIGKTGQQPAKKRLNFGQTGD	139-178	-	-	1.5	100	MS2	34.5	702.37	6	4,205.1641	4,205.1577
APGKKRPVEQSPQEPDSSSGIGKTGQQPAKKRLNFGQTGDSESVPD	139-184	-	-	2.75	100	MS2	37.79	689.926	7	4,819.4258	4,819.4125
APGKKRPVEQSPQEPDSSSGIGKTGQQPAKKRLNFGQTGDSESVPDPQPLGEPPATPAAVGPTTM	139-203	-	-	-1.14	100	MS2	51.34	1,107.064	6	6,632.313	6,632.3205
**Acetylated VP3 (N-Term Peptide)**											
ASGGGAPMADNNEGAD	204-219	Acetylation	A204	2.19	100	MS2	54.97	738.29	2	1,474.5663	1,474.5631
ASGGGAPMADNNEGADGVGNASGNW	204-228	Acetylation	A204	-2.31	100	MS2	75.71	1,159.973	2	2,316.9248	2,316.9302
ASGGGAPMADNNEGADGVGNASGNWHCDSTWLGD	204-237	Acetylation	A204	2.11	100	MS2	79.62	1,667.672	2	3,331.3237	3,331.3167
ASGGGAPMADNNEGADGVGNASGNWHCDSTWLGDRV	204-239	Acetylation	A204	2.89	100	MS2	71.97	1,197.175	3	3,586.4966	3,586.4862
ASGGGAPMADNNEGADGVGNASGNWHCDSTWLGDRVIT	204-241	Acetylation	A204	2.24	100	MS2	74.49	1,268.551	3	3,800.6265	3,800.618
ASGGGAPMADNNEGADGVGNASGNWHCDSTWLGDRVITT	204-242	Acetylation	A204	2.35	100	MS2	74.65	1,302.234	3	3,901.6748	3,901.6656
ASGGGAPMADNNEGADGVGNASGNWHCDSTWLGDRVITTST	204-244	Acetylation	A204	2.81	100	MS2	74.47	1,364.928	3	4,089.7568	4,089.7453
ASGGGAPMADNNEGADGVGNASGNWHCDSTWLGDRVITTSTRT	204-246	Acetylation	A204	3.01	100	MS2	67.98	1,450.643	3	4,346.9072	4,346.8941
ASGGGAPMADNNEGADGVGNASGNWHCDSTWLGDRVITTSTRTWAL	204-249	Acetylation	A204	1.99	100	MS2	78.27	1,574.379	3	4,717.104	4,717.0946
**Acetylated A212 VP3 Variant (N-Term Peptide)**											
ADNNEGADGVGNASGNWHCDSTWLGDRVITTSTRT	212-246	Acetylation	A212	3.38	100	MS2	66.69	1,240.889	3	3,718.6428	3,718.6302
ADNNEGADGVGNASGNWHCDSTWLGDRVITTSTRTWALPTYNNHL	212-256	Acetylation	A212	2.34	100	MS2	75.28	1,233.819	4	4,928.2349	4,928.2233
**Acetylated VP3-D590 Fragment (C-Term Peptide)**											
EEIKATNPVATERFGTVAVNLQSSSTD	564-590	-	-	1.36	100	MS2	60.49	955.817	3	2,863.4238	2,863.4199
VNLQSSSTD	582-590	-	-	2.04	100	MS2	38.34	475.726	2	949.4372	949.4353

Table S8: Select peptides found in both empty and full AAV8 peptide mapping analysis to confirm presence of VPs and fragments detected during intact mass analysis. MS/MS spectra of the highlighted peptides is shown in Figures S8-S14 as illustrative examples of the quality of the peptides identified for each VP or fragment. Peptide information displayed is from the analysis of the full AAV8 capsids

Peptide Sequence	Positions	Modification	Site	Delta (ppm)	Confidence Score	ID Type	RT	M/Z	Charge State	Mono Mass Exp.	Mono Mass Theo.
**Acetylated VP1 (N-Term Peptide)**											
**AADGYLPDWL**	2-11	Acetylation	A2	3.52	100	MS2	104.56	581.776	2	1,161.5383	1,161.5342
**AADGYLPDWLED**	2-13	Acetylation	A2	3.03	100	MS2	102.1	703.811	2	1,405.608	1,405.6038
**AADGYLPDWLEDNL**	2-15	Acetylation	A2	1.76	100	MS2	105.81	817.374	2	1,632.7336	1,632.7308
**AADGYLPDWLEDNLSE**	2-17	Acetylation	A2	2.99	100	MS2	103.44	925.413	2	1,848.8109	1,848.8054
**AADGYLPDWLEDNLSEG**	2-18	Acetylation	A2	2.78	100	MS2	102.64	953.923	2	1,905.8322	1,905.8269
**AADGYLPDWLEDNLSEGIRE**	2-21	Acetylation	A2	3.1	100	MS2	91.89	769.362	3	2,304.0618	2,304.0546
**V132-VP1 Fragment (N-Term Peptide)**											
**VEEGAKTAPGKKRPVEPSPQRSPD**	132-155	-	-	2.22	100	MS2	28.06	641.095	4	2,559.3462	2,559.3405
**VEEGAKTAPGKKRPVEPSPQRSPDSSTGIGKKGQQPARKRLNFGQTGDSESVPD**	132-185	Phosphorylation	~S149	-0.47	100	MS2	37.19	962.327	6	5,765.8848	5,765.8875
**VP2 (N-Term Peptide)**											
**APGKKRPVEPSPQRSPDSSTGIGKKGQQPARKRLNFGQTGDSESVPD**	139-185	Phosphorylation	~S149	4.94	100	MS2	37.2	723.228	7	5,051.5576	5,051.5327
**Acetylated VP3 (N-Term Peptide)**											
**AAGGGAPMADNNEGADGVGSSSGNW**	205-229	Acetylation	A205	3.38	100	MS2	76.02	1,146.472	2	2,289.927	2,289.9193
**AAGGGAPMADNNEGADGVGSSSGNWHCDSTWLGD**	205-238	Acetylation	A205	4.76	100	MS2	79.89	1,654.17	2	3,304.3215	3,304.3058
**AAGGGAPMADNNEGADGVGSSSGNWHCDSTWLGDRVIT**	205-242	Acetylation	A205	3.2	100	MS2	74.98	1,259.215	3	3,773.6191	3,773.6071
**AAGGGAPMADNNEGADGVGSSSGNWHCDSTWLGDRVITT**	205-243	Acetylation	A205	3.67	100	MS2	75.1	1,293.232	3	3,874.6689	3,874.6547
**AAGGGAPMADNNEGADGVGSSSGNWHCDSTWLGDRVITTST**	205-245	Acetylation	A205	1.78	100	MS2	74.91	1,355.589	3	4,062.7417	4,062.7344
**AAGGGAPMADNNEGADGVGSSSGNWHCDSTWLGDRVITTSTRT**	205-247	Acetylation	A205	4.2	100	MS2	68.43	1,441.641	3	4,319.9014	4,319.8832
**AAGGGAPMADNNEGADGVGSSSGNWHCDSTWLGDRVITTSTRTWAL**	205-250	Acetylation	A205	4.01	100	MS2	78.82	1,174.034	4	4,690.1025	4,690.0837
**Unmodified VP3 (N-Term Peptide)**											
**AAGGGAPMADNNEGAD**	205-220	-	-	0.72	100	MS2	39.65	709.287	2	1,416.5586	1,416.5576
**AAGGGAPMADNNEGADGVGSSSGNW**	205-229	-	-	3.04	100	MS2	59.06	1,125.467	2	2,247.9155	2,247.9087
**AAGGGAPMADNNEGADGVGSSSGNWHCDSTWLGD**	205-238	-	-	3.05	100	MS2	68.36	1,088.777	3	3,262.3052	3,262.2952
**AAGGGAPMADNNEGADGVGSSSGNWHCDSTWLGDRVIT**	205-242	-	-	4.24	100	MS2	66.89	1,245.546	3	3,731.6123	3,731.5965
**AAGGGAPMADNNEGADGVGSSSGNWHCDSTWLGDRVITT**	205-243	-	-	4.04	100	MS2	67.18	1,279.229	3	3,832.6597	3,832.6442
**AAGGGAPMADNNEGADGVGSSSGNWHCDSTWLGDRVITTST**	205-245	-	-	3.76	100	MS2	67.17	1,341.922	3	4,020.739	4,020.7239
**AAGGGAPMADNNEGADGVGSSSGNWHCDSTWLGDRVITTSTRT**	205-247	-	-	3.63	100	MS2	62.22	1,070.979	4	4,277.8882	4,277.8727
**AAGGGAPMADNNEGADGVGSSSGNWHCDSTWLGDRVITTSTRTWALPTYNNHL**	205-257	-	-	4.99	100	MS2	72.07	1,373.382	4	5,487.4932	5,487.4658
**G209-VP3 Fragment (N-Term Peptide)**											
**GAPMADNNEGADGVGSSSGNWHCDSTWLGDRVIT**	209-242	-	-	2.36	100	MS2	67.14	1,159.839	3	3,475.4875	3,475.4793

Peptide Sequence	Positions	Modification	Site	Delta (ppm)	Confidence Score	ID Type	RT	M/Z	Charge State	Mono Mass Exp.	Mono Mass Theo.
**Acetylated A213 VP3 Variant (N-Term Peptide)**											
**ADNNEGADGVGSSSGNWHCDSTWLGDRVITTSTRT**	213-247	Acetylation	A213	3.89	100	MS2	66.57	1,237.552	3	3,707.6287	3,707.6143
**ADNNEGADGVGSSSGNWHCDSTWLGDRVITTSTRTWALPTYNNHL**	213-257	Acetylation	A213	3.91	100	MS2	75.9	1,231.066	4	4,917.2266	4,917.2073

Table S9: Select peptides found in both empty and full AAV9 peptide mapping analysis to confirm presence of VPs and fragments detected during intact mass analysis. MS/MS spectra of the highlighted peptides is shown in Figures S15-S26 as illustrative examples of the quality of the peptides identified for each VP or fragment. Peptide information displayed is from the analysis of the full AAV9 capsids

Peptide Sequence	Positions	Modification	Site	Delta (ppm)	Confidence Score	ID Type	RT	M/Z	Charge State	Mono Mass Exp.	Mono Mass Theo.
**Acetylated VP1 (N-Term Peptide)**											
**AADGYLPDW**	2-10	Acetylation	A2	4.21	100	MS2	95.69	525.235	2	1,048.4546	1,048.4502
**AADGYLPDWL**	2-11	Acetylation	A2	4.26	100	MS2	104.69	581.777	2	1,161.5392	1,161.5342
**AADGYLPDWLED**	2-13	Acetylation	A2	3.38	100	MS2	102.5	703.812	2	1,405.6085	1,405.6038
**AADGYLPDWLEDNL**	2-15	Acetylation	A2	2.73	100	MS2	106.07	817.375	2	1,632.7352	1,632.7308
**AADGYLPDWLEDNLSE**	2-17	Acetylation	A2	3.52	100	MS2	103.8	925.914	2	1,848.8119	1,848.8054
**AADGYLPDWLEDNLSEG**	2-18	Acetylation	A2	3.23	100	MS2	102.98	954.425	2	1,905.833	1,905.8269
**AADGYLPDWLEDNLSEGIRE**	2-21	Acetylation	A2	3.42	100	MS2	91.81	769.363	3	2,304.0625	2,304.0546
**AADGYLPDWLEDNLSEGIREW**	2-22	Acetylation	A2	4.55	100	MS2	96.83	831.388	3	2,490.1453	2,490.1339
**R116-VP1 Fragment (N-Term Peptide)**											
**RAVFQAKKRLLEPLGL**	116-131	-	-	2.83	100	MS2	50.8	614.052	3	1,838.1306	1,838.1254
**L131-VP1 Fragment (N-Term Peptide)**											
**LVEEAAKTAPGKKRPVE**	131-147	-	-	4.49	100	MS2	29.79	456.517	4	1,822.0394	1,822.0312
**LVEEAAKTAPGKKRPVEQSPQEPD**	131-154	-	-	2.62	100	MS2	33.76	652.099	4	2,603.3623	2,603.3555
**LVEEAAKTAPGKKRPVEQSPQEPDSSAGIGKSGAQPAKKRLNFGQTGDTESVPD**	131-184	-	-	-0.28	100	MS2	40.58	699.741	8	5,587.8491	5,587.8507
**VP2 (N-Term Peptide)**											
**APGKKRPVEQSPQEPD**	139-154	-	-	3	100	MS2	26.32	588.309	3	1,761.9062	1,761.901
**APGKKRPVEQSPQEPDSSAGIGKSGAQPAKKRLNFGQTGDTESVPD**	139-184	Phosphorylation	S149	3.1	100	MS2	40.05	805.905	6	4,826.3774	4,826.3625
**APGKKRPVEQSPQEPDSSAGIGKSGAQPAKKRLNFGQTGDTESVPDPQPIGEPPAAPSGVGSL**	139-201	-	-	2.46	100	MS2	51.29	901.612	7	6,301.2158	6,301.2003
**APGKKRPVEQSPQEPDSSAGIGKSGAQPAKKRLNFGQTGDTESVPDPQPIGEPPAAPSGVGSLTM**	139-203	-	-	-1.11	100	MS2	53.56	1,090.561	6	6,533.2812	6,533.2885
**F173-VP2 Fragment (N-Term Peptide)**											
**FGQTGDTESVPD**	173-184	-	-	3.89	100	MS2	49.78	626.772	2	1,251.5304	1,251.5255
**FGQTGDTESVPDPQPIGEPPAAPSGVGSL**	173-201	-	-	4.82	100	MS2	75.78	936.788	3	2,806.3433	2,806.3297
**M203-VP3 (N-Term Peptide)**											
**MASGGGAPVADNNEGAD**	203-219	-	-	2.76	100	MS2	41.04	766.82	2	1,531.6251	1,531.6209
**MASGGGAPVADNNEGADGVGSSSGNWHCDSQWLGD**	203-237	-	-	2.99	100	MS2	66.57	1,136.135	3	3,404.3796	3,404.3695

Peptide Sequence	Positions	Modification	Site	Delta (ppm)	Confidence Score	ID Type	RT	M/Z	Charge State	Mono Mass Exp.	Mono Mass Theo.
MASGGGAPVADNNEGADGVGSSSGNWHCDSQWLGDRVITT	203-242	-	-	3.65	100	MS2	65.47	995.193	4	3,974.7329	3,974.7184
MASGGGAPVADNNEGADGVGSSSGNWHCDSQWLGDRVITTSTRT	203-246	-	-	3.29	100	MS2	60.64	1,106.498	4	4,419.9614	4,419.9469
**Acetylated VP3 (N-Term Peptide)**											
ASGGGAPVAD	204-213	Acetylation	A204	0.44	98.5	MS2	51.57	422.196	2	842.3774	842.377
ASGGGAPVADNNEGAD	204-219	Acetylation	A204	3.44	100	MS2	52.11	722.305	2	1,442.5959	1,442.591
ASGGGAPVADNNEGADGVGSSSGNW	204-228	Acetylation	A204	3.23	100	MS2	72.79	1,138.483	2	2,273.9495	2,273.9421
ASGGGAPVADNNEGADGVGSSSGNWHCD	204-231	Acetylation	A204	4.35	100	MS2	60.7	877.691	3	2,629.0486	2,629.0371
ASGGGAPVADNNEGADGVGSSSGNWHCDSQ	203-233	Acetylation	A204	2.89	100	MS2	58.38	949.387	3	2,844.136	2,844.1278
ASGGGAPVADNNEGADGVGSSSGNWHCDSQWLGD	204-237	Acetylation	A204	4.07	100	MS2	76.23	1,659.186	2	3,315.353	3,315.3395
ASGGGAPVADNNEGADGVGSSSGNWHCDSQWLGDRV	204-239	Acetylation	A204	4.03	100	MS2	69.24	1,191.516	3	3,570.5234	3,570.5091
ASGGGAPVADNNEGADGVGSSSGNWHCDSQWLGDRVIT	204-241	Acetylation	A204	4.73	100	MS2	71.95	1,263.227	3	3,784.6587	3,784.6408
ASGGGAPVADNNEGADGVGSSSGNWHCDSQWLGDRVITT	204-242	Acetylation	A204	3.64	100	MS2	72.14	972.934	4	3,885.7026	3,885.6885
ASGGGAPVADNNEGADGVGSSSGNWHCDSQWLGDRVITTST	204-244	Acetylation	A204	2.37	100	MS2	72.03	1,019.954	4	4,073.7778	4,073.7682
ASGGGAPVADNNEGADGVGSSSGNWHCDSQWLGDRVITTSTRT	204-246	Acetylation	A204	3.61	100	MS2	65.76	1,445.319	3	4,330.9326	4,330.917
ASGGGAPVADNNEGADGVGSSSGNWHCDSQWLGDRVITTSTRTWAL	204-249	Acetylation	A204	4.3	100	MS2	76.39	1,176.792	4	4,701.1377	4,701.1175
ASGGGAPVADNNEGADGVGSSSGNWHCDSQWLGDRVITTSTRTWALPTYNNHL	204-256	Acetylation	A204	3.03	100	MS2	74.12	1,109.714	5	5,540.5269	5,540.5101
**Acetylated VP3-D657 Fragment (C-Term Peptide)**											
AKIPHTDGNFHPSPLMGGFGMKHPPPQILIKNTPVPAD	620-657	-	-	3.74	100	MS2	58.27	677.358	6	4,056.1018	4,056.0866
GNFHPSPLMGGFGMKHPPPQILIKNTPVPAD	627-657	-	-	2.64	100	MS2	61.07	659.947	5	3,293.6929	3,293.6842
IKNTPVPAD	649-657	-	-	0.25	100	MS2	35.28	477.766	2	953.5184	953.5182
**Acetylated VP3-S538 Fragment (C-Term Peptide)**											
ALNGRNSLMNPGPAMASHKEGEDRFFPLS	510-538	-	-	2.06	100	MS2	57.13	629.912	5	3,142.5142	3,142.5077
**Acetylated VP3-M518 Fragment (C-Term Peptide)**											
FAWPGASSWALNGRNSLM	501-518	-	-	3.53	100	MS2	78.26	982.979	2	1,963.9432	1,963.9363
WALNGRNSLM	509-518	-	-	4.09	100	MS2	58.8	581.298	2	1,160.5808	1,160.5761
ALNGRNSLM	510-518	-	-	4.02	100	MS2	41.58	488.258	2	974.5007	974.4967
**Acetylated VP3-N512 Fragment (C-Term Peptide)**											
AVQGRNYIPGPSYRQQRVSTTVTQNNNSEFAWPGASSWALN	472-512	-	-	4.45	100	MS2	64.85	1,139.314	4	4,551.2271	4,551.2068
**Acetylated VP3-S448 Fragment (C-Term Peptide)**											
YLYYLS	443-448	-	-	-2.62	99.3	MS2	68.43	821.406	1	820.3986	820.4007
YYLS	445-448	-	-	2.43	99.0	MS2	46.94	545.262	1	544.2546	544.2533

Table S10: Acetylation and phosphorylation PTMs identified during peptide mapping of AAV6 empty and full capsids with a relative abundance ≥ 1%. ~ indicates that the exact location of the modification could not be determined by BPF 5.1, but the modification is near or on the listed residue

Modification	Amino Acid Residues	Most Abundant Peptide Sequence Containing Modification	Confidence	Average % abundance	STDEV
		**Empty Capsids**			
**A2+Acetylation**	A2-L15	AADGYLPDWLEDNL	100.00	100.00	0.00
**A204+Acetylation**	A204-L255	ASGGGAPMADNNEGADGVGNASGNWHCDSTWLGDRVITTSTRTWALPTYNNHL	100.00	97.75	0.39
**~S149+Phosphorylation**	A139-M203	APGKKRPVEQSPQEPDSSSGIGKTGQQPAKKRLNFGQTGDSESVPDPQPLGEPPATPAAVGPTTM	100.00	13.11	0.29
**~S467+Phosphorylation**	F463-F501	FSRGSPAGMSVQPKNWLPGPCYRQQRVSKTKTDNNNSNF	100.00	1.83	0.14
**~S149+Phosphorylation**	V132-D184	VEEGAKTAPGKKRPVEQSPQEPDSSSGIGKTGQQPAKKRLNFGQTGDSESVPD	99.99	10.07	0.42
**S149+Phosphorylation**	A139-D184	APGKKRPVEQSPQEPDSSSGIGKTGQQPAKKRLNFGQTGDSESVPD	100.00	6.83	2.38
**S467+Phosphorylation**	F463-M471	FSRGSPAGM	100.00	1.02	0.03
		**Full Capsids**			
**A2+Acetylation**	A2-L15	AADGYLPDWLEDNL	100.00	100.00	0.00
**A204+Acetylation**	A204-T246	ASGGGAPMADNNEGADGVGNASGNWHCDSTWLGDRVITTSTRT	100.00	98.13	0.14
**~S149+Phosphorylation**	A139-M203	APGKKRPVEQSPQEPDSSSGIGKTGQQPAKKRLNFGQTGDSESVPDPQPLGEPPATPAAVGPTTM	100.00	13.83	2.25
**S149+Phosphorylation**	A139-D184	APGKKRPVEQSPQEPDSSSGIGKTGQQPAKKRLNFGQTGDSESVPD	100.00	10.94	0.79
**S467+Phosphorylation**	F463-M471	FSRGSPAGM	100.00	1.18	0.06
**~T138+Phosphorylation**	V132-D184	VEEGAKTAPGKKRPVEQSPQEPDSSSGIGKTGQQPAKKRLNFGQTGDSESVPD	100.00	5.47	0.85
**~T494+Phosphorylation**	F463-F501	FSRGSPAGMSVQPKNWLPGPCYRQQRVSKTKTDNNNSNF	100.00	1.83	0.14

Table S11: Acetylation and phosphorylation PTMs identified during peptide mapping of AAV8 empty and full capsids with a relative abundance ≥ 1%. ~ indicates that the exact location of the modification could not be determined by BPF 5.1, but the modification is near or on the listed residue

Modification	Amino Acid Residues	Most Abundant Peptide Sequence Containing Modification	Confidence	Average % abundance	STDEV
		**Empty Capsids**			
**A2+Acetylation**	A2-L15	AADGYLPDWLEDNL	100.00	100.00	0.00
**A205+Acetylation**	A205-L257	AAGGGAPMADNNEGADGVGSSSGNWHCDSTWLGDRVITTSTRTWALPTYNNHL	100.00	60.87	0.87
**~S153+Phosphorylation**	A139-D185	APGKKRPVEPSPQRSPDSSTGIGKKGQQPARKRLNFGQTGDSESVPD	100.00	62.70	2.21
**~T138+Phosphorylation**	V132-D185	VEEGAKTAPGKKRPVEPSPQRSPDSSTGIGKKGQQPARKRLNFGQTGDSESVPD	100.00	46.44	0.63
**~T494+Phosphorylation**	Y446-F503	YYLSRTQTTGGTANTQTLGFSQGGPNTMANQAKNWLPGPCYRQQRVSTTTGQNNNSNF	100.00	1.34	0.11
		**Full Capsids**			
**A2+Acetylation**	A2-L15	AADGYLPDWLEDNL	100.00	99.45	0.02
**A205+Acetylation**	A205-T247	AAGGGAPMADNNEGADGVGSSSGNWHCDSTWLGDRVITTSTRT	100.00	73.64	0.70
**~S149+Phosphorylation**	V132-D185	VEEGAKTAPGKKRPVEPSPQRSPDSSTGIGKKGQQPARKRLNFGQTGDSESVPD	100.00	87.53	1.35

Table S12: Acetylation and phosphorylation PTMs identified during peptide mapping of AAV9 empty and full capsids with a relative abundance ≥ 1%. ~ indicates that the exact location of the modification could not be determined by BPF 5.1, but the modification is near or on the listed residue

Modification	Amino Acid Residues	Most Abundant Peptide Sequence Containing Modification	Confidence	Average % abundance	STDEV
		**Empty Capsids**			
**A2+Acetylation**	A2-L15	AADGYLPDWLEDNL	100.00	100.00	0.00
**A204+Acetylation**	A204-L256	ASGGGAPVADNNEGADGVGSSSGNWHCDSQWLGDRVITTSTRTWALPTYNNHL	100.00	97.39	0.13
**S149+Phosphorylation**	V132-D184	VEEAAKTAPGKKRPVEQSPQEPDSSAGIGKSGAQPAKKRLNFGQTGDTESVPD	100.00	14.31	0.09
**S149+Phosphorylation**	A139-D184	APGKKRPVEQSPQEPDSSAGIGKSGAQPAKKRLNFGQTGDTESVPD	100.00	13.03	0.93
**~S490+Phosphorylation**	A472-E500	AVQGRNYIPGPSYRQQRVSTTVTQNNNSE	100.00	3.62	0.14
		**Full Capsids**			
**A2+Acetylation**	A2-L15	AADGYLPDWLEDNL	100.00	100.00	0.00
**A204+Acetylation**	A204-L256	ASGGGAPVADNNEGADGVGSSSGNWHCDSQWLGDRVITTSTRTWALPTYNNHL	100.00	98.01	0.01
**~S149+Phosphorylation**	A139-L201	APGKKRPVEQSPQEPDSSAGIGKSGAQPAKKRLNFGQTGDTESVPDPQPIGEPPAAPSGVGSL	100.00	11.58	1.23
**~S490+Phosphorylation**	A472-E500	AVQGRNYIPGPSYRQQRVSTTVTQNNNSE	100.00	2.96	0.05
**~S149+Phosphorylation**	V132-D184	VEEAAKTAPGKKRPVEQSPQEPDSSAGIGKSGAQPAKKRLNFGQTGDTESVPD	100.00	13.78	0.30
**~S149+Phosphorylation**	A139-M203	APGKKRPVEQSPQEPDSSAGIGKSGAQPAKKRLNFGQTGDTESVPDPQPIGEPPAAPSGVGSLTM	100.00	16.60	0.66
**~S483+Phosphorylation**	S464-E500	SVAGPSNMAVQGRNYIPGPSYRQQRVSTTVTQNNNSE	100.00	1.06	0.04

Figure S1 Plot of total ion electropherogram area (blue line on top) and extracted apex MS signal intensity (orange line on bottom) versus Vps injected during LoD study

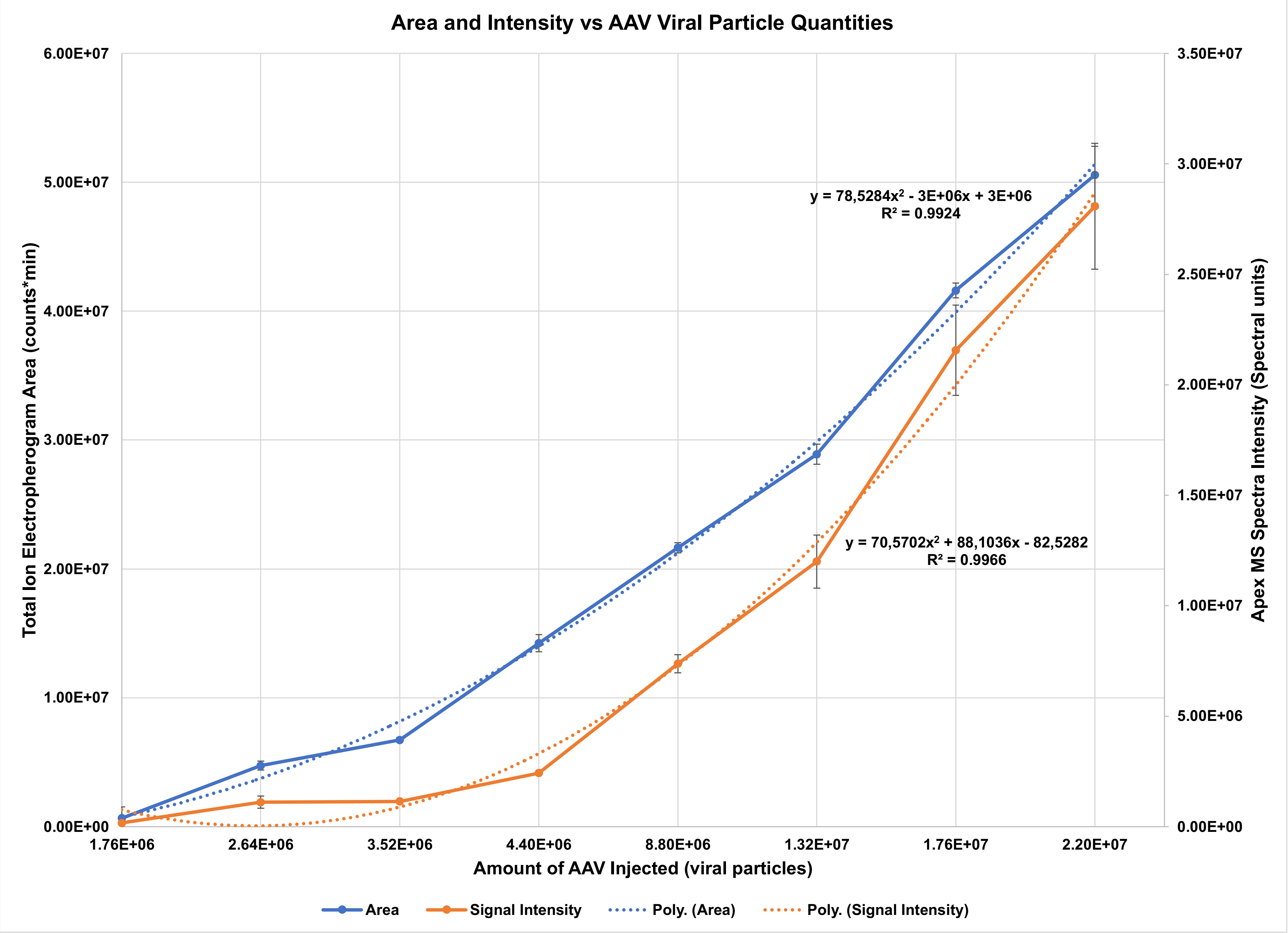

Figure S2 Tandem MS (MS/MS) spectra of the *AADGYLPDWLEDNLSEGIRE* (A2-E21) peptide, with acetylation at A2, from peptide mapping of full AAV6 capsids, as identified using BPF 5.1. Peptide is an example of the detected peptides that indicate the presence of the (Ac)VP1 capsid protein identified during intact mass analysis (Table S7)

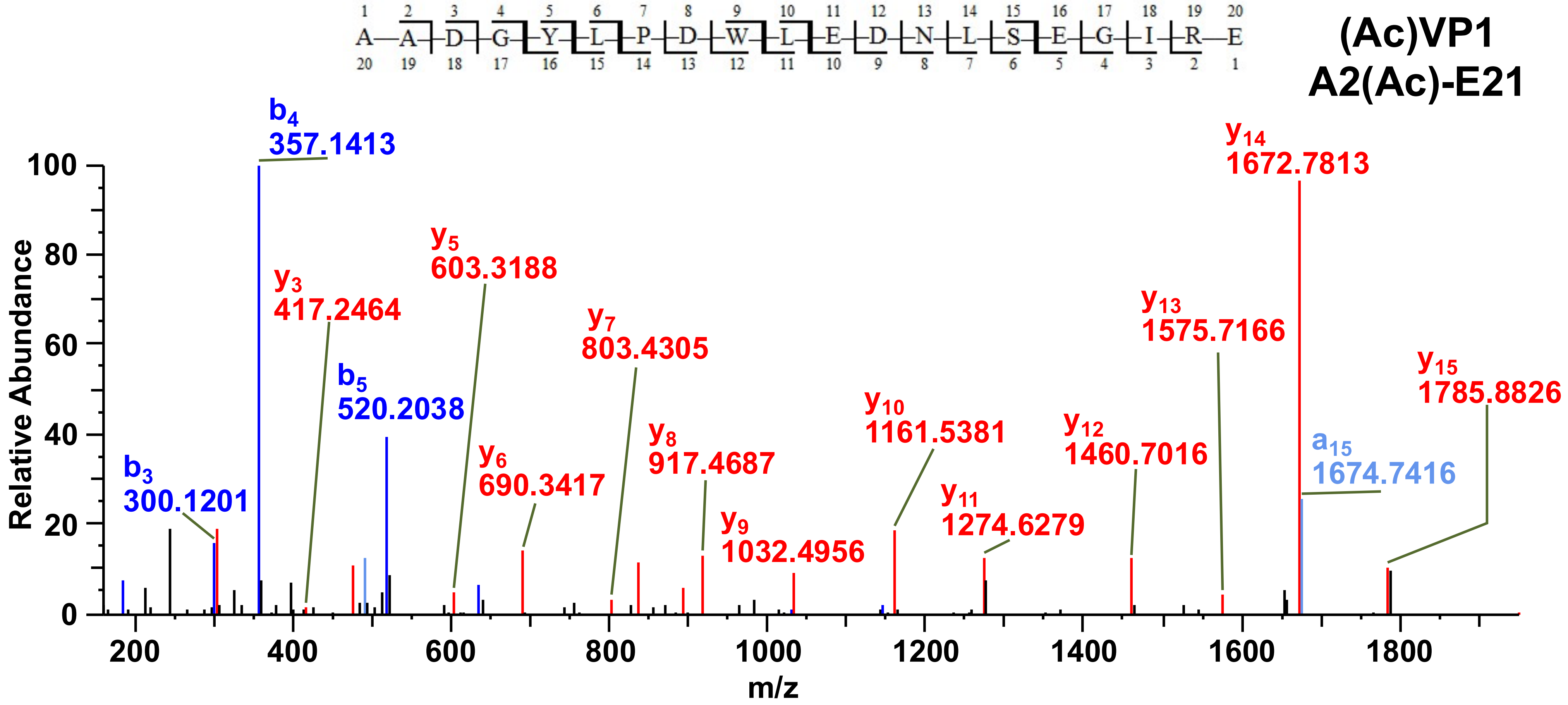

Figure S3 MS/MS spectra of the *APGKKRPVEQSPQEPDSSSGIGKTGQQPAKKRLNFGQTGD* (A139-D178) peptide from peptide mapping of full AAV6 capsids, as identified using BPF 5.1. Peptide is an example of the detected peptides that indicate the presence of the VP2 capsid protein identified during intact mass analysis (Table S7)

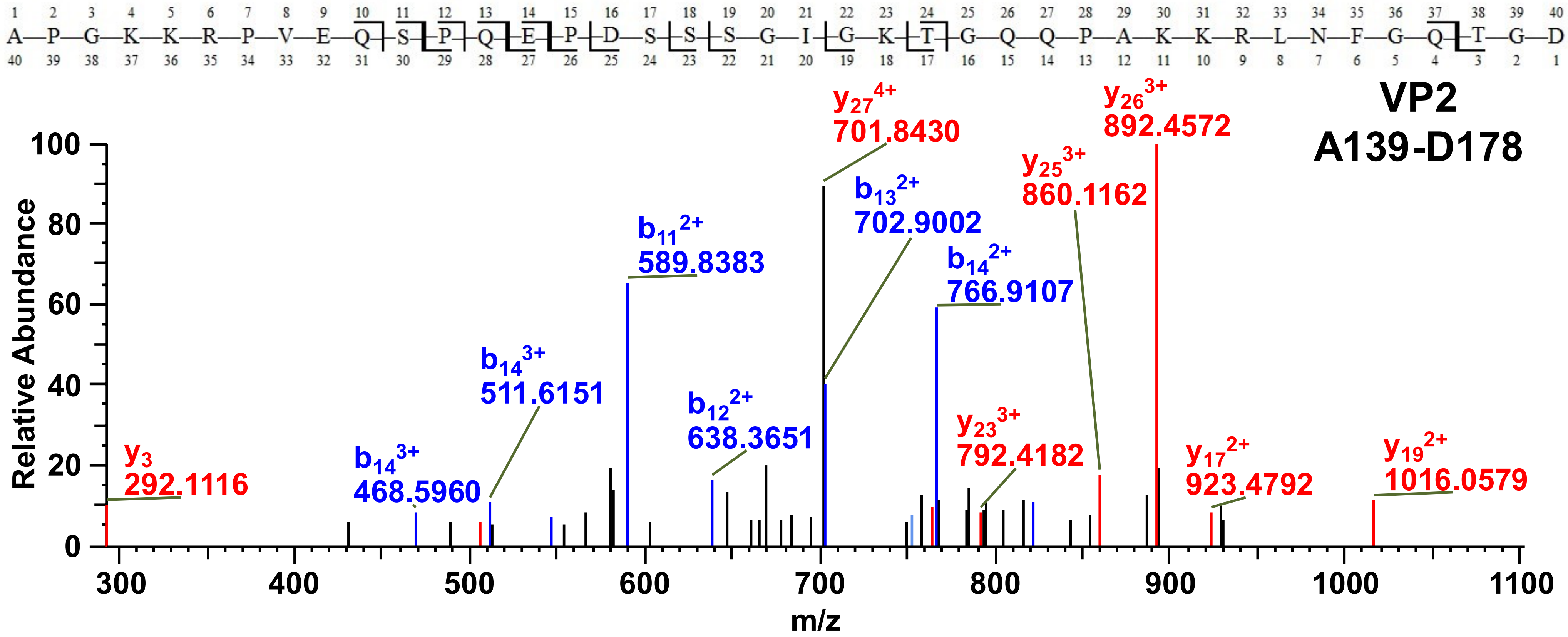

Figure S4 MS/MS spectra of the *ASGGGAPMADNNEGADGVGNASGNWHCDSTWLGDRVITT* (A204-T242) peptide, with acetylation at A204, from peptide mapping of full AAV6 capsids, as identified using BPF 5.1. Peptide is an example of the detected peptides that indicate the presence of the VP3 capsid protein identified during intact mass analysis (Table S7)

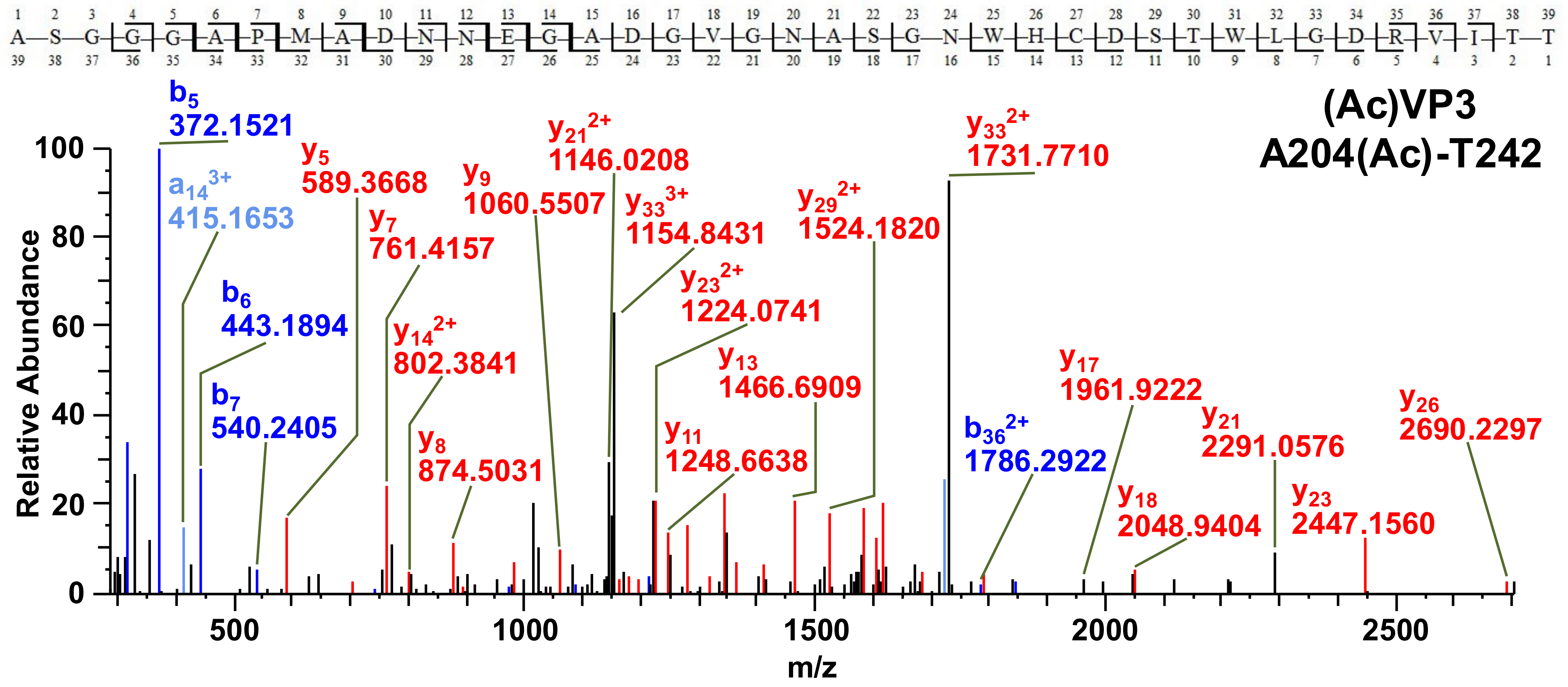

Figure S5 MS/MS spectra of the *ADNNEGADGVGNASGNWHCDSTWLGDRVITTSTRT* (A212-T246) peptide, with acetylation at A212, from peptide mapping of full AAV6 capsids, as identified using BPF 5.1. Peptide is an example of the detected peptides that indicate the presence of the (Ac)VP3 Variant capsid protein identified during intact mass analysis (Table S7)

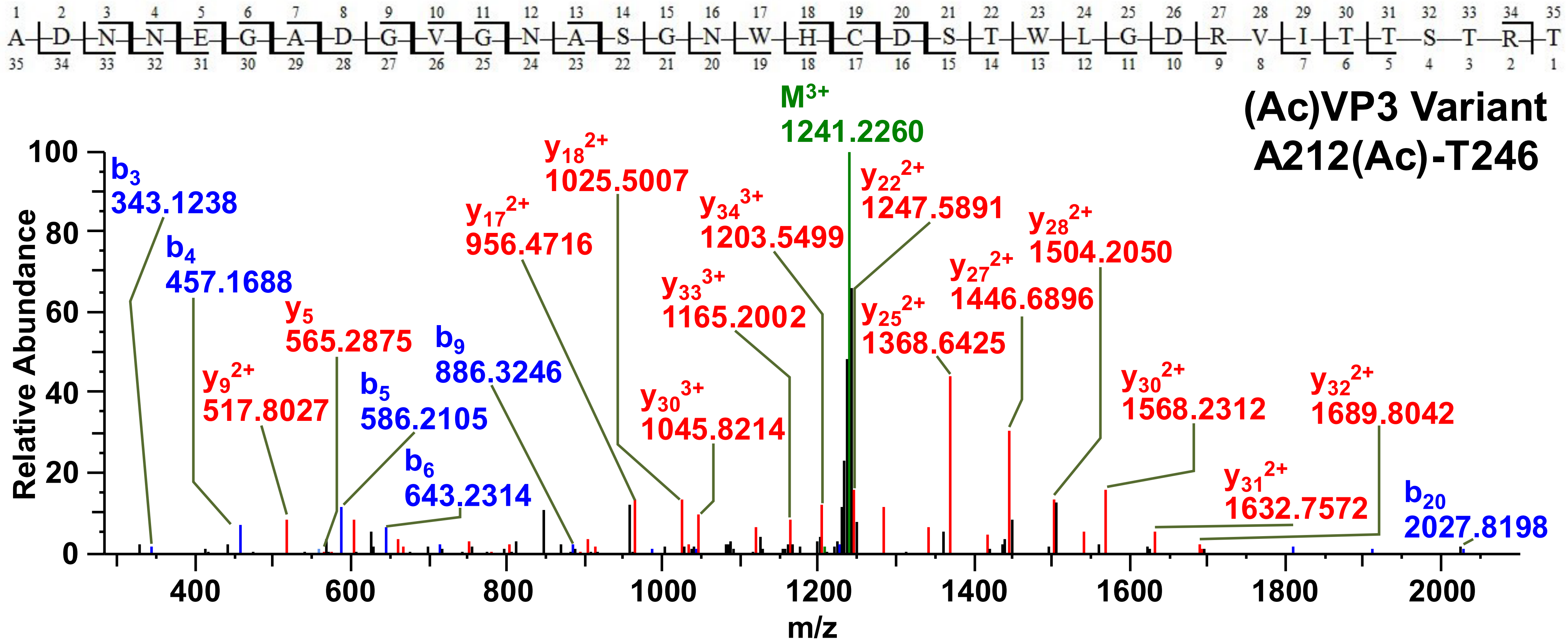

Figure S6 MS/MS spectra of the *RAVFQAKKRVLEPFGL* (R116-L131) peptide from peptide mapping of full AAV6 capsids, as identified using BPF 5.1. Peptide is an example of the detected peptides that indicate the presence of the R116-VP1 fragment identified during intact mass analysis (Table S7)

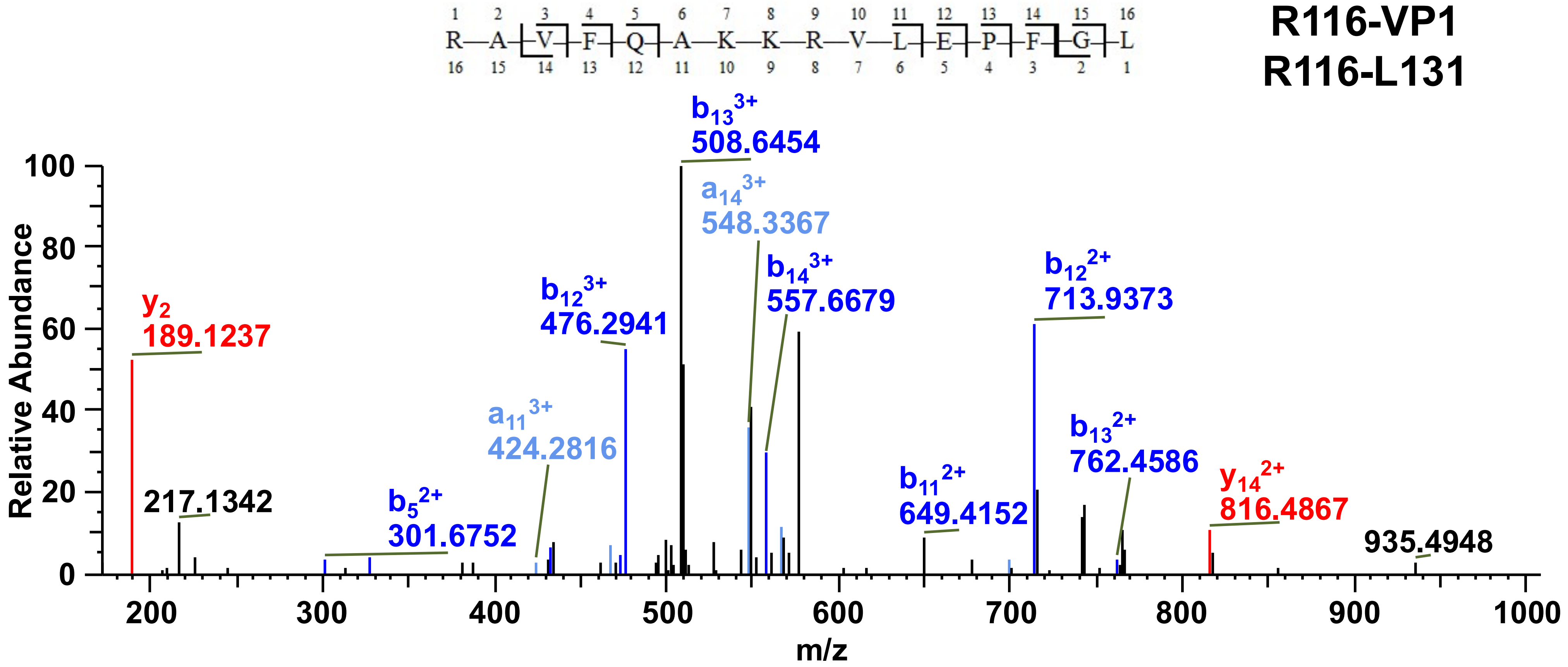

Figure S7 MS/MS spectra of the *EEIKATNPVATERFGTVAVNLQSSSTD* (E564-D590) peptide from peptide mapping of full AAV6 capsids, as identified using BPF 5.1. Peptide is an example of the detected peptides that indicate the presence of the (Ac)VP3-D590 fragment identified during intact mass analysis (Table S7)

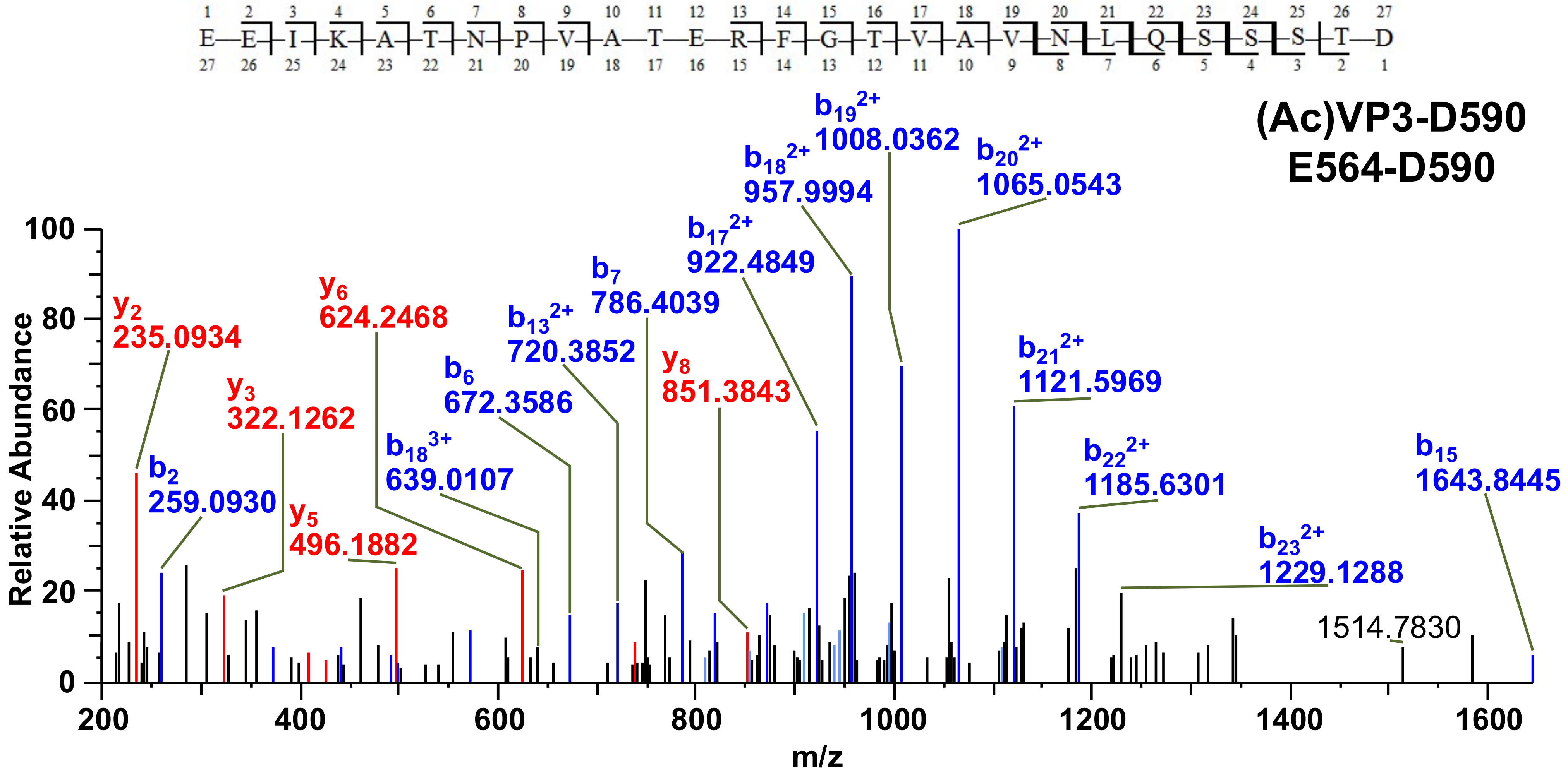

Figure S8 MS/MS spectra of the *AADGYLPDWLEDNLSEGIRE* (A2-E21) peptide, with acetylation at A2, from peptide mapping of full AAV8 capsids, as identified using BPF 5.1. Peptide is an example of the detected peptides that indicate the presence of the (Ac)VP1 capsid protein identified during intact mass analysis (Table S8)

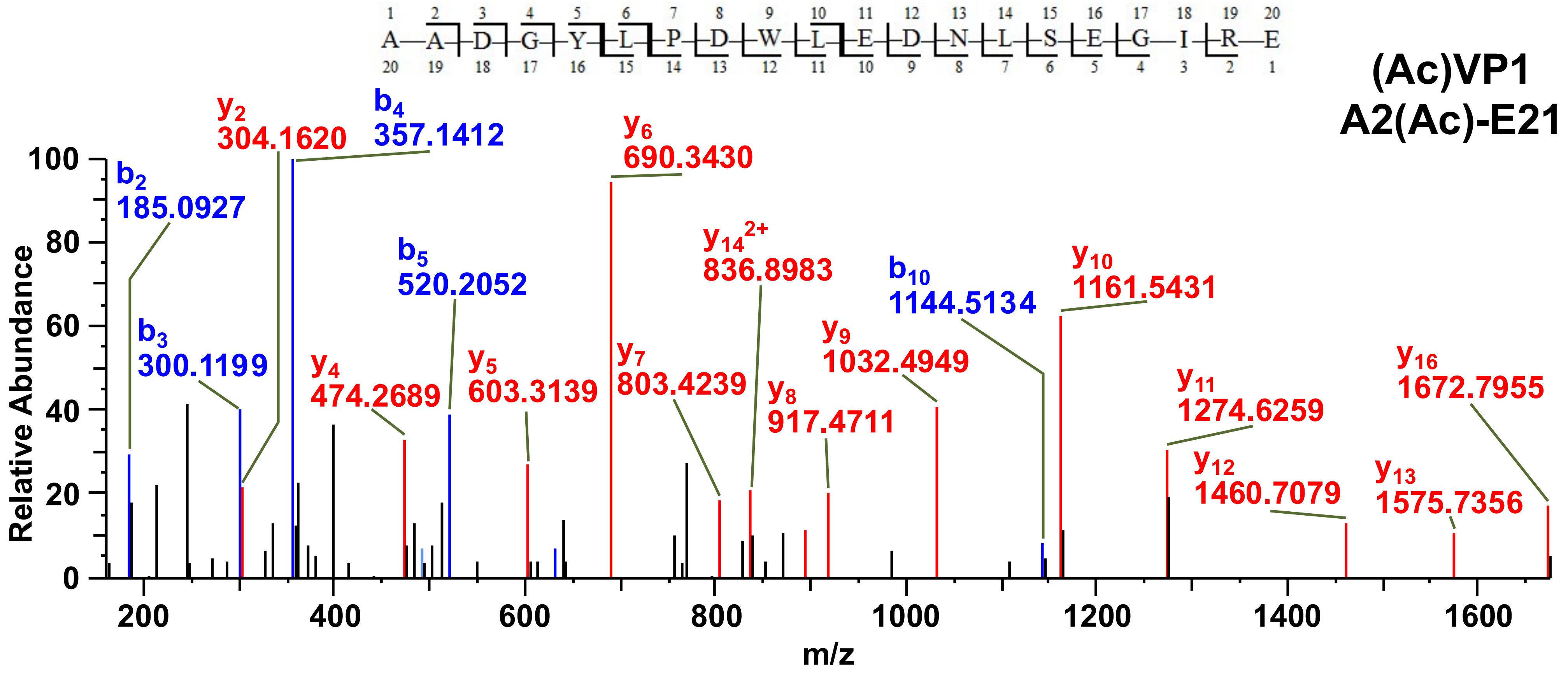

Figure S9 MS/MS spectra of the *APGKKRPVEPSPQRSPDSSTGIGKKGQQPARKRLNFGQTGDSESVPD* (A139-D185) peptide, with phosphorylation around S149 (exact location could not be determined), from peptide mapping of full AAV8 capsids, as identified using BPF 5.1. Peptide is an example of the detected peptides that indicate the presence of the VP2 capsid protein identified during intact mass analysis (Table S8). The relatively poor spectrum is a result of the phosphorylation present which impacts digestion efficiency of the pepsin, resulting in the large peptide identified

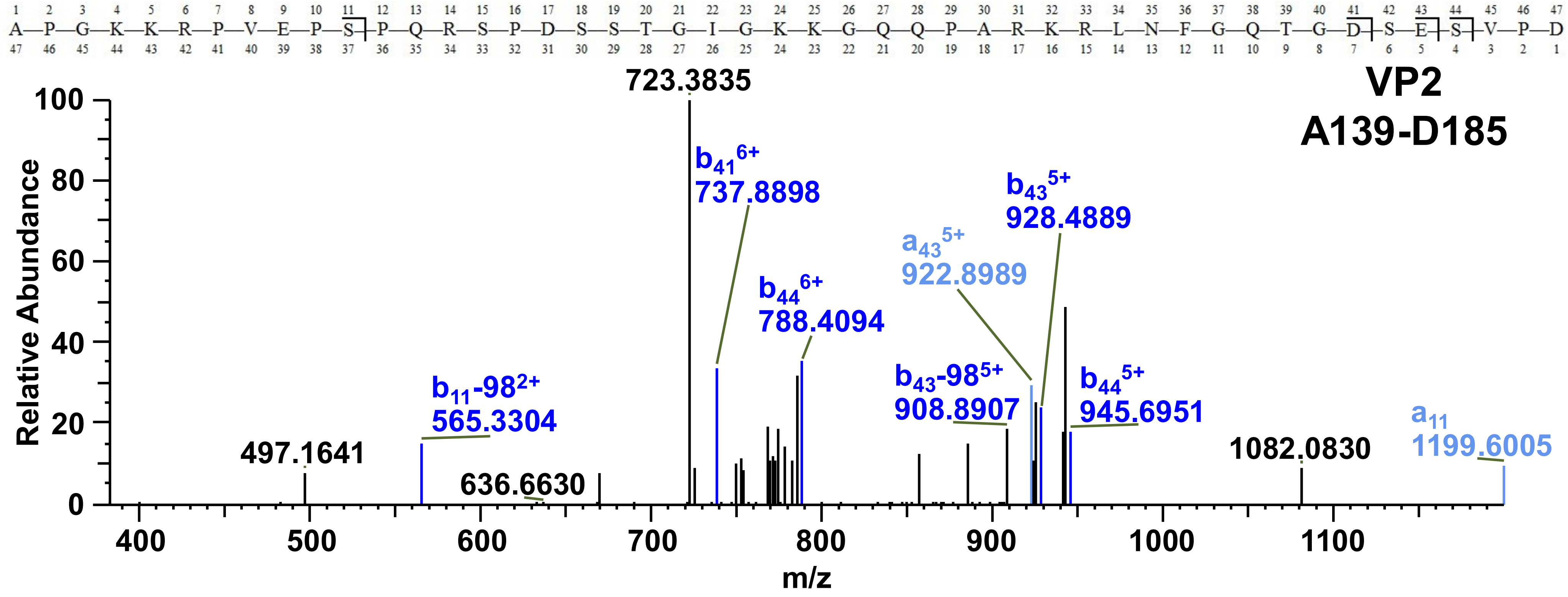

Figure S10 MS/MS spectra of the *AAGGGAPMADNNEGADGVGSSSGNWHCDSTWLGDRVITT* (A205-T243) peptide, with acetylation at A205, from peptide mapping of full AAV8 capsids, as identified using BPF 5.1. Peptide is an example of the detected peptides that indicate the presence of the (Ac)VP3 capsid protein identified during intact mass analysis (Table S8)

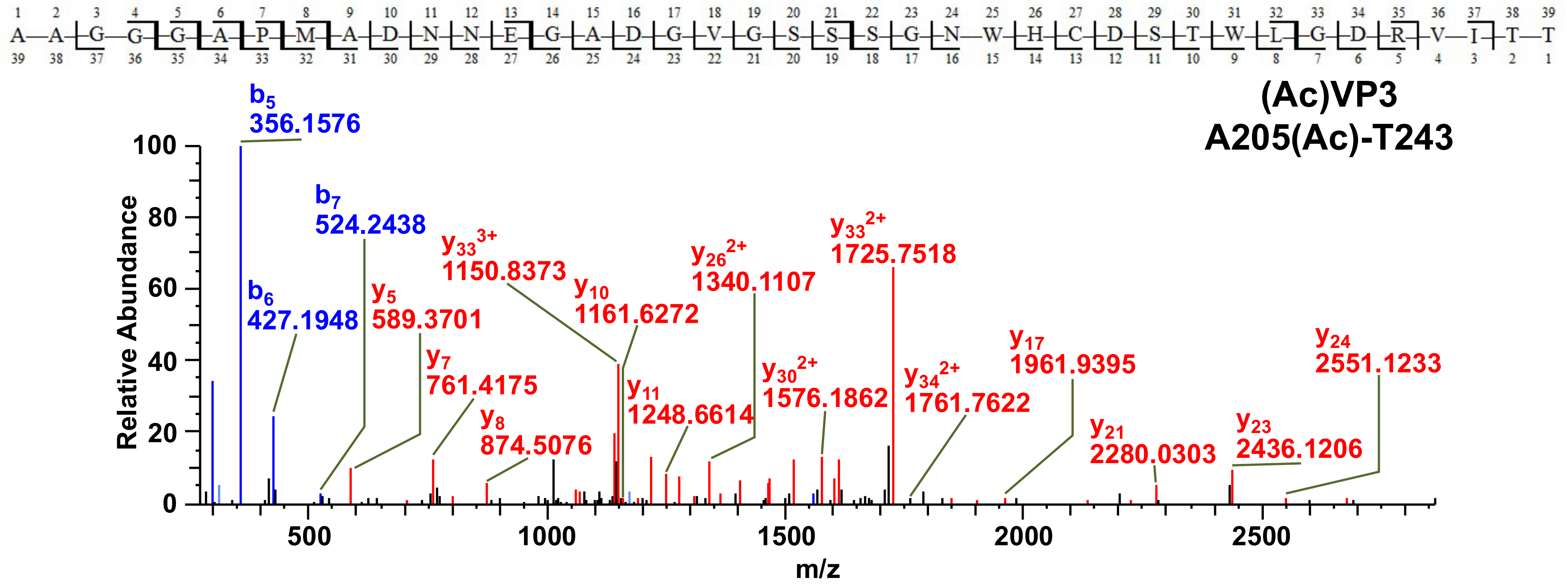

Figure S11 MS/MS spectra of the *AAGGGAPMADNNEGADGVGSSSGNWHCDSTWLGDRVIT* (A205-T242) peptide, with no acetylation, from peptide mapping of full AAV8 capsids, as identified using BPF 5.1. Peptide is an example of the detected peptides that indicate the presence of the VP3 capsid protein without N-term acetylation identified during intact mass analysis (Table S8)

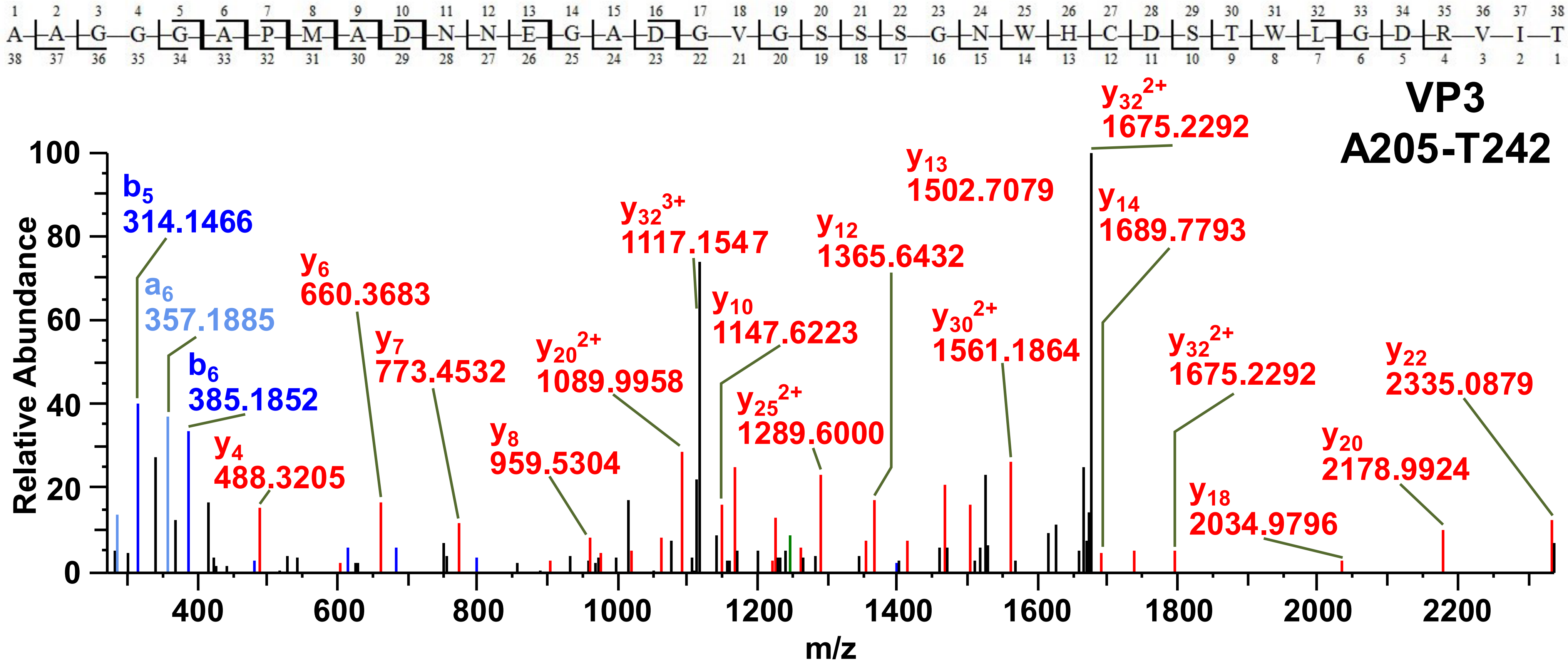

Figure S12 MS/MS spectra of the *ADNNEGADGVGSSSGNWHCDSTWLGDRVITTSTRT* (A213-T247) peptide, with acetylation at A213, from peptide mapping of full AAV8 capsids, as identified using BPF 5.1. Peptide is an example of the detected peptides that indicate the presence of the (Ac)VP3 Variant capsid protein identified during intact mass analysis (Table S8)

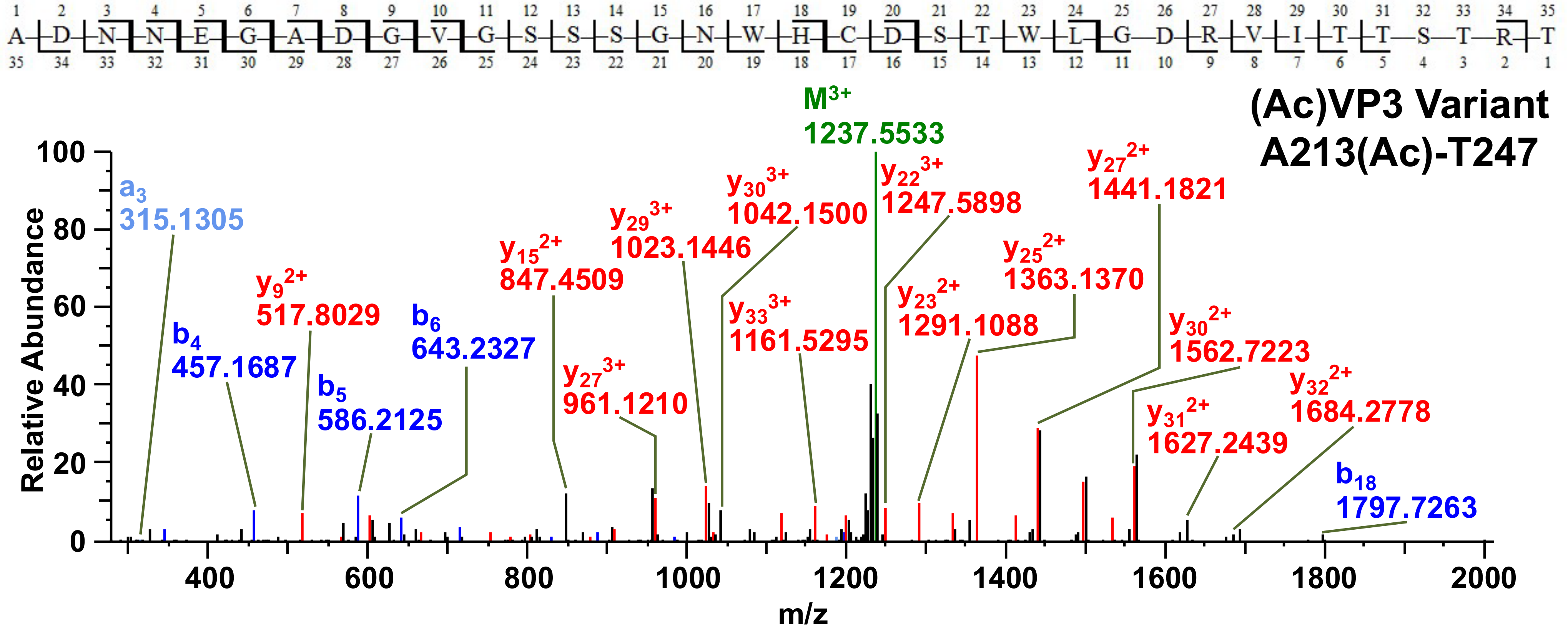

Figure S13 MS/MS spectra of the *VEEGAKTAPGKKRPVEPSPQRSPDSSTGIGKKGQQPARKRLNFGQTGDSESVPD* (V132-D185) peptide, with phosphorylation around S149 (exact location could not be determined), from peptide mapping of full AAV8 capsids, as identified using BPF 5.1. Peptide is an example of the detected peptides that indicate the presence of the V132-VP1 fragment identified during intact mass analysis (Table S8). The relatively poor spectrum is a result of the phosphorylation present which impacts digestion efficiency of the pepsin, resulting in the large peptide identified

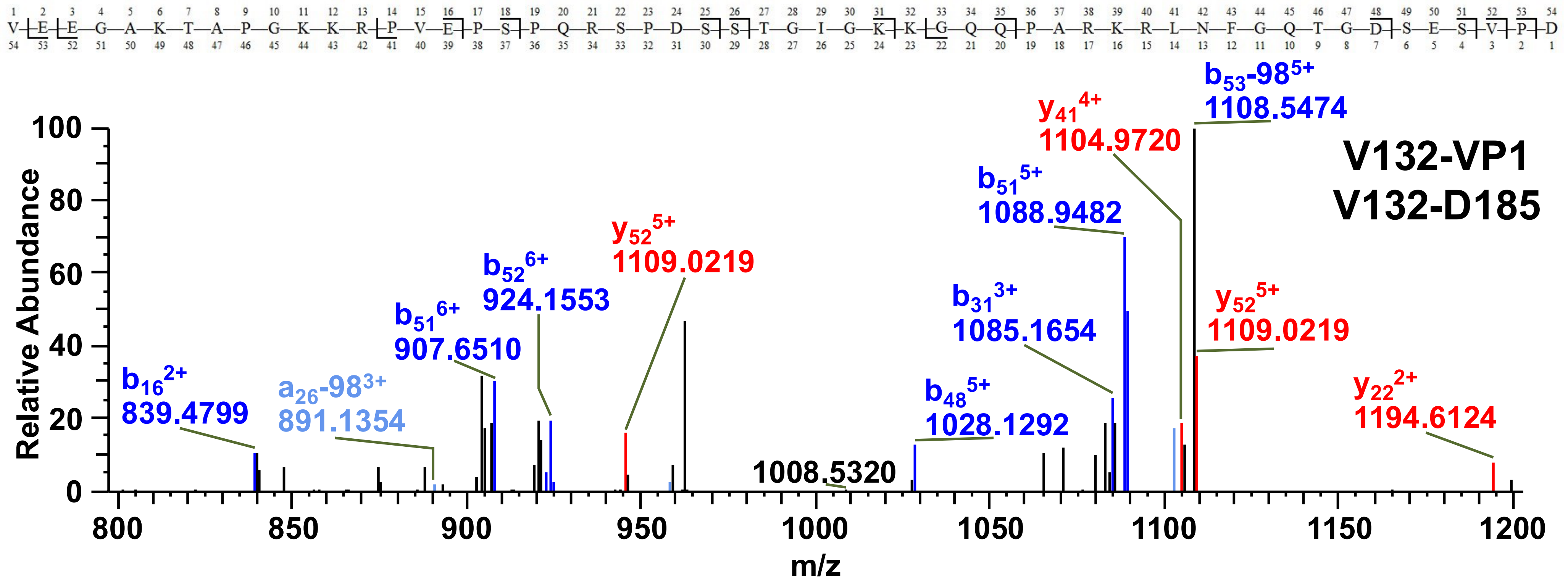

Figure S14 MS/MS spectra of the *GAPMADNNEGADGVGSSSGNWHCDSTWLGDRVIT* (G209-T242) peptide from peptide mapping of full AAV8 capsids, as identified using BPF 5.1. Peptide is an example of the detected peptides that indicate the presence of the G209-VP3 fragment identified during intact mass analysis (Table S8)

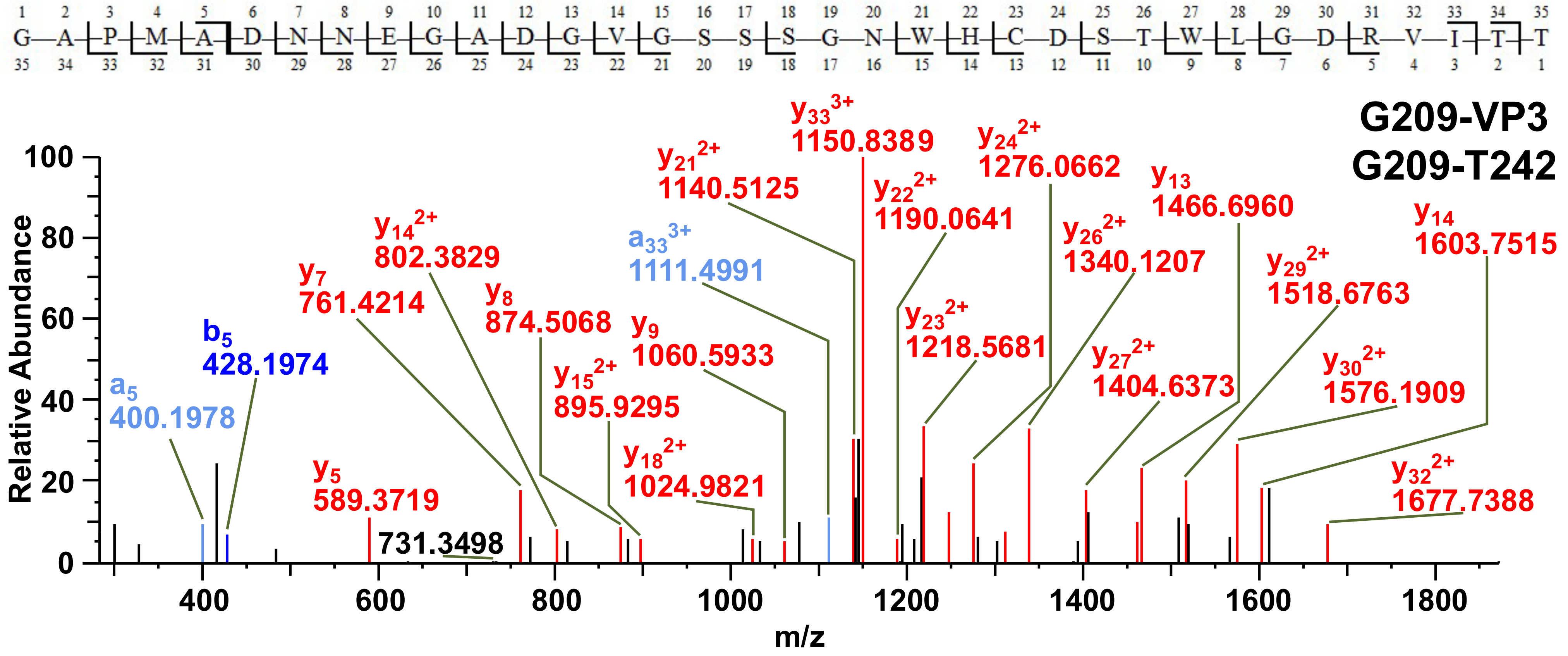

Figure S15 MS/MS spectra of the *AADGYLPDWLEDNLSEGIRE* (A2-E21) peptide, with acetylation at A2, from peptide mapping of full AAV9 capsids, as identified using BPF 5.1. Peptide is an example of the detected peptides that indicate the presence of the (Ac)VP1 capsid protein identified during intact mass analysis (Table S9)

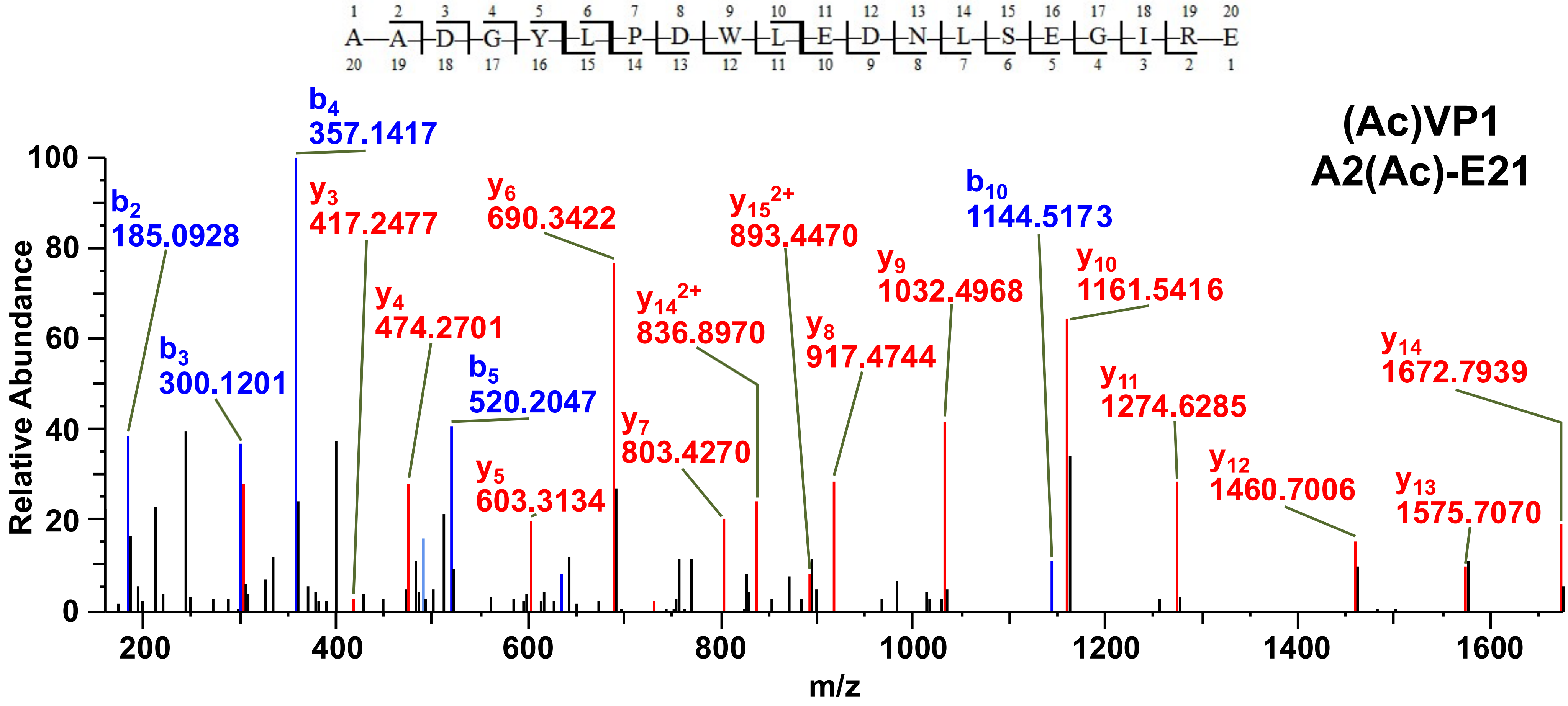

Figure S16 MS/MS spectra of the *APGKKRPVEQSPQEPDSSAGIGKSGAQPAKKRLNFGQTGDTESVPDPQPIGEPPAAPSGVGSLTM* (A139-M203) peptide, from peptide mapping of full AAV9 capsids, as identified using BPF 5.1. Peptide is an example of the detected peptides that indicate the presence of the VP2 capsid protein identified during intact mass analysis (Table S9)

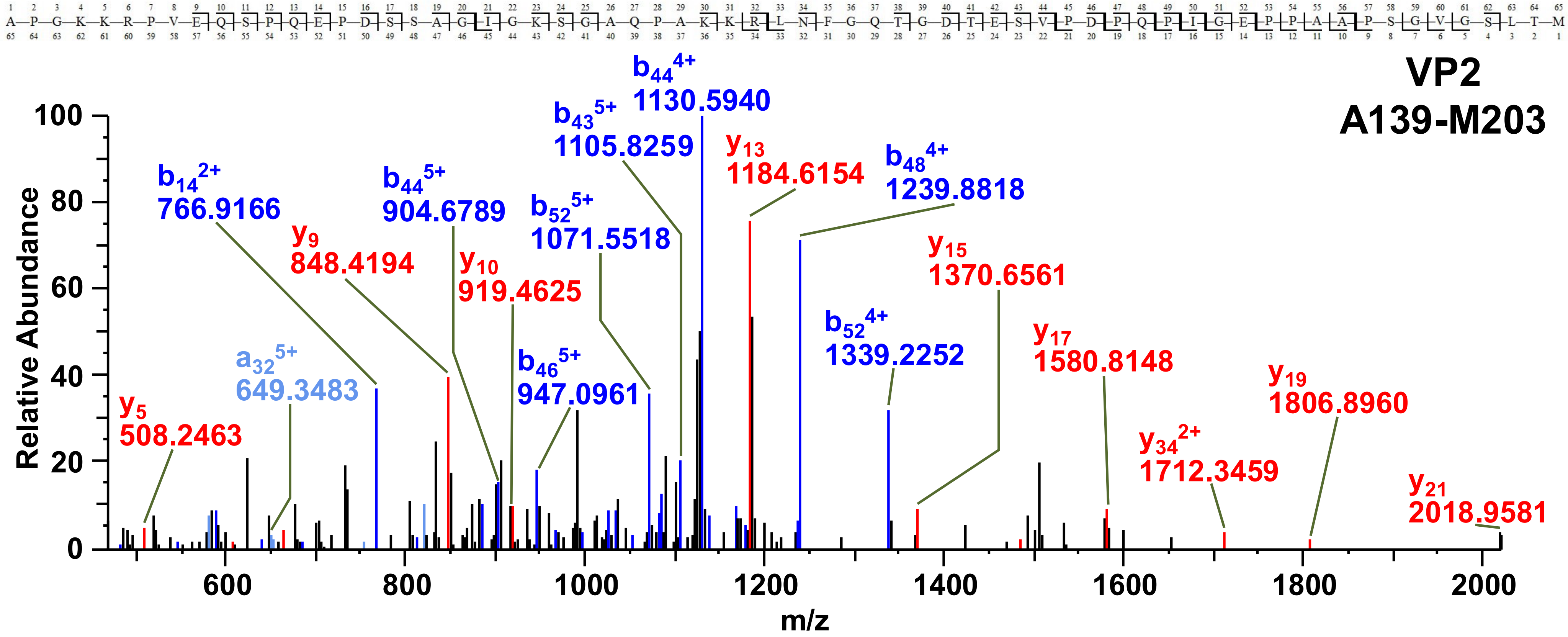

Figure S17 MS/MS spectra of the *ASGGGAPVADNNEGADGVGSSSGNWHCDSQWLGDRVIT* (A204-T241) peptide, with acetylation at A204, from peptide mapping of full AAV9 capsids, as identified using BPF 5.1. Peptide is an example of the detected peptides that indicate the presence of the (Ac)VP3 capsid protein identified during intact mass analysis (Table S9)

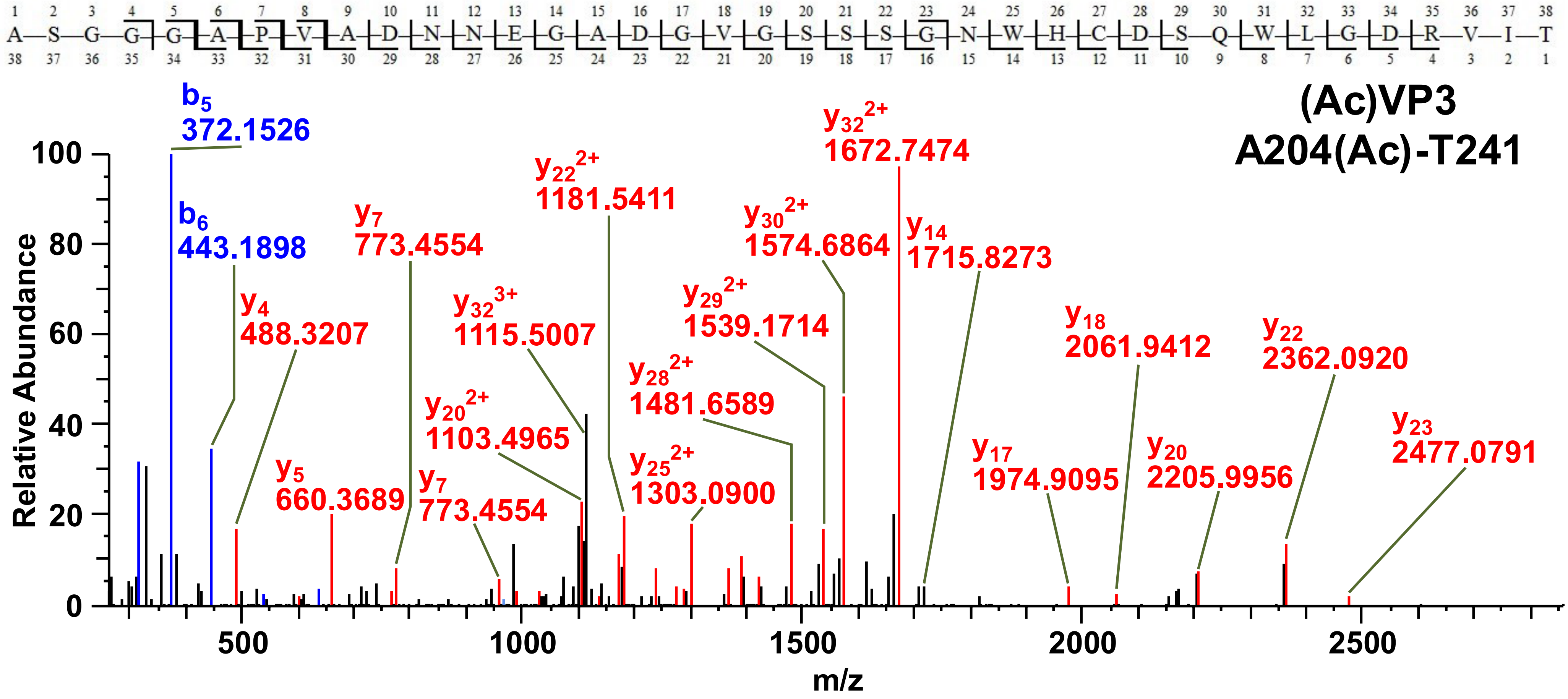

Figure S18 MS/MS spectra of the *MASGGGAPVADNNEGADGVGSSSGNWHCDSQWLGDRVITTSTRT* (M203-T246) peptide from peptide mapping of full AAV9 capsids, as identified using BPF 5.1. Peptide is an example of the detected peptides that indicate the presence of the M203-VP3 capsid protein identified during intact mass analysis (Table S9). This is a VP3 protein that didn't undergo expected N-term methionine cleavage and subsequent N-term acetylation

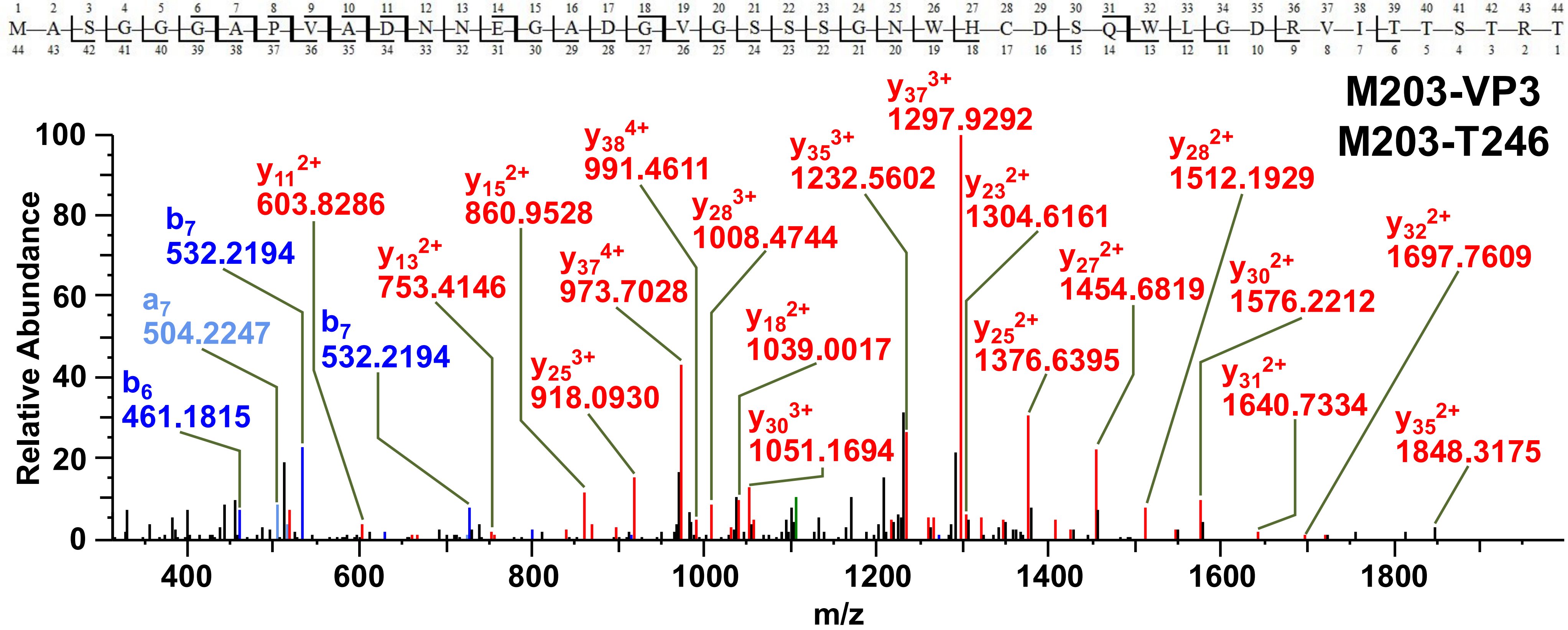

Figure S19 MS/MS spectra of the *RAVFQAKKRLLEPLGL* (R116-L131) peptide from peptide mapping of full AAV9 capsids, as identified using BPF 5.1. Peptide is an example of the detected peptides that indicate the presence of the R116-VP1 fragment identified during intact mass analysis (Table S9)

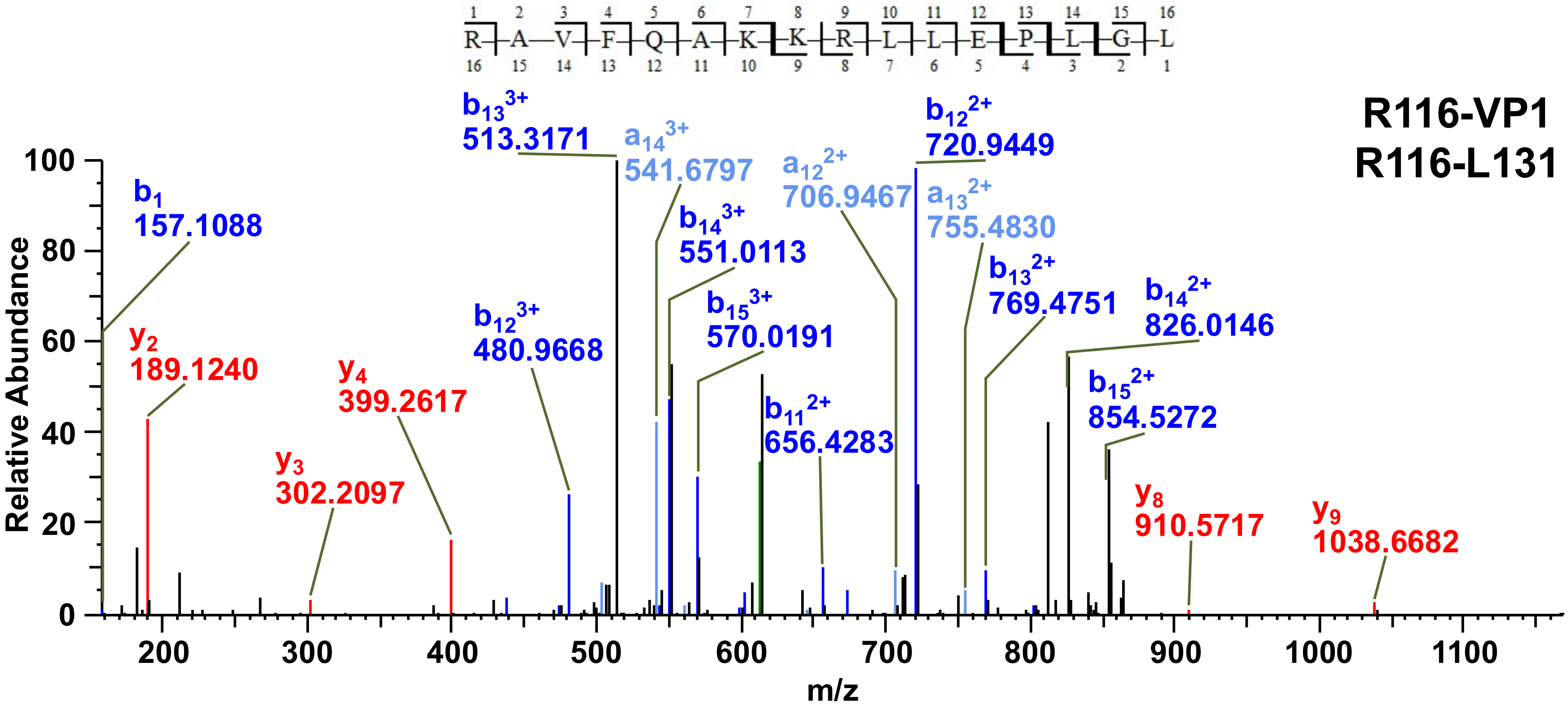

Figure S20 MS/MS spectra of the *LVEEAAKTAPGKKRPVE* (L131-E147) peptide from peptide mapping of full AAV9 capsids, as identified using BPF 5.1. Peptide is an example of the detected peptides that indicate the presence of the L131-VP1 fragment identified during intact mass analysis (Table S9)

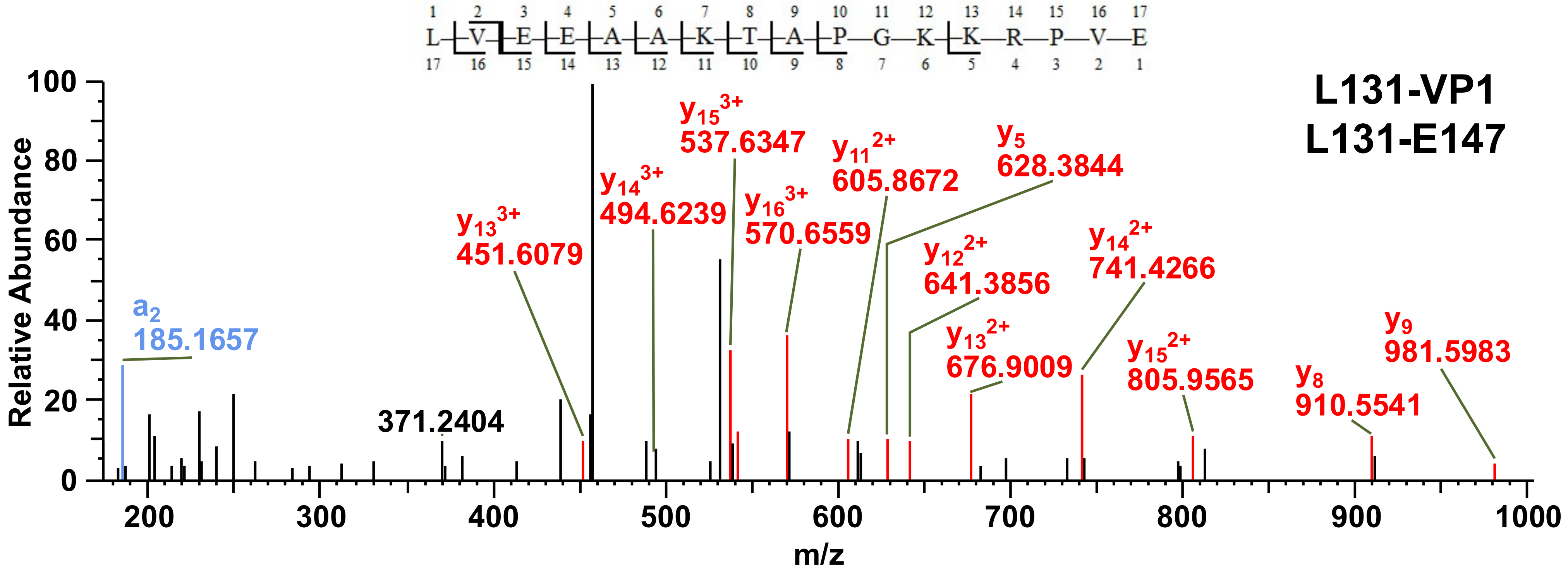

Figure S21 MS/MS spectra of the *FGQTGDTESVPDPQPIGEPPAAPSGVGSL* (F173-L201) peptide from peptide mapping of full AAV9 capsids, as identified using BPF 5.1. Peptide is an example of the detected peptides that indicate the presence of the F173-VP2 fragment identified during intact mass analysis (Table S9)

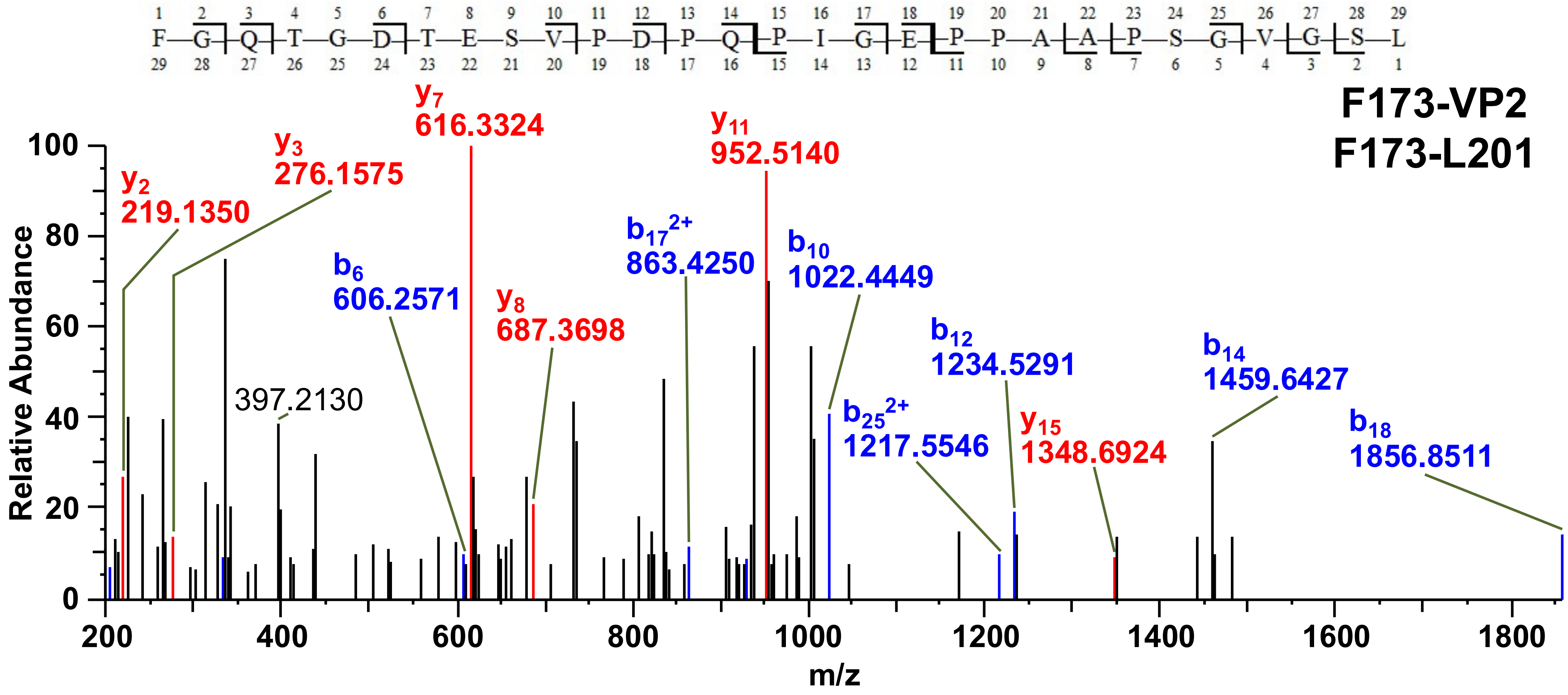

Figure S22 MS/MS spectra of the *IKNTPVPAD* (I649-D657) peptide from peptide mapping of full AAV9 capsids, as identified using BPF 5.1. Peptide is an example of the detected peptides that indicate the presence of the (Ac)VP3-D657 fragment identified during intact mass analysis (Table S9)

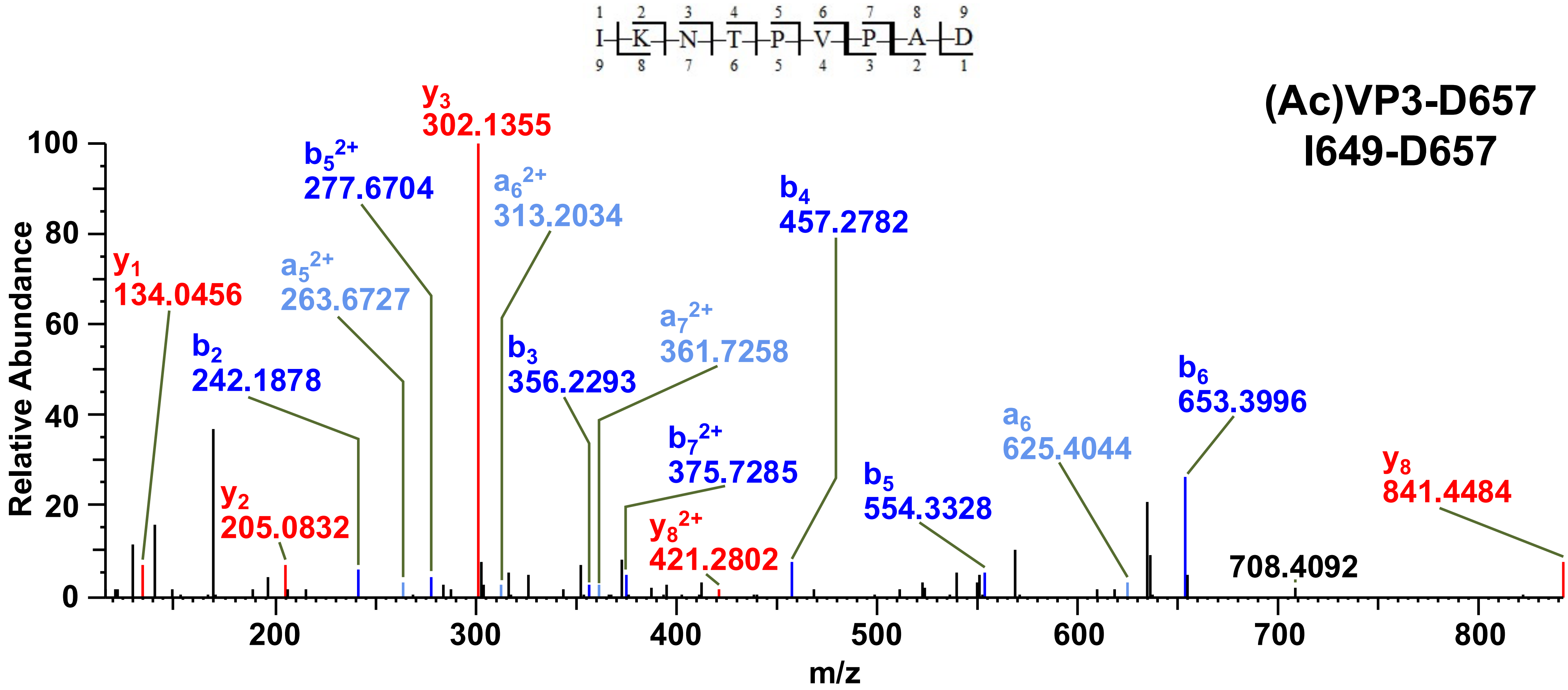

Figure S23 MS/MS spectra of the *ALNGRNSLMNPGPAMASHKEGEDRFFPLS* (A510-S538) peptide from peptide mapping of full AAV9 capsids, as identified using BPF 5.1. Peptide is an example of the detected peptides that indicate the presence of the (Ac)VP3-S538 fragment identified during intact mass analysis (Table S9)

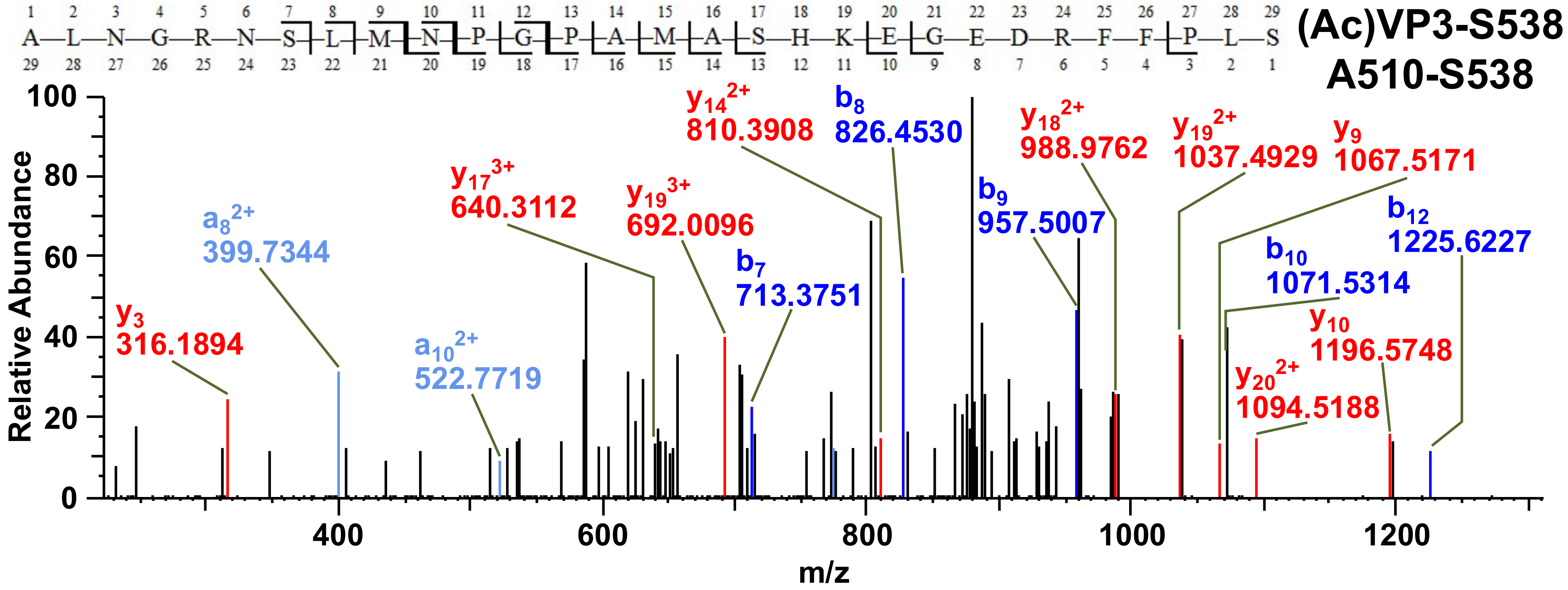

Figure S24 MS/MS spectra of the *FAWPGASSWALNGRNSLM* (F501-M518) peptide from peptide mapping of full AAV9 capsids, as identified using BPF 5.1. Peptide is an example of the detected peptides that indicate the presence of the (Ac)VP3-M518 fragment identified during intact mass analysis (Table S9)

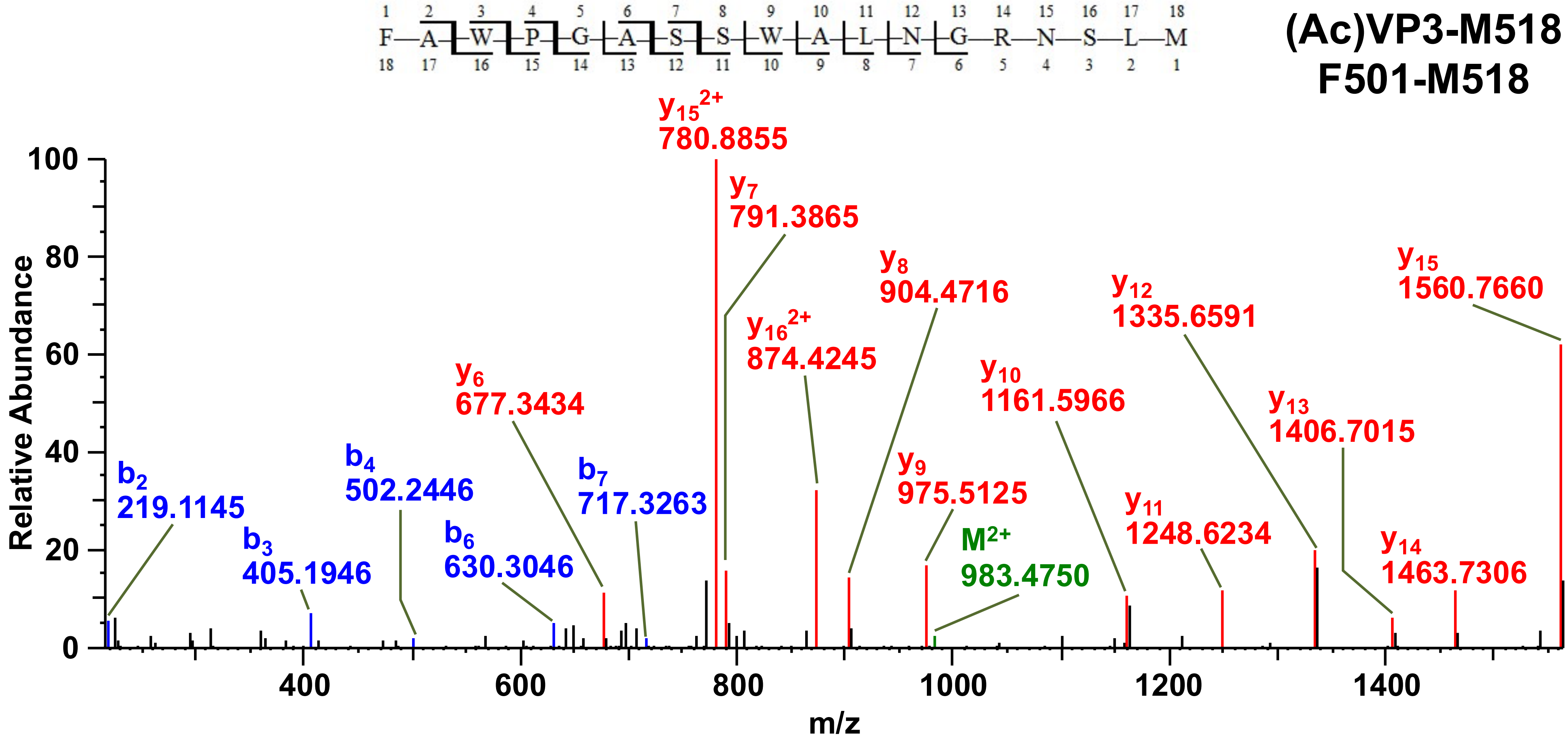

Figure S25 MS/MS spectra of the *AVQGRNYIPGPSYRQQRVSTTVTQNNNSEFAWPGASSWALN* (A472-N512) peptide from peptide mapping of full AAV9 capsids, as identified using BPF 5.1. Peptide is an example of the detected peptides that indicate the presence of the (Ac)VP3-N512 fragment identified during intact mass analysis (Table S9)

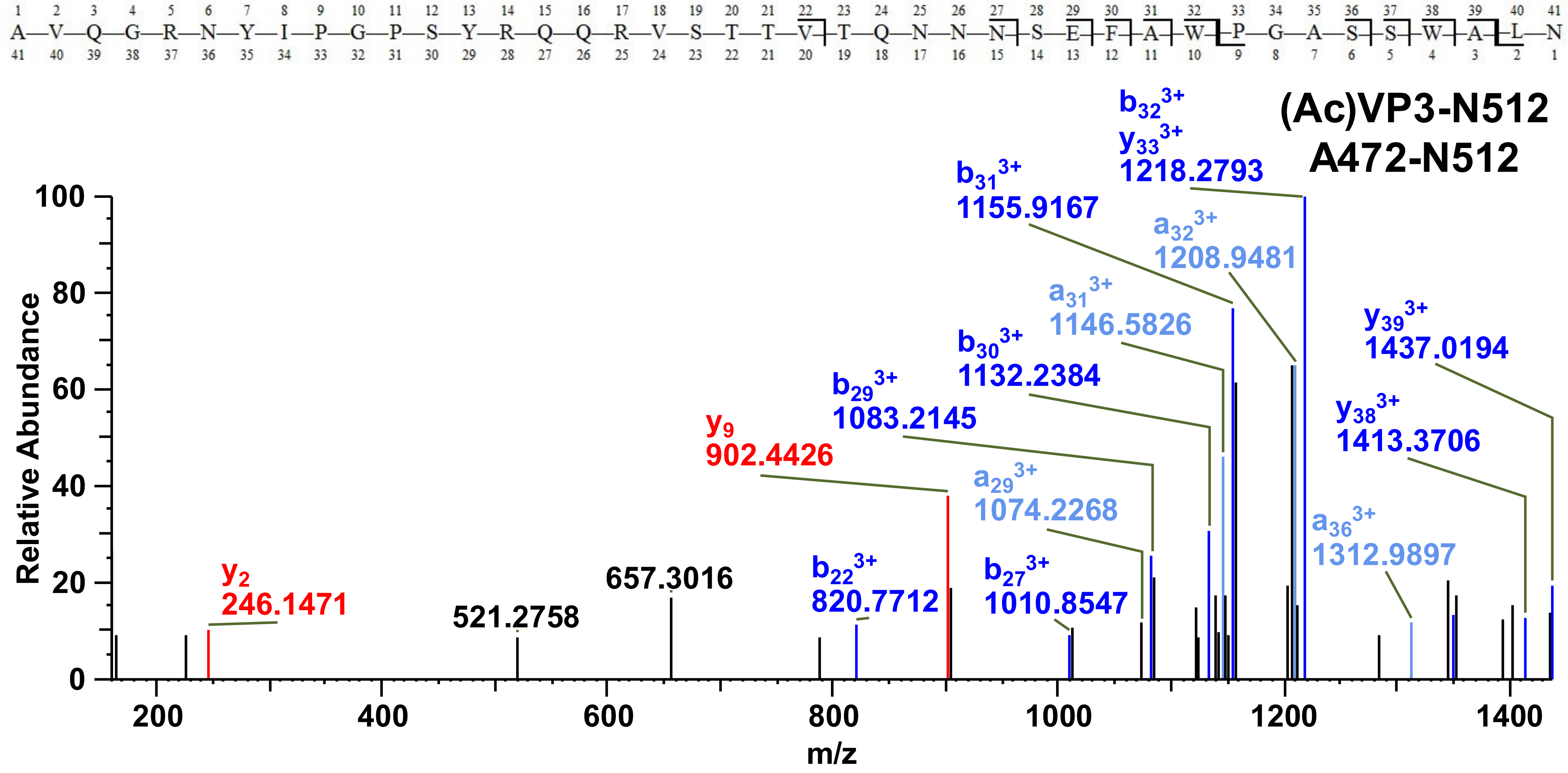

Figure S26 MS/MS spectra of the *YLYYLS* (Y443-S448) peptide from peptide mapping of full AAV9 capsids, as identified using BPF 5.1. Peptide is an example of the detected peptides that indicate the presence of the (Ac)VP3-S448 fragment identified during intact mass analysis (Table S9)

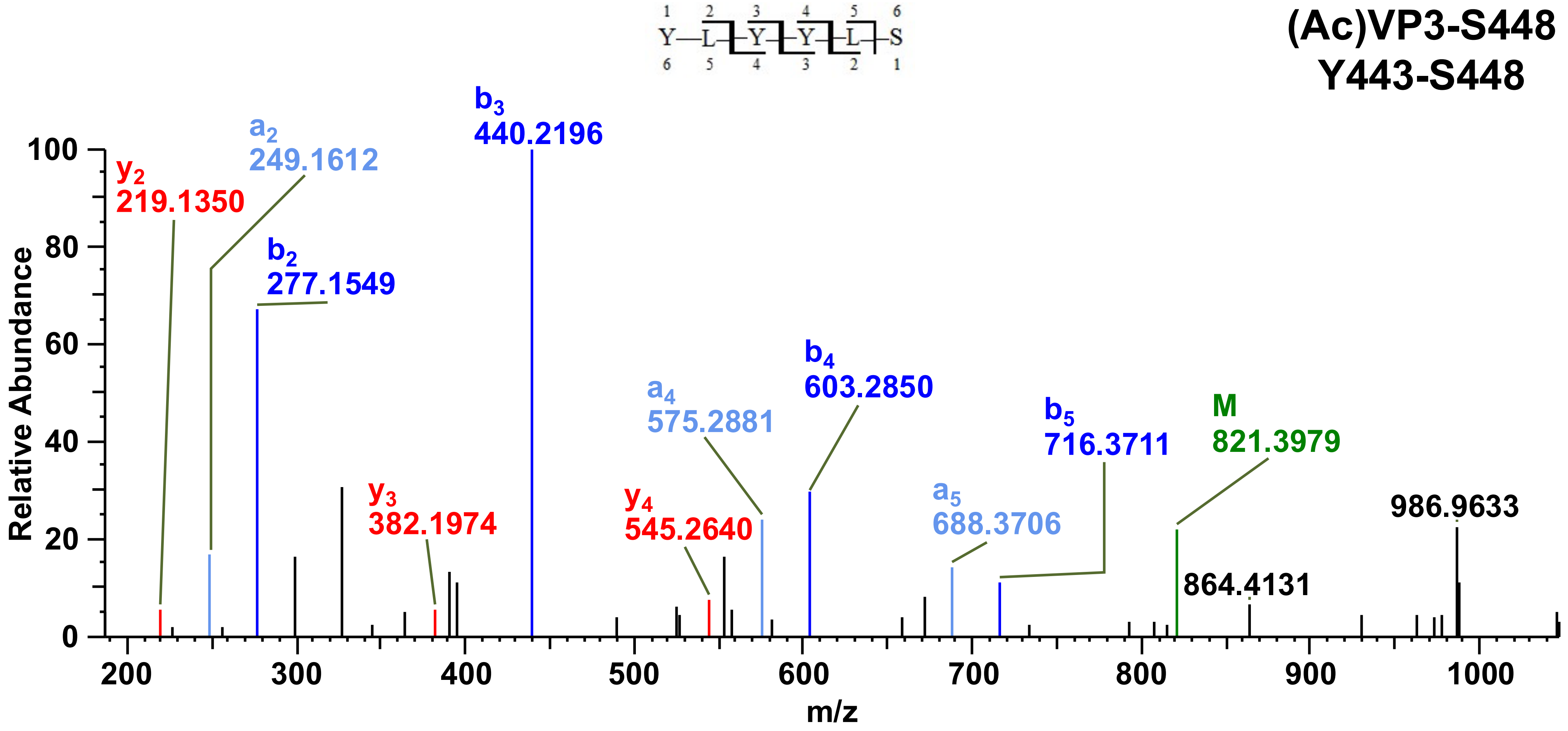

Supplement: Supplementary file 1 — Supplementary file1 (PDF 11225 KB) [file 216_2023_5097_MOESM1_ESM.pdf]
